# Mosaic integration and knowledge transfer of single-cell multimodal data with MIDAS

**DOI:** 10.1038/s41587-023-02040-y

**Published:** 2024-01-23

**Authors:** Zhen He, Shuofeng Hu, Yaowen Chen, Sijing An, Jiahao Zhou, Runyan Liu, Junfeng Shi, Jing Wang, Guohua Dong, Jinhui Shi, Jiaxin Zhao, Le Ou-Yang, Yuan Zhu, Xiaochen Bo, Xiaomin Ying

**Affiliations:** 1 Center for Computational Biology, Beijing Institute of Basic Medical Sciences, Beijing, China; 2 College of Electronics and Information Engineering, Shenzhen University, Shenzhen, China; 3 School of Automation, China University of Geosciences, Wuhan, China; 4 Institute of Health Service and Transfusion Medicine, Beijing, China

**Keywords:** Machine learning, Bioinformatics, Data integration

## Abstract

Integrating single-cell datasets produced by multiple omics technologies is essential for defining cellular heterogeneity. Mosaic integration, in which different datasets share only some of the measured modalities, poses major challenges, particularly regarding modality alignment and batch effect removal. Here, we present a deep probabilistic framework for the mosaic integration and knowledge transfer (MIDAS) of single-cell multimodal data. MIDAS simultaneously achieves dimensionality reduction, imputation and batch correction of mosaic data by using self-supervised modality alignment and information-theoretic latent disentanglement. We demonstrate its superiority to 19 other methods and reliability by evaluating its performance in trimodal and mosaic integration tasks. We also constructed a single-cell trimodal atlas of human peripheral blood mononuclear cells and tailored transfer learning and reciprocal reference mapping schemes to enable flexible and accurate knowledge transfer from the atlas to new data. Applications in mosaic integration, pseudotime analysis and cross-tissue knowledge transfer on bone marrow mosaic datasets demonstrate the versatility and superiority of MIDAS. MIDAS is available at https://github.com/labomics/midas.

## Abstract (web summary)

Single-cell, multiomic datasets are integrated using dimensionality reduction, imputation and batch correction.

## Main

Recently emerged single-cell multimodal omics (scMulti-omics) sequencing technologies enable the simultaneous detection of multiple modalities, such as RNA expression, protein abundance and chromatin accessibility, in the same cell^1,2^. These technologies, including the trimodal DOGMA-seq^3^ and TEA-seq^4^ and bimodal CITE-seq^5^ and ASAP-seq^3^, among many others^6–11^, reveal not only cellular heterogeneity at multiple molecular layers, enabling more refined identification of cell characteristics, but also connections across omes, providing a systematic view of ome interactions and regulation at single-cell resolution. The involvement of more measured modalities in analyses of biological samples increases the potential for enhancing the understanding of mechanisms underlying numerous processes, including cell functioning, tissue development and disease occurrence. The growing size of scMulti-omics datasets necessitates the development of new computational tools to integrate massive high-dimensional data generated from different sources, thereby facilitating more comprehensive and reliable downstream analysis for knowledge mining^1,2,12^. Such ‘integrative analysis’ also enables the construction of a large-scale single-cell multimodal atlas, which is urgently needed to make full use of publicly available single-cell multimodal data. Such an atlas can serve as an encyclopedia, allowing researchers the ability to transfer knowledge to their new data and in-house studies^13–15^.

Several methods for single-cell multimodal integration have recently been presented. Most of them have been proposed for the integration of bimodal data^15–23^. Fewer trimodal integration methods have been developed. MOFA+^24^ has been proposed for trimodal integration with complete modalities, and GLUE^25^ has been developed for the integration of unpaired trimodal data (that is, datasets involving single specific modalities).

All of these current integration methods have difficulty in handling flexible omics combinations. Due to the diversity of scMulti-omics technologies, datasets from different studies often include heterogeneous omics combinations with one or more missing modalities, resulting in mosaic-like data. The mosaic-like data are increasing rapidly and are predictably prevalent. Mosaic integration methods are urgently needed to markedly expand the scale and modalities of integration, breaking through the modality scalability and cost limitations of existing scMulti-omics sequencing technologies. Most recently, scVAEIT^26^, scMoMaT^27^, StabMap^28^ and Multigrate^29^ have been proposed to tackle this problem. However, these methods are not capable of aligning modalities or correcting batches, which results in limited functions and performances. Therefore, flexible and general multimodal mosaic integration remains challenging^30–32^. One major challenge is the reconciliation of modality heterogeneity and technical variation across batches. Another challenge is the achievement of modality imputation and batch correction for downstream analysis.

To overcome these challenges, we developed a probabilistic framework, MIDAS, for the mosaic integration and knowledge transfer of single-cell multimodal data. By using self-supervised learning^33^ and information-theoretic approaches^34^, MIDAS simultaneously achieves modality alignment, imputation and batch correction for single-cell trimodal mosaic data. We further designed transfer learning and reciprocal reference mapping schemes tailored to MIDAS to enable knowledge transfer. Systematic benchmarks and case studies demonstrate that MIDAS can accurately and robustly integrate mosaic datasets. Through the atlas-level mosaic integration of trimodal human peripheral blood mononuclear cell (PBMC) data, MIDAS achieved flexible and accurate knowledge transfer for various types of unimodal and multimodal query datasets. We also applied MIDAS to mosaic datasets of human bone marrow mononuclear cells (BMMCs) and demonstrated the satisfactory performance of MIDAS for mosaic data-based pseudotime analysis and cross-tissue knowledge transfer.

## Results

### The MIDAS model

**Fig. 1:**
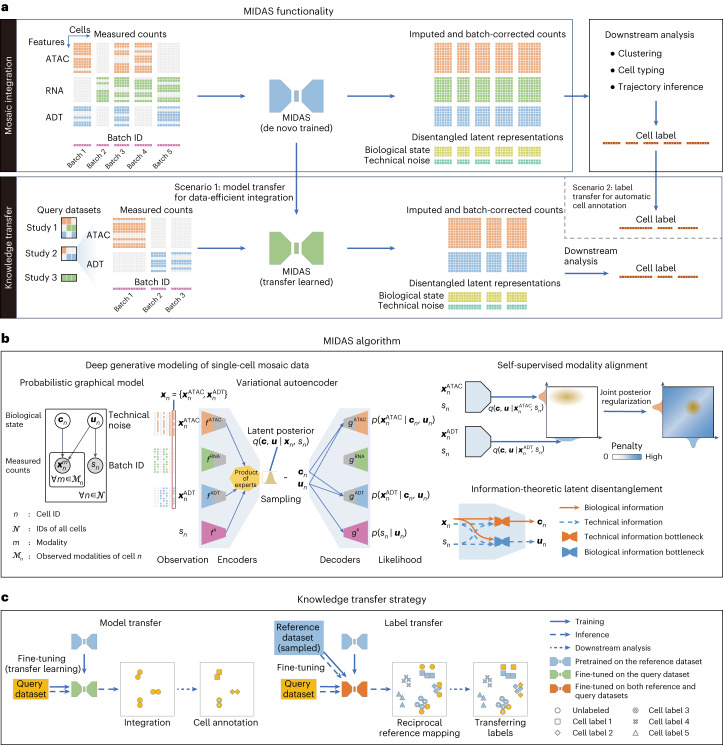
Overview of the MIDAS framework. **a**, Functionality of the MIDAS framework. **b**, MIDAS assumes that each cell’s measured counts and batch ID are generated from biological state and technical noise latent variables and uses the VAE to implement model learning and latent variable inference. Self-supervised learning is used to align different modalities on latent space through joint posterior regularization, and information-theoretic approaches help disentangle the latent variables. **c**, Two strategies are developed for MIDAS to achieve reference-to-query knowledge transfer, where model transfer uses a pretrained model for data-efficient integration, and label transfer reciprocally maps the reference and query datasets onto the latent space for automatic cell annotation.

MIDAS is a deep generative model^35,36^ that represents the joint distribution of incomplete single-cell multimodal data with assay for transposase-accessible chromatin (ATAC), RNA and antibody-derived tags (ADT) measurements. MIDAS assumes that each cell’s multimodal measurements are generated from two modality-agnostic and disentangled latent variables (the biological state (that is, cellular heterogeneity) and technical noise (that is, unwanted variation induced by single-cell experimentation)) through deep neural networks^37^. Its input consists of a mosaic feature-by-cell count matrix comprising different single-cell samples (batches) and a vector representing the cell batch IDs (Fig. 1a). The batches can derive from different experiments or be generated by the application of different sequencing techniques (for example, single-cell RNA-sequencing (scRNA-seq)^38^, CITE-seq^5^, ASAP-seq^3^ and TEA-seq^4^) and thus can have different technical noise, modalities and features. The output of MIDAS comprises biological state and technical noise matrices, which are the two low-dimensional representations of different cells, and an imputed and batch-corrected count matrix in which modalities and features missing from the input data are interpolated and batch effects are removed. These outputs can be used for downstream analyses, such as clustering, cell typing and trajectory inference^39^.

MIDAS is based on a variational autoencoder (VAE)^40^ architecture, with a modularized encoder network designed to handle the mosaic input data and infer the latent variables and a decoder network that uses the latent variables to seed the generative process for the observed data (Fig. 1b and Supplementary Fig. 1). MIDAS uses self-supervised learning to align different modalities in latent space, improving cross-modal inference in downstream tasks, such as imputation and translation. Information-theoretic approaches are applied to disentangle the biological state and technical noise, enabling further batch correction. Combining these elements into our optimization objective, scalable learning and inference of MIDAS are simultaneously achieved by the stochastic gradient variational Bayes^41^, which also enables large-scale mosaic integration and atlas construction of single-cell multimodal data. For the robust transfer of knowledge from the constructed atlas to query datasets with various modality combinations, transfer learning and reciprocal reference mapping schemes were developed for the transfer of model parameters and cell labels, respectively (Fig. 1c).

### MIDAS enables accurate trimodal rectangular integration

To compare MIDAS with state-of-the-art methods, we evaluated the performance of MIDAS in trimodal integration with complete modalities, a simplified form of mosaic integration, as few methods are designed specifically for trimodal mosaic integration. We named this task ‘rectangular integration’. We used two published single-cell trimodal human PBMC datasets (DOGMA-seq^3^ and TEA-seq^4^; Supplementary Table 1) with simultaneous RNA, ADT and ATAC measurements for each cell to construct dogma-full and teadog-full datasets. The dogma-full dataset took all four batches (LLL_Ctrl, LLL_Stim, DIG_Ctrl and DIG_Stim) from the DOGMA-seq dataset, and the teadog-full dataset took two batches (W1 and W6) from the TEA-seq dataset and two batches (LLL_Ctrl and DIG_Stim) from the DOGMA-seq dataset (Supplementary Table 2). Integration of each dataset requires the handling of batch effects and missing features and preservation of biological signals, which is challenging, especially for the teadog-full dataset, as the involvement of more datasets amplifies biological and technical variation.

**Fig. 2:**
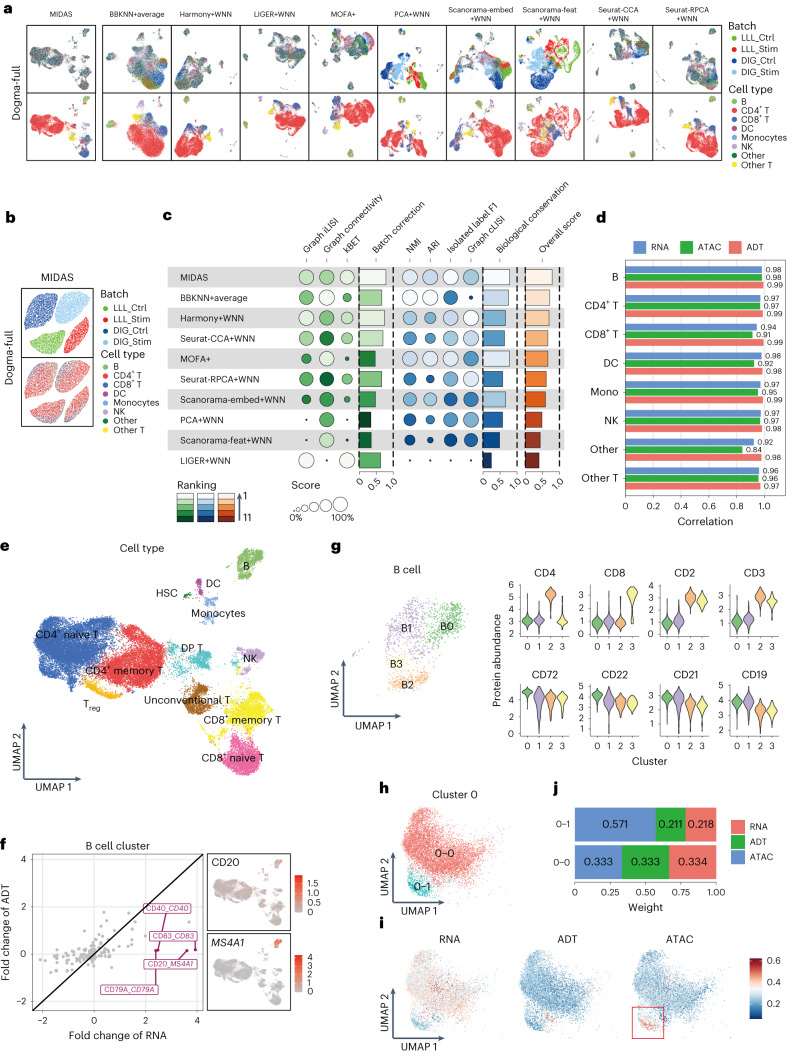
Evaluation and downstream analysis results obtained with MIDAS on rectangular integration tasks. **a**, UMAP visualization of cell embeddings obtained by MIDAS and nine other strategies in the dogma-full dataset. The left two graphs show inferred latent biological states, and the right graphs show dimensionality reduction results obtained with the other strategies; Mono, monocytes. **b**, UMAP visualization of latent technical noise inferred by MIDAS in the dogma-full dataset. **c**, scIB benchmarking of performance on the dogma-full rectangular integration task. **d**, Correlation of fold changes in gene/protein abundance and chromatin accessibility between raw and batch-corrected data. **e**, UMAP visualization of the inferred latent biological states with manually annotated cell types; DP, double positive; T_reg_, regulatory T cells. **f**, Expression inconsistencies between proteins and their corresponding genes in B cells. The left graph shows RNA and ADT fold changes, and the right graph shows the UMAP visualization of imputed CD20 and *MS4A1* expression. **g**, UMAP visualization of B cell subclusters (left) and violin plots of imputed protein abundance across subclusters (right). **h**, UMAP plot of CD4^+^ naive T cell C0-0 and C0-1 subclusters from the dogma dataset. **i**, Single-cell modality contributions to C0 clustering. The red rectangle highlights the greater contribution of the ATAC modality in cluster C0-1. **j**, Modality contributions to the integrated clustering of C0-0 and C0-1 cells.

Uniform manifold approximation and projection (UMAP)^42^ visualization showed that the biological states of different batches were well aligned, their grouping was consistent with the cell-type labels (Fig. 2a, left, and Supplementary Fig. 2a, left) and the technical noise was grouped by batch and exhibited little relevance to cell types (Fig. 2b and Supplementary Fig. 2b). Thus, the two inferred latent variables were disentangled well and independently represented biological and technical variation.

Taking the inferred biological states as low-dimensional representations of the integrated data, we compared the performance of MIDAS with that of nine strategies derived from recently published methods (Methods and Supplementary Table 3) in the removal of batch effects and preservation of biological signals. UMAP visualization of the integration results showed that MIDAS ideally removed batch effects and also preserved cell-type information on both dogma-full and teadog-full datasets, whereas the performance of other strategies was not satisfactory. For example, BBKNN+average, MOFA+, PCA+WNN, Scanorama-embed+WNN and Scanorama-feat+WNN did not mix different batches well, and PCA+WNN and Scanorama-feat+WNN produced cell clusters largely inconsistent with cell types (Fig. 2a and Supplementary Fig. 2a).

In a quantitative evaluation of the low-dimensional representations of different strategies performed with the widely used single-cell integration benchmarking (scIB)^43^ tool, MIDAS had the highest batch correction, biological conservation and overall scores for the dogma-full and teadog-full datasets (Fig. 2c and Supplementary Fig. 2c). In addition, MIDAS preserved cell-type-specific patterns in batch-corrected RNA, ADT and ATAC data (Methods). For each cell type, fold changes in gene/protein abundance and chromatin accessibility in raw and batch-corrected data correlated strongly and positively (all Pearson’s *r* > 0.8; Fig. 2d).

Manual cell clustering and typing based on the integrated low-dimensional representations and batch-corrected data from MIDAS led to the identification of 13 PBMC types, including B cells, T cells, dendritic cells (DCs), natural killer (NK) cells and monocytes (Fig. 2e). We identified a distinct T cell cluster that highly expresses CD4 and CD8 simultaneously. We labeled this cluster as double-positive CD4^+^CD8^+^ T cells. This phenomenon was also reported in previous studies^44^. Another T cell cluster, containing mucosa-associated invariant T cells and γδ T cells, was distinct from conventional T cells and was labeled unconventional T cells^45^.

As is known, multiple omes regulate biological functions synergistically^1,2^. MIDAS integrates RNA, ADT and ATAC single-cell data and hence facilitates the discovery of the intrinsic nature of cell activities in a more comprehensive manner. We found that all omics data contributed greatly to the identification of cell types and functions (Supplementary Fig. 3).

Systematic screening for expression inconsistencies between proteins and their corresponding genes, expected to reflect ome irreplaceability, at the RNA and ADT levels demonstrated that several markers in each cell type were expressed strongly in one modality and weakly in the other (Fig. 2f and Supplementary Fig. 4). For instance, *MS4A1*, which encodes a B cell-specific membrane protein, was expressed extremely specifically in B cells, but the CD20 protein encoded by *MS4A1* was rarely detected, confirming the irreplaceability of the RNA modality. We also found that ADT could complement RNA-based clustering. For example, the simultaneous expression of T cell markers (CD3 and CD4) was unexpectedly observed in two subclusters of B cells (B2 and B3) expressing canonical B cell makers (CD19, CD21 and CD22; Fig. 2g). As this phenomenon could not be replicated using RNA data alone, this finding confirms the irreplaceability of the ADT modality. However, it should be noted that certain technical issues of single-cell sequencing may also lead to the emergence of these cells^46^.

Investigation of the uniqueness of chromatin accessibility in multiomics integration at the ATAC level showed that ATAC contributed more than did ADT and RNA to the integration of a subcluster of CD4^+^ naive T cells (Methods and Fig. 2h–j). We took the ratio of peak number of a cell to that of all cells as the representation of the cell accessibility level. RNA and ADT expression did not differ between these cells and their CD4^+^ naive T cell siblings, but lower accessibility levels were observed at the ATAC layer (<0.02; Supplementary Fig. 5). Gene Ontology enrichment analysis^47^ indicated that the inaccessible regions are related to T cell activation, cell adhesion and other immune functions. Therefore, we define this cluster as low chromatin-accessible (LCA) naive CD4^+^ T cells. Although this discovery needs to be verified further, it demonstrates the multiomics integration capability of MIDAS.

### MIDAS enables reliable trimodal mosaic integration

**Fig. 3:**
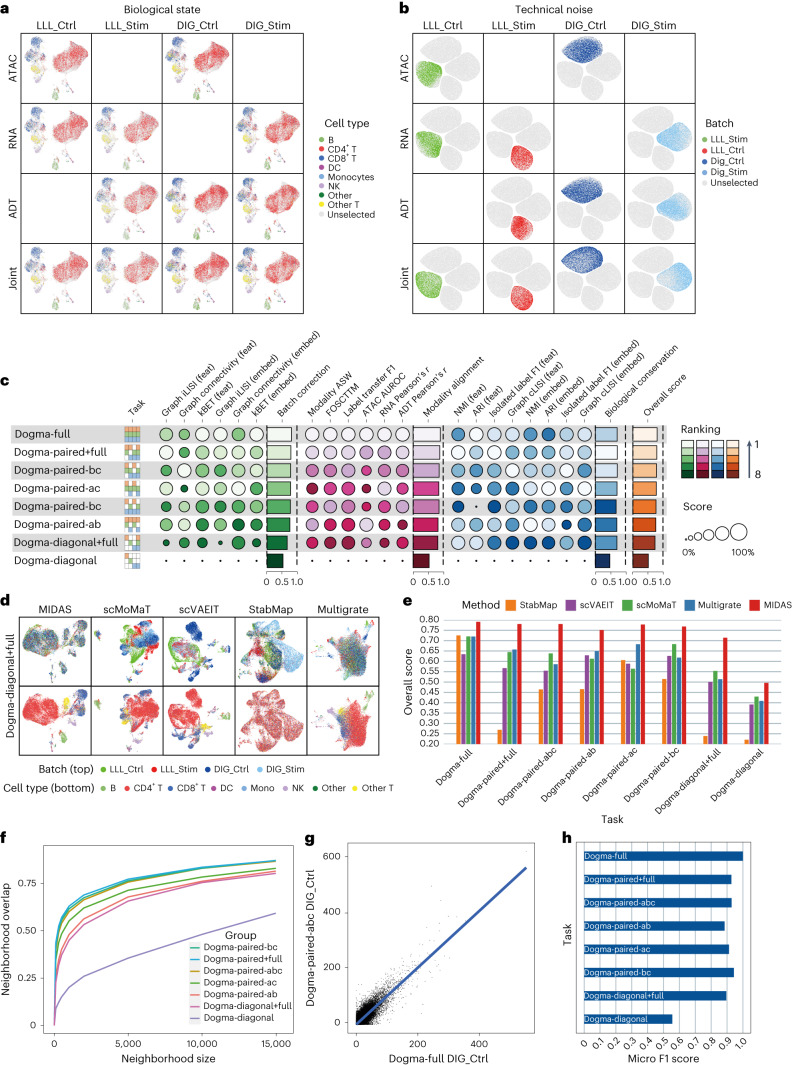
Qualitative and quantitative evaluation of MIDAS’s performance on mosaic integration tasks. **a**,**b**, UMAP visualization of the biological states (**a**) and technical noises (**b**) inferred by MIDAS on the dogma-paired-abc dataset. **c**, Benchmarking of MIDAS’s performance on dogma mosaic integration tasks using our proposed scMIB. **d**, UMAP comparison of embeddings on dogma-diagonal+full mosaic integration tasks. Cells in the top row are colored by batch, and cells in the bottom row are colored by cell type. **e**, Comparison of scIB overall scores on dogma mosaic integration tasks. **f**, Consistency of dimensional reduction results from different tasks with those from the dogma-full task measured by the overlap of cells’ nearest neighbors. **g**, Consistency of gene regulation links in inferred (dogma-paired-abc DIG_Ctrl batch) and raw (dogma-full DIG_Ctrl batch) RNA data. Values represent the regulation importance of gene–transcript factor pairs. **h**, Micro F1 scores reflecting the consistency of downstream-analyzed cell labels between mosaic tasks and the dogma-full task.

At present, trimodal sequencing techniques are still immature. Most of the existing datasets are unimodal or bimodal with various modality combinations. MIDAS is designed to integrate these diverse multimodal datasets, that is, mosaic datasets. To evaluate the performance of MIDAS on mosaic integration, we further constructed 14 incomplete datasets based on the previously generated rectangular datasets, including dogma-full and teadog-full datasets (Methods and Supplementary Table 2). Each mosaic dataset was generated by removing several modality batch blocks from the full-modality dataset. We then took the rectangular integration results as the baseline and examined whether MIDAS could obtain comparable results on mosaic integration tasks. We assessed the ability of MIDAS to perform batch correction, modality alignment and biological conservation. Here, we also focused on modality alignment because it guarantees accurate cross-modal inference for processes such as downstream imputation and knowledge transfer. For qualitative evaluation, we used UMAP to visualize the biological states and technical noises inferred from the individual and the joint input modalities (Fig. 3a,b and Supplementary Figs. 6 and 7). Taking the dogma-paired-abc dataset, for example, for each modality, the biological states were consistently distributed across different batches (Fig. 3a), whereas the technical noises were grouped by batches (Fig. 3b), indicating that the batch effects were well disentangled from the biological states. Similarly, the distributions of biological states and technical noises within batches were very similar across modalities (Fig. 3a,b), suggesting that MIDAS internally aligns different modalities in latent space. Moreover, the biological states of each cell type were grouped together, and the cell-type silhouettes were consistent across batches and modality combinations (Fig. 3a), reflecting robust conservation of the biological signals after mosaic integration.

To quantitatively evaluate MIDAS on mosaic integration, we proposed single-cell mosaic integration benchmarking (scMIB). scMIB extends scIB with modality alignment metrics and defines each type of metric on both embedding (latent) space and feature (observation) space, resulting in 20 metrics in total (Methods and Supplementary Table 4). The obtained batch correction, modality alignment, biological conservation and overall scores for paired+full, paired-abc, paired-ab, paired-ac, paired-bc and diagonal+full tasks performed with the dogma and teadog datasets were similar to those obtained with rectangular integration (Fig. 3c and Supplementary Fig. 8a). MIDAS showed moderate performance in the dogma- and teadog-diagonal tasks, likely due to the lack of cell-to-cell correspondence across modalities in these tasks, which can be remedied via knowledge transfer (see MIDAS enables knowledge transfer across mosaic datasets).

scIB benchmarking showed that MIDAS, when given incomplete datasets (paired+full, paired-abc, paired-ab, paired-ac and paired-bc for dogma and teadog), outperformed methods that rely on the full-modality datasets (dogma- and teadog-full; Supplementary Fig. 8b,c). Even with the severely incomplete dogma- and teadog-diagonal+full datasets, the performance of MIDAS surpassed that of most other methods.

We also compared MIDAS to scVAEIT, scMoMaT, Multigrate and StabMap (Methods), which can handle mosaic datasets. UMAP visualization of the low-dimensional cell embeddings showed that MIDAS removed batch effects and preserved biological signals well on various tasks, whereas the other four methods did not integrate trimodal data well, especially when missing modalities (dogma in Fig. 3d and Supplementary Fig. 9 and teadog in Supplementary Fig. 9). To be specific, MIDAS aligned the cells of different batches well and grouped them consistently with the cell-type labels, whereas the other methods did not mix different batches well and produced cell clusters largely inconsistent with cell types. scIB benchmarking showed that MIDAS had stable performance on different mosaic tasks, and its overall scores were much higher than those of the other methods (dogma in Fig. 3e, teadog in Supplementary Fig. 10 and detailed scores in Supplementary Fig. 11).

The identification of cells’ nearest neighbors based on individual dimensionality reduction results and comparison of neighborhood overlap among tasks showed that this overlap exceeded 0.75 for most tasks, except dogma-diagonal, when the number of neighbors reached 10,000 (Fig. 3f). As imputed omics data have been inferred to deteriorate the accuracy of gene regulatory inference in many cases^48^, we evaluated the consistency of downstream analysis results obtained with the performance of different mosaic integration tasks with the dogma datasets. We validated the conservation of gene regulatory networks in the imputed data. In the dogma-paired+full task, for example, the regulatory network predicted from imputed data was consistent with that predicted from the dogma-full data (Fig. 3g). These results indicate that the modality inference performed by MIDAS is reliable.

The MIDAS-based annotation of cell types for the mosaic integration tasks and computation of their confusion matrices and micro F1 scores showed that the cell-type labels generated from the incomplete datasets, except dogma-diagonal, were largely consistent with the dogma-full labels, with all micro F1 scores exceeding 0.885 (Fig. 3h and Supplementary Fig. 12). The separation of monocytes and DCs was difficult in some mosaic experiments, mainly because the latter originate from the former^49^ and likely also because the monocyte population in the dogma dataset was small.

To demonstrate the robustness of MIDAS for real-world mosaic integration, we tested MIDAS in more challenging cases, including batches with various sequencing depths, batches with inconsistent cell types and perturbations of hyperparameters (Supplementary Note 1, Supplementary Figs. 13–15 and Supplementary Tables 5 and 6). We compared MIDAS with other competing methods on more omics combinations and also benchmarked their computational costs (Supplementary Note 1 and Supplementary Figs. 16–18). All the results show that MIDAS is a robust, versatile and efficient tool for single-cell multimodal integration.

### MIDAS enables atlas-level mosaic integration of PBMC data

**Fig. 4:**
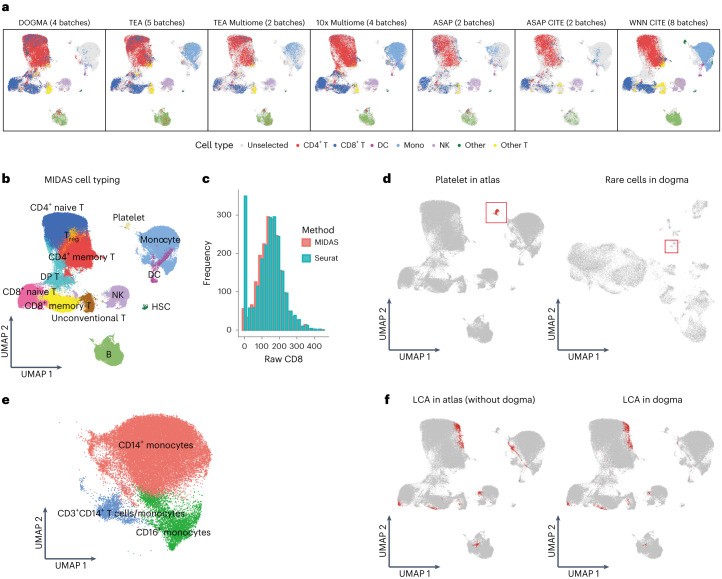
Atlas-level mosaic integration and downstream analysis results obtained with the application of MIDAS to trimodal PBMC data. **a**, UMAP visualization of the biological states inferred by MIDAS with the PBMC mosaic dataset across seven datasets. Cell-type labels are derived from Seurat labeling. **b**, Labels of atlas cells annotated based on clustering of MIDAS embeddings. **c**, The distributions of raw protein levels of CD8 among CD8^+^ cells labeled by MIDAS and Seurat, respectively, from the DIG_Ctrl batch of the DOGMA-seq dataset. **d**, Platelets in the atlas (left) and a rare cluster of platelet-like cells in the dogma (right) datasets. **e**, High-resolution clustering of monocyte types in the atlas. **f**, LCA cells (red) in the atlas. Left, all LCA cells except those from the dogma dataset; right, LCA cells from the dogma dataset.

We used MIDAS for the large-scale mosaic integration of 18 PBMC batches from bimodal sequencing platforms (for example, 10x Multiome, ASAP-seq and CITE-seq) and the 9 batches from the DOGMA-seq and TEA-seq trimodal datasets (a total of 27 batches from 10 platforms comprising 185,518 cells; Methods and Supplementary Table 1 and 9). Similar to the results obtained with the dogma-full and teadog-full datasets, MIDAS achieved satisfactory batch removal and biological conservation. UMAP visualization showed that the inferred biological states of different batches maintained a consistent PBMC population structure and conserved batch-specific (due mainly to differences in experimental design) biological information (Fig. 4a and Supplementary Figs. 19 and 20a). In addition, the technical noise was clearly grouped by batch (Supplementary Fig. 20b). These results suggest that the biological states and technical noises were disentangled well and that the data could be used reliably in downstream analysis.

Manual labeling of cell types according to cluster markers achieved largely consistent separation and annotation with automatic labeling by Seurat^15^, which indicates the reliability of MIDAS for constructing the atlas (Fig. 4b and Supplementary Fig. 20a). We also found that MIDAS labeling seems more biologically meaningful when we checked the CD8 protein level of CD8-labeled cells between the two labeling systems (MIDAS and Seurat; Fig. 4c). Consistent with the rectangular integration results (Fig. 2e), we identified all cell types known to be in the atlas, including B cells, conventional T cell subsets, double-positive T cells, NK cells, unconventional T cells and hematopoietic stem cells (HSCs), demonstrating the robustness of MIDAS. The integration of more datasets with MIDAS led to the identification of rare clusters and high-resolution cell typing. For example, a group of cells from the DOGMA-seq dataset aggregated into a much larger cluster with recognizable platelet markers in the PBMC atlas (Fig. 4d). Because platelets have no cell nuclei and are not expected to be present in the DOGMA-seq dataset, this rare group of cells could motivate researchers to perform further experiments to validate it. In addition, the atlas contained more monocyte subclusters, including CD14^+^, CD16^+^ and CD3^+^CD14^+^ monocytes, than obtained with rectangular integration (Fig. 4e). Other cell types present in more subclusters in the atlas included CD158e1^+^ NK cells, CD4^+^CD138^+^CD202b^+^ T cells and RTKN2^+^CD8^+^ T cells (Supplementary Fig. 21a).

Most batches in the atlas contained considerable numbers of LCA cells (Fig. 4f and Supplementary Fig. 21b) with an accessibility level of <0.02, as did the DOGMA-seq dataset (Fig. 2i). The chromatin accessibility levels of cells in the atlas showed an obvious bimodal distribution, reflecting the existence of two ATAC patterns (Supplementary Fig. 21b). CD8^+^ T cell, CD14^+^ monocyte, NK cell, B cell and other clusters contained LCA cells (Fig. 4b,f), implying that LCA is common in various cell types.

### MIDAS enables knowledge transfer across mosaic datasets

To investigate the knowledge transfer capability of MIDAS, we repartitioned the atlas dataset into reference (for atlas construction) and query (knowledge transfer target) datasets (Supplementary Table 9). By removing DOGMA-seq from the atlas, we obtained a reference dataset named atlas-no_dogma. To test the flexibility of knowledge transfer, we used DOGMA-seq to construct 14 query datasets: 1 rectangular and 7 mosaic trimodal datasets generated previously and 6 rectangular datasets with fewer modalities (Methods and Supplementary Table 10). In consideration of real applications, we defined model and label knowledge transfer scenarios (Methods). In the model transfer scenario, knowledge was transferred implicitly through model parameters via transfer learning. In the label transfer scenario, knowledge was transferred explicitly through cell labels via reference mapping.

**Fig. 5:**
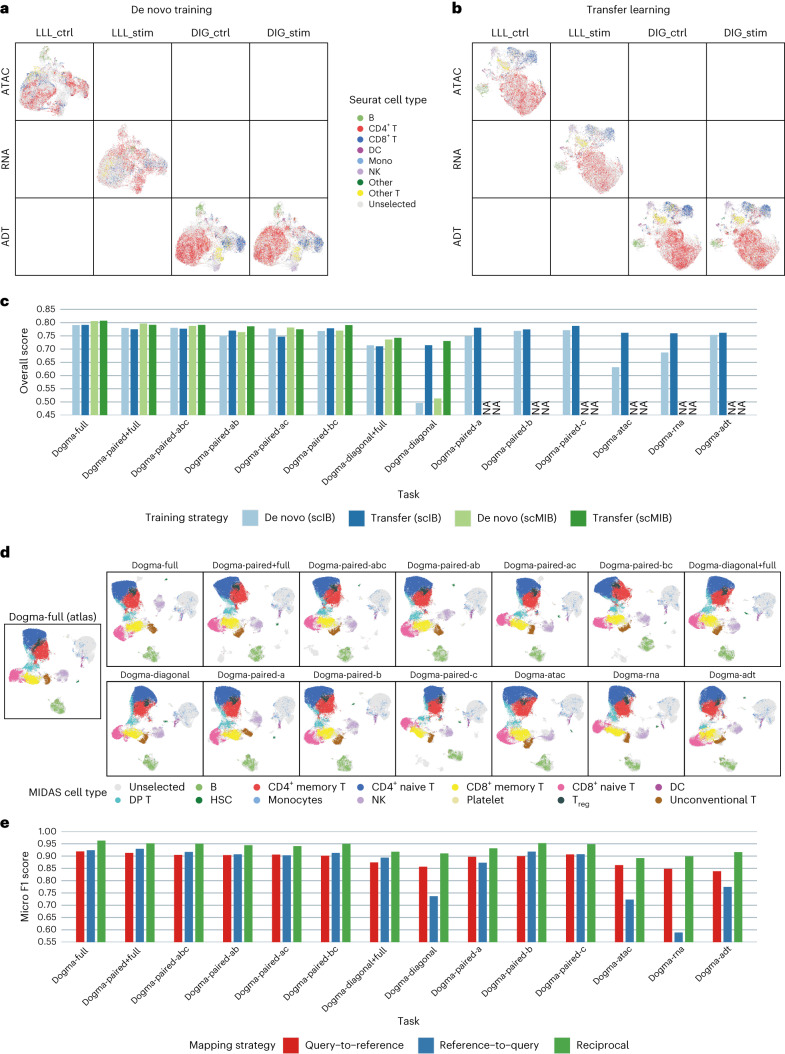
Qualitative and quantitative evaluation of MIDAS on knowledge transfer tasks. **a**,**b**, UMAP visualization of the biological states inferred by the de novo trained (**a**) and transfer-learned (**b**) MIDAS on the dogma-diagonal dataset. **c**, Overall scIB and scMIB scores reflecting transfer-learned and de novo trained MIDAS performance on 14 dogma mosaic integration tasks; NA, not available. **d**, UMAP visualization of the biological states obtained by reciprocal reference mapping with 14 dogma mosaic datasets (columns 2–8). Column 1 shows the dogma-full atlas integration. **e**, Label transfer micro F1 scores representing performance on 14 dogma mosaic query datasets.

We assessed the performance of MIDAS in the model transfer scenario. For the transfer-learned models, we used UMAP to visualize the inferred biological states and technical noises and scMIB and scIB for integration benchmarking and compared the results of different tasks with those generated by de novo trained models. Transfer learning greatly improved performance on the dogma-diagonal, dogma-atac, dogma-rna and dogma-paired-a tasks, with performance levels on the other tasks maintained (Fig. 5a–c and Supplementary Figs. 22 and 23). For example, the de novo trained model failed to integrate well in the dogma-diagonal task due to lack of cell-to-cell correspondence across modalities (Fig. 5a), whereas the transfer-learned model with atlas knowledge successfully aligned the biological states across batches and modalities and formed groups consistent with cell types (Fig. 5b). The results obtained by transfer-learned models with all 14 datasets were not only comparable (Supplementary Fig. 23a,b) but also superior to those of many other methods that use the complete dataset (Fig. 5c and Supplementary Fig. 23b).

To assess the performance of MIDAS in the label transfer scenario, we compared the widely used query-to-reference mapping^50,51^, reference-to-query mapping^14,52^ and our proposed reciprocal reference mapping (Methods). For each strategy, we aligned each query dataset to the reference dataset and transferred cell-type labels through the k-nearest neighbors (kNN) algorithm, where the ground truth cell-type labels were taken from the trimodal PBMC atlas annotated by MIDAS. Visualization of the mapped biological states showed that reciprocal reference mapping with different query datasets yielded consistent results, with strong agreement with the atlas integration results obtained with the dogma-full dataset (Fig. 5d and Supplementary Fig. 24). Micro F1 scores indicated that reciprocal reference mapping outperformed the query-to-reference and reference-to-query mapping strategies for various forms of query data, achieving robust and accurate label transfer and thereby avoiding the need for de novo integration and downstream analysis (Fig. 5e).

Thus, MIDAS can be used to transfer atlas-level knowledge to various forms of users’ datasets without expensive de novo training or complex downstream analysis.

### Application of MIDAS on BMMC mosaic data

To investigate the application of MIDAS in single-cell datasets with continuous cell state changes, we constructed a human BMMC mosaic dataset, denoted ‘bm’, by combining three distinct batches (ICA, ASAP and CITE) obtained from publicly available scRNA-seq, ASAP-seq and CITE-seq datasets, respectively (Methods). The results of de novo integration on bm showed that MIDAS accurately aligned different modalities and removed batch effects while preserving cell-type information (Supplementary Fig. 25a). Through comparison, we found that MIDAS outperformed the other trimodal mosaic integration methods in both qualitative (Supplementary Fig. 25b) and quantitative (Supplementary Fig. 25c) results.

**Fig. 6:**
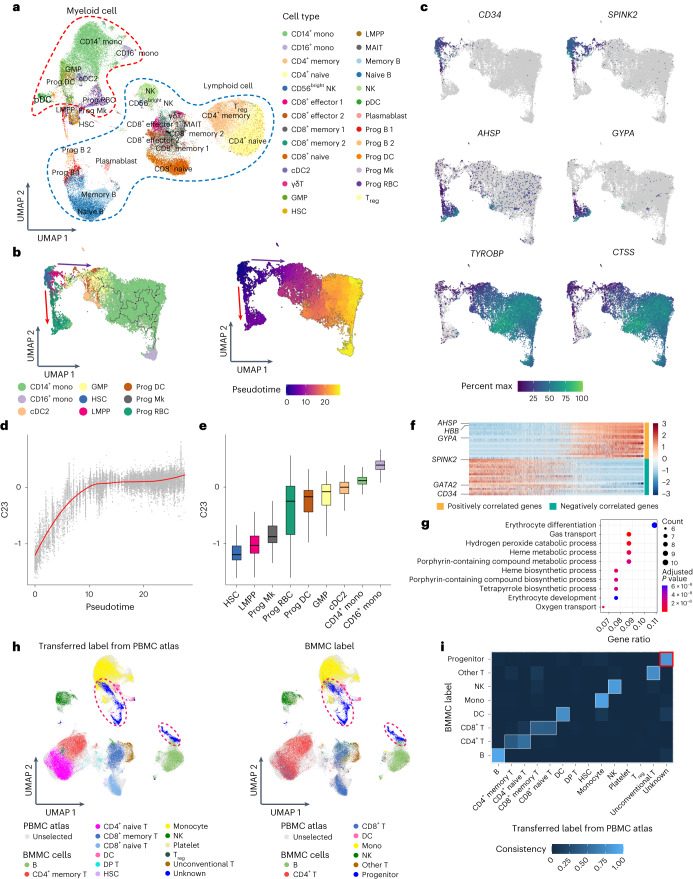
Application of MIDAS on BMMC mosaic dataset. **a**, UMAP visualization of the BMMC dataset labeled by Seurat; GMP, granulocyte–monocyte progenitor; LMPP, lymphoid-primed multipotential progenitor; MAIT, mucosal-associated invariant T cell; pDC, plasmacytoid DC; Mk, megakaryocyte; RBC, red blood cell; cDC2, type 2 conventional DC. **b**, UMAP visualization of the inferred trajectory and pseudotime based on 32-dimensional biological state latent representation in myeloid cells. **c**, UMAP visualization colored by imputed gene expression of key cell-type markers. **d**, Loess smoothed curve showing trends of C23 along with the pseudotime. **e**, Box plots showing C23 values of each cell type in **b** sorted by the medians (*n* = 19,405). In the box plots, the center lines indicate the median, boxes indicate the interquartile range, and whiskers indicate 1.5× interquartile range. **f**, Heat map showing scaled expression of the top 20 positively and negatively correlated genes of C23. **g**, Dot plot showing the top 10 significantly enriched Gene Ontology biological process terms of the positively correlated genes of C23 with clusterProfiler. Data were analyzed using a one-sided Fisher’s exact test, and *P* values were adjusted using the Benjamini–Hochberg method. **h**, UMAP visualization of the biological states obtained by cross-tissue reciprocal reference mapping between the PBMC atlas reference and the BMMC query dataset. The BMMC cells are colored by cell types transferred from the PBMC atlas using MIDAS (left) and by cell types annotated with Seurat (right). **i**, Confusion plot showing the label transfer consistency in a cross-tissue label transfer task on the BMMC mosaic dataset.

Next, we performed a pseudotime analysis of myeloid cells based on the 32-dimensional latent variables generated by MIDAS (Fig. 6a,b). The results showed that HSCs (marked by *CD34* and *SPINK2*) mainly differentiate into two branches. One branch corresponds to the precursor of megakaryocytes and erythrocytes (marked by *GYPA* and *AHSP*), and the other branch differentiates into granulocyte–macrophage progenitors through lymphoid-primed multipotential progenitors and finally differentiates into monocytes and DCs (marked by *TYROBP* and *CTSS*; Fig. 6c). This differentiation trajectory is consistent with the well-known developmental pathways of myeloid cells in bone marrow^53^, demonstrating that MIDAS’s 32-dimensional biological state latent variables can be applied to trajectory inference of cell differentiation. It is worth noting that the original data cannot be directly used for pseudotime analysis because one batch lacks RNA modality.

We further explored the possible biological meanings of each dimension in latent space. Notably, latent dimension 23 (C23) corresponded to the pseudotime inferred by Monocle 3 (ref. ^54^) and captured a gradual transition from HSCs to progenitor cells and finally to mature cells (Fig. 6d,e). These results suggest that C23 summarized a gene program contributing to cell development and differentiation. We further calculated correlations between C23 and all genes within the megakaryocyte and erythrocyte developing branch. The negatively correlated genes included canonical HSC markers, such as *SPINK2*, *CD34* and *GATA2* (Fig. 6f). Positively correlated genes included many erythrocyte-related genes, such as *AHSP*, *HBB* and *GYPA*, that are demonstrated to be involved in erythrocyte differentiation and function by clusterProfiler^55^ (Fig. 6g). These results showcase the ability of MIDAS biological state latent variables to capture meaningful biological information.

To showcase the capability of MIDAS in cross-tissue knowledge transfer, we used our constructed PBMC atlas as the reference dataset and bm as the query dataset (Supplementary Table 9). First, we performed experiments on model transfer and found that it yielded comparable qualitative and quantitative performance to de novo integration (Supplementary Fig. 26) while taking less than half the time (model transfer: 1.28 h; de novo integration: 2.61 h). Subsequently, we conducted experiments on label transfer, which showed that MIDAS successfully transferred cell-type labels from the PBMC atlas reference to the bm query dataset (Fig. 6h). MIDAS accurately identified an unknown cell type in the query dataset, which turned out to be the progenitor cell not present in the reference dataset (Fig. 6h,i).

## Discussion

By modeling the single-cell mosaic data generative process, MIDAS can precisely disentangle biological states and technical noises from the input and robustly align modalities to support multisource and heterogeneous integration analysis. MIDAS provides accurate and robust results and outperforms other methods when performing various mosaic integration tasks. It also integrates large datasets, as demonstrated with the atlas-scale integration of publicly available PBMC multiomics datasets. Moreover, MIDAS efficiently and flexibly transfers knowledge from reference to query datasets, enabling convenient handling of new multiomics data. With superior performance in dimensionality reduction and batch correction, MIDAS supports accurate downstream biological analysis. In addition to enabling clustering and cell-type identification for mosaic data, MIDAS can also assist in pseudotime analysis for cells with continuous states, which will be especially helpful when no RNA omics data are available. When transferring knowledge between different tissues, MIDAS is capable of aligning heterogeneous datasets and identifying cell types and even new types.

Recently, several methods for single-cell multimodal integration and knowledge transfer have been proposed (refer to Supplementary Note 2 for a detailed discussion). However, MIDAS supports simultaneous dimensionality reduction, modality complementing and batch correction in single-cell trimodal mosaic integration. MIDAS accurately integrates mosaic data with missing modalities, achieving results comparable to rectangular integration and superior to those obtained from other methods. These distinct advantages of MIDAS derive from the deep generative modeling, product of experts, information-theoretic disentanglement and self-supervised modality alignment components of the algorithm, which are specifically designed and inherently competent for the heterogeneous integration of data with missing features and modalities. In addition, MIDAS allows knowledge transfer across mosaic data modalities, batches and even tissues in a highly flexible and reliable manner, enabling researchers to conquer the vast bodies of data produced with continuously emerging multiomics techniques.

We envision two major developmental directions for MIDAS. At present, MIDAS integrates only three modalities. By fine-tuning the model architecture, we can achieve the integration of four or more modalities, overcoming the limitations of existing scMulti-omics sequencing technologies. In addition, the continuous incorporation of rapidly increasing bodies of newly generated scMulti-omics data is needed to update the model and improve the quality of the atlas. This process requires frequent model retraining, which is computationally expensive and time consuming. Thus, using incremental learning^56^ is an inevitable trend to achieve continuous mosaic integration without model retraining.

## Methods

### Deep generative modeling of mosaic single-cell multimodal data

For cell $n\in {{{\mathcal{N}}}}=\{1,\ldots ,N\}$ with batch ID ${s}_{n}\in {{{\mathcal{B}}}}=\{1,\ldots ,B\}$, let ${{{{\boldsymbol{x}}}}}_{n}^{m}\in {{\mathbb{N}}}^{{D}_{n}^{m}}$ be the count vector of size ${D}_{n}^{m}$ from modality *m* and ${{{{\boldsymbol{x}}}}}_{n}={\{{{{{\boldsymbol{x}}}}}_{n}^{m}\}}_{m\in {{{{\mathcal{M}}}}}_{n}}$ be the set of count vectors from the measured modalities ${{{{\mathcal{M}}}}}_{n}\subseteq {{{\mathcal{M}}}}=\{{{{\rm{ATAC}}}},{{{\rm{RNA}}}},{{{\rm{ADT}}}}\}$. We define two modality-agnostic low-dimensional latent variables ${{{\boldsymbol{c}}}}\in {{\mathbb{R}}}^{{D}^{c}}$ and ${{{\boldsymbol{u}}}}\in {{\mathbb{R}}}^{{D}^{u}}$ to represent each cell’s biological state and technical noise, respectively. To model the generative process of the observed variables ***x*** and *s* for each cell, we factorize the joint distribution of all variables as

1 $$\begin{array}{ll}p({{{\boldsymbol{x}}}},s,{{{\boldsymbol{c}}}},{{{\boldsymbol{u}}}})&=p({{{\boldsymbol{c}}}})p({{{\boldsymbol{u}}}})p(s| {{{\boldsymbol{u}}}})p({{{\boldsymbol{x}}}}| {{{\boldsymbol{c}}}},{{{\boldsymbol{u}}}})\\ &=p({{{\boldsymbol{c}}}})p({{{\boldsymbol{u}}}})p(s| {{{\boldsymbol{u}}}})\mathop{\prod}\limits_{m\in {{{{\mathcal{M}}}}}_{n}}p({{{{\boldsymbol{x}}}}}^{m}| {{{\boldsymbol{c}}}},{{{\boldsymbol{u}}}})\end{array}$$

where we assume that ***c*** and ***u*** are independent of each other, the batch ID *s* only depends on ***u*** to facilitate the disentanglement of both latent variables, and the count variables ${\{{{{{\boldsymbol{x}}}}}^{m}\}}_{m\in {{{{\mathcal{M}}}}}_{n}}$ from different modalities are conditional independent given ***c*** and ***u***.

Based on the above factorization, we define a generative model for ***x*** and *s* as

2 $$p({{{\boldsymbol{c}}}})={{\mathrm{Normal}}}\,({{{\boldsymbol{c}}}}| {{{\boldsymbol{0}}}},{{{\boldsymbol{I}}}})$$

3 $$p({{{\boldsymbol{u}}}})={{\mathrm{Normal}}}\,({{{\boldsymbol{u}}}}| {{{\boldsymbol{0}}}},{{{\boldsymbol{I}}}})$$

4 $${{{\boldsymbol{\pi }}}}={g}^{s}({{{\boldsymbol{u}}}};{{{{\boldsymbol{\theta }}}}}^{s})$$

5 $${p}_{{{{\boldsymbol{\theta }}}}}(s| {{{\boldsymbol{u}}}})={{\mathrm{Categorical}}}\,(s| {{{\boldsymbol{\pi }}}})$$

6 $${{{{\boldsymbol{\lambda }}}}}^{m}={g}^{m}({{{\boldsymbol{c}}}},{{{\boldsymbol{u}}}};{{{{\boldsymbol{\theta }}}}}^{m})\,{{{\rm{for}}}}\,m\in {{{{\mathcal{M}}}}}_{n}$$

7 $${p}_{{{{\boldsymbol{\theta }}}}}({{{{\boldsymbol{x}}}}}^{m}| {{{\boldsymbol{c}}}},{{{\boldsymbol{u}}}})=\left\{\begin{array}{l}{{\mathrm{Bernoulli}}}\,({{{{\boldsymbol{x}}}}}^{m}| {{{{\boldsymbol{\lambda }}}}}^{m})\,{{{\rm{if}}}}\,m={{{\rm{ATAC}}}}\quad \\ {{\mathrm{Poisson}}}\,({{{{\boldsymbol{x}}}}}^{m}| {{{{\boldsymbol{\lambda }}}}}^{m})\,{{{\rm{if}}}}\,m\in \{{{{\rm{RNA}}}},{{{\rm{ADT}}}}\}\quad \end{array}\right.{{{\rm{for}}}}\,m\in {{{{\mathcal{M}}}}}_{n}$$

where the priors *p*(***c***) and *p*(***u***) are set as standard Gaussians. The likelihood *p*_***θ***_(*s*|***u***) is set as a categorical distribution with probability vector ***π*** ∈ Δ^*B*−1^ generated through a batch ID decoder *g*^*s*^, which is a neural network with learnable parameters ***θ***^*s*^. The likelihood *p*_***θ***_(***x***^*m*^|***c***, ***u***) is set as a Bernoulli distribution with mean ${{{{\boldsymbol{\lambda }}}}}^{m}\in {[0,1]}^{{D}_{n}^{m}}$ when *m* = ATAC and as a Poisson distribution with mean ${{{{\boldsymbol{\lambda }}}}}^{m}\in {{\mathbb{R}}}_{+}^{{D}_{n}^{m}}$ when *m* ∈ {RNA, ADT}, where ***λ***^*m*^ is generated through a modality decoder neural network *g*^*m*^ parameterized by ***θ***^*m*^. To mitigate overfitting and improve generalization, we share parameters of the first few layers of different modality decoders ${\{{g}^{m}\}}_{m\in {{{\mathcal{M}}}}}$ (the gray parts of the decoders in Fig. 1b, middle). The corresponding graphical model is shown in Fig. 1b (left).

Given the observed data ${\{{{{{\boldsymbol{x}}}}}_{n},{s}_{n}\}}_{n\in {{{\mathcal{N}}}}}$, we aim to fit the model parameters ${{{\boldsymbol{\theta }}}}=\{{{{{\boldsymbol{\theta }}}}}^{s},{\{{{{{\boldsymbol{\theta }}}}}^{m}\}}_{m\in {{{\mathcal{M}}}}}\}$ and meanwhile infer the posteriors of latent variables {***c***, ***u***} for each cell. This can be achieved by using the stochastic gradient variational Bayes^41^, which maximizes the expected evidence lower bound (ELBO) for individual data points. The ELBO for each individual data point {***x***_*n*_, *s*_*n*_} can be written as

8 $$\begin{array}{ll}{{\mathrm{ELBO}}}\,({{{\boldsymbol{\theta }}}},{{{\boldsymbol{\phi }}}};{{{{\boldsymbol{x}}}}}_{n},{s}_{n})\\ \triangleq {{\mathbb{E}}}_{\displaystyle{q}_{{{{\boldsymbol{\phi }}}}}\displaystyle({{{\boldsymbol{c}}}},{{{\boldsymbol{u}}}}| {{{{\boldsymbol{x}}}}}_{n},{s}_{n})}\left[\log \displaystyle\frac{{p}_{{{{\boldsymbol{\theta }}}}}({{{{\boldsymbol{x}}}}}_{n},{s}_{n},{{{\boldsymbol{c}}}},{{{\boldsymbol{u}}}})}{{q}_{{{{\boldsymbol{\phi }}}}}({{{\boldsymbol{c}}}},{{{\boldsymbol{u}}}}| {{{{\boldsymbol{x}}}}}_{n},{s}_{n})}\right]\\ =\displaystyle{{\mathbb{E}}}_{\displaystyle{q}_{{{{\boldsymbol{\phi }}}}}({{{\boldsymbol{c}}}},{{{\boldsymbol{u}}}}| {{{{\boldsymbol{x}}}}}_{n},{s}_{n})}\displaystyle\left[\log {p}_{{{{\boldsymbol{\theta }}}}}({{{{\boldsymbol{x}}}}}_{n},{s}_{n}| {{{\boldsymbol{c}}}},{{{\boldsymbol{u}}}})\right]-{{{\rm{KL}}}}\left[\displaystyle{q}_{{{{\boldsymbol{\phi }}}}}({{{\boldsymbol{c}}}},{{{\boldsymbol{u}}}}| {{{{\boldsymbol{x}}}}}_{n},{s}_{n})\parallel p({{{\boldsymbol{c}}}},{{{\boldsymbol{u}}}})\right]\\ =\displaystyle{{\mathbb{E}}}_{\displaystyle{q}_{{{{\boldsymbol{\phi }}}}}({{{\boldsymbol{c}}}},{{{\boldsymbol{u}}}}| {{{{\boldsymbol{x}}}}}_{n},{s}_{n})}\left[\log {p}_{{{{\boldsymbol{\theta }}}}}({s}_{n}| {{{\boldsymbol{u}}}})+\mathop{\sum}\limits_{m\in {{{{\mathcal{M}}}}}_{n}}\log {p}_{{{{\boldsymbol{\theta }}}}}({{{{\boldsymbol{x}}}}}_{n}^{m}| {{{\boldsymbol{c}}}},{{{\boldsymbol{u}}}})\right]\\ \quad-{{{\rm{KL}}}}\left[{q}_{{{{\boldsymbol{\phi }}}}}({{{\boldsymbol{c}}}},{{{\boldsymbol{u}}}}| {{{{\boldsymbol{x}}}}}_{n},{s}_{n})\parallel p({{{\boldsymbol{c}}}},{{{\boldsymbol{u}}}})\right]\end{array}$$

where *q*_***ϕ***_(***c***, ***u***|***x***_*n*_, *s*_*n*_), with learnable parameters ***ϕ***, is the variational approximation of the true posterior *p*(***c***, ***u***|***x***_*n*_, *s*_*n*_) and is typically implemented by neural networks, and KL( ⋅ ∥ ⋅ ) is the Kullback–Leibler divergence between two distributions.

### Scalable variational inference via the product of experts

Let $M=| {{{\mathcal{M}}}}|$ be the total modality number. Because there are (2^*M*^ − 1) possible modality combinations for the count data ${{{{\boldsymbol{x}}}}}_{n}={\{{{{{\boldsymbol{x}}}}}_{n}^{m}\}}_{m\in {{{{\mathcal{M}}}}}_{n}\subseteq {{{\mathcal{M}}}}}$, naively implementing *q*_***ϕ***_(***c***, ***u***|***x***_*n*_, *s*_*n*_) in Eq. (8) requires (2^*M*^ − 1) different neural networks to handle different cases of input (***x***_*n*_, *s*_*n*_), making inference unscalable. Let ***z*** = {***c***, ***u***}. Inspired by Wu and Goodman^57^, which uses the product of experts to implement variational inference in a combinatorial way, we factorize the posterior *p*(***z***|***x***_*n*_, *s*_*n*_) and define its variational approximation *q*_***ϕ***_(***z***|***x***_*n*_, *s*_*n*_) as follows:

9 $$\begin{array}{ll}\displaystyle p({{{\boldsymbol{z}}}}| {{{{\boldsymbol{x}}}}}_{n},{s}_{n})&= \displaystyle \frac{p({s}_{n})}{p({{{{\boldsymbol{x}}}}}_{n},{s}_{n})}\left(\mathop{\prod}\limits_{m\in {{{{\mathcal{M}}}}}_{n}}p({{{{\boldsymbol{x}}}}}_{n}^{m})\right)p({{{\boldsymbol{z}}}})\frac{p({{{\boldsymbol{z}}}}| {s}_{n})}{p({{{\boldsymbol{z}}}})}\mathop{\prod}\limits_{m\in {{{{\mathcal{M}}}}}_{n}}\frac{p({{{\boldsymbol{z}}}}| {{{{\boldsymbol{x}}}}}_{n}^{m})}{p({{{\boldsymbol{z}}}})}\\ &\approx \displaystyle\frac{p({s}_{n})}{p({{{{\boldsymbol{x}}}}}_{n},{s}_{n})}\left(\mathop{\prod}\limits_{m\in {{{{\mathcal{M}}}}}_{n}}p({{{{\boldsymbol{x}}}}}_{n}^{m})\right)p({{{\boldsymbol{z}}}})\frac{{q}_{{{{\boldsymbol{\phi }}}}}({{{\boldsymbol{z}}}}| {s}_{n})}{p({{{\boldsymbol{z}}}})}\mathop{\prod}\limits_{m\in {{{{\mathcal{M}}}}}_{n}}\frac{{q}_{{{{\boldsymbol{\phi }}}}}({{{\boldsymbol{z}}}}| {{{{\boldsymbol{x}}}}}_{n}^{m})}{p({{{\boldsymbol{z}}}})}\\ &\triangleq \, \displaystyle {q}_{{{{\boldsymbol{\phi }}}}}({{{\boldsymbol{z}}}}| {{{{\boldsymbol{x}}}}}_{n},{s}_{n})\end{array}$$

where *q*_***ϕ***_(***z***|*s*_*n*_) and ${q}_{{{{\boldsymbol{\phi }}}}}({{{\boldsymbol{z}}}}| {{{{\boldsymbol{x}}}}}_{n}^{m})$ are the variational approximations of the true posteriors *p*(***z***|*s*_*n*_) and $p({{{\boldsymbol{z}}}}| {{{{\boldsymbol{x}}}}}_{n}^{m})$, respectively (see Supplementary Note 3 for detailed derivation). Let ${\widetilde{q}}_{{{{\boldsymbol{\phi }}}}}({{{\boldsymbol{z}}}}| {s}_{n})=\frac{1}{{C}^{s}}\frac{{q}_{{{{\boldsymbol{\phi }}}}}({{{\boldsymbol{z}}}}| {s}_{n})}{p({{{\boldsymbol{z}}}})}$ and ${\widetilde{q}}_{{{{\boldsymbol{\phi }}}}}({{{\boldsymbol{z}}}}| {{{{\boldsymbol{x}}}}}_{n}^{m})=\frac{1}{{C}^{m}}\frac{{q}_{{{{\boldsymbol{\phi }}}}}({{{\boldsymbol{z}}}}| {{{{\boldsymbol{x}}}}}_{n}^{m})}{p({{{\boldsymbol{z}}}})}$ be the normalized quotients of distributions with normalizing constants *C*^*s*^ and *C*^*m*^, respectively. From Eq. (9), we further acquire

10 $$\begin{array}{ll}\displaystyle{q}_{{{{\boldsymbol{\phi }}}}}({{{\boldsymbol{z}}}}| {{{{\boldsymbol{x}}}}}_{n},{s}_{n})& \displaystyle \propto p({{{\boldsymbol{z}}}})\frac{{q}_{{{{\boldsymbol{\phi }}}}}({{{\boldsymbol{z}}}}| {s}_{n})}{p({{{\boldsymbol{z}}}})}\mathop{\prod}\limits_{m\in {{{{\mathcal{M}}}}}_{n}}\frac{{q}_{{{{\boldsymbol{\phi }}}}}({{{\boldsymbol{z}}}}| {{{{\boldsymbol{x}}}}}_{n}^{m})}{p({{{\boldsymbol{z}}}})}\\ &= \displaystyle p({{{\boldsymbol{z}}}}){C}^{s}{\widetilde{q}}_{{{{\boldsymbol{\phi }}}}}({{{\boldsymbol{z}}}}| {s}_{n})\mathop{\prod}\limits_{m\in {{{{\mathcal{M}}}}}_{n}}{C}^{m}{\widetilde{q}}_{{{{\boldsymbol{\phi }}}}}({{{\boldsymbol{z}}}}| {{{{\boldsymbol{x}}}}}_{n}^{m})\\ &\propto \displaystyle p({{{\boldsymbol{z}}}}){\widetilde{q}}_{{{{\boldsymbol{\phi }}}}}({{{\boldsymbol{z}}}}| {s}_{n})\mathop{\prod}\limits_{m\in {{{{\mathcal{M}}}}}_{n}}{\widetilde{q}}_{{{{\boldsymbol{\phi }}}}}({{{\boldsymbol{z}}}}| {{{{\boldsymbol{x}}}}}_{n}^{m})\end{array}$$

where we set *q*_***ϕ***_(***z***|*s*_*n*_) and ${q}_{{{{\boldsymbol{\phi }}}}}({{{\boldsymbol{z}}}}| {{{{\boldsymbol{x}}}}}_{n}^{m})$ to be diagonal Gaussians, resulting in ${\widetilde{q}}_{{{{\boldsymbol{\phi }}}}}({{{\boldsymbol{z}}}}| {s}_{n})$ and ${\widetilde{q}}_{{{{\boldsymbol{\phi }}}}}({{{\boldsymbol{z}}}}| {{{{\boldsymbol{x}}}}}_{n}^{m})$ being diagonal Gaussians, which are defined as

11 $$\displaystyle \left({{{{\boldsymbol{\mu }}}}}_{n}^{s},{{{{\boldsymbol{\nu }}}}}_{n}^{s}\right)={\displaystyle{f}^{\,s}({s}_{n};{{{{\boldsymbol{\phi }}}}}^{s})}$$

12 $$\displaystyle {\widetilde{q}}_{{{{\boldsymbol{\phi }}}}}({{{\boldsymbol{z}}}}| {s}_{n})= \displaystyle {{\mathrm{Normal}}}\, {\displaystyle \left[{{{\boldsymbol{z}}}}| {{{{\boldsymbol{\mu }}}}}_{n}^{s},{{\mathrm{diag}}}\,\left({{{{\boldsymbol{\nu }}}}}_{n}^{s}\right)\right]}$$

13 $${\displaystyle \left({{{{\boldsymbol{\mu }}}}}_{n}^{m},{{{{\boldsymbol{\nu }}}}}_{n}^{m}\right)={f}^{m}({{{{\boldsymbol{x}}}}}_{n}^{m};{{{{\boldsymbol{\phi }}}}}^{m})\,{{{\rm{for}}}}\,m\in {{{{\mathcal{M}}}}}_{n}}$$

14 $$\displaystyle {\widetilde{q}}_{{{{\boldsymbol{\phi }}}}}({{{\boldsymbol{z}}}}| {{{{\boldsymbol{x}}}}}_{n}^{m})= \displaystyle{{\mathrm{Normal}}}\,\left[{{{\boldsymbol{z}}}}| {{{{\boldsymbol{\mu }}}}}_{n}^{m},{{\mathrm{diag}}}\,\left({{{{\boldsymbol{\nu }}}}}_{n}^{m}\right)\right]{{{\rm{for}}}}\,m\in {{{{\mathcal{M}}}}}_{n}$$

where *f*^*s*^, with parameters ***ϕ***^*s*^, is the batch ID encoder neural network for generating the mean ${{{{\boldsymbol{\mu }}}}}_{n}^{s}$ and variance ${{{{\boldsymbol{\nu }}}}}_{n}^{s}$ of ${\widetilde{q}}_{{{{\boldsymbol{\phi }}}}}({{{\boldsymbol{z}}}}| {s}_{n})$, and *f*^*m*^, with parameters ***ϕ***^*m*^, is the modality encoder neural network for generating the mean ${{{{\boldsymbol{\mu }}}}}_{n}^{m}$ and variance ${{{{\boldsymbol{\nu }}}}}_{n}^{m}$ of ${\widetilde{q}}_{{{{\boldsymbol{\phi }}}}}({{{\boldsymbol{z}}}}| {{{{\boldsymbol{x}}}}}_{n}^{m})$. The operator diag( ⋅ ) converts a vector into a diagonal matrix.

In Eq. (10), because *q*_***ϕ***_(***z***|***x***_*n*_, *s*_*n*_) is proportional to the product of individual Gaussians (or ‘experts’), itself is a Gaussian whose mean ***μ***_*n*_ and variance ***ν***_*n*_ can be calculated using those of the individual Gaussians:

15 $$\begin{array}{ll}{{{{\boldsymbol{\mu }}}}}_{n}&=\left(\displaystyle\frac{{{{{\boldsymbol{\mu }}}}}_{n}^{s}}{{{{{\boldsymbol{\nu }}}}}_{n}^{s}}+\mathop{\sum}\limits_{m\in {{{{\mathcal{M}}}}}_{n}}\frac{{{{{\boldsymbol{\mu }}}}}_{n}^{m}}{{{{{\boldsymbol{\nu }}}}}_{n}^{m}}\right)\odot {{{{\boldsymbol{\nu }}}}}_{n}\\ {{{{\boldsymbol{\nu }}}}}_{n}&={\left(1+ \displaystyle\frac{1}{{{{{\boldsymbol{\nu }}}}}_{n}^{s}}+\mathop{\sum}\limits_{m\in {{{{\mathcal{M}}}}}_{n}}\frac{1}{{{{{\boldsymbol{\nu }}}}}_{n}^{m}}\right)}^{-1}\end{array}$$

where ⊙ is the Hadamard product.

In doing this, *q*_***ϕ***_(***z***|***x***_*n*_, *s*_*n*_) is modularized into (*M* + 1) neural networks to handle (2^*M*^ − 1) different modality combinations, increasing the model’s scalability. Similar to the modality decoders, we also share parameters of the last few layers of different modality encoders ${\{{f}^{{\,}m}\}}_{m\in {{{\mathcal{M}}}}}$ (the gray parts of the encoders in Fig. 1b, middle) to improve generalization.

### Handling missing features via padding and masking

For each modality, as different cells can have different feature sets (for example, genes for RNA modality), it is hard to use a fixed-size neural network to handle these cells. To remedy this, we first convert ${{{{\boldsymbol{x}}}}}_{n}^{m}$ of variable size into a fixed-size vector for inference. For modality *m*, let ${{{{\mathcal{F}}}}}_{n}^{m}$ be the features of cell *n* and let ${{{{\mathcal{F}}}}}^{m}={\bigcup }_{n\in {{{\mathcal{N}}}}}{{{{\mathcal{F}}}}}_{n}^{m}$ be the feature union of all cells. The missing features of cell *n* can then be defined as ${\overline{{{{\mathcal{F}}}}}}_{n}^{m}={{{{\mathcal{F}}}}}^{m}\setminus {{{{\mathcal{F}}}}}_{n}^{m}$. We pad ${{{{\boldsymbol{x}}}}}_{n}^{m}$ of size ${D}_{n}^{m}$ with zeros corresponding to its missing features ${\overline{{{{\mathcal{F}}}}}}_{n}^{m}$ through a zero-padding function *h*,

16 $$\begin{array}{ll}{\widetilde{{{{\boldsymbol{x}}}}}}_{n}^{m}&=h({{{{\boldsymbol{x}}}}}_{n}^{m})\end{array}$$

where ${\widetilde{{{{\boldsymbol{x}}}}}}_{n}^{m}$ is the zero-padded count vector of constant size ${D}^{m}=| {{{{\mathcal{F}}}}}^{m}|$. The modality encoding process is thus decomposed as

17 $$\begin{array}{rcl}\left({{{{\boldsymbol{\mu }}}}}_{n}^{m},{{{{\boldsymbol{\nu }}}}}_{n}^{m}\right)&=& {{f}}^{m}({{{\boldsymbol{x}}}}_{n}^{m}{;}{{{{\boldsymbol{\phi }}}}}^{m})\\ &=&{\widehat{{f}}}^{m}\left({h}({{{{\boldsymbol{x}}}}}_{n}^{m}){;}{{{{\boldsymbol{\phi }}}}}^{m}\right)\\ &=&{\widehat{{f}}}^{m}\left({\widetilde{{{{\boldsymbol{x}}}}}}_{n}^{m}{;}{{{{\boldsymbol{\phi }}}}}^{m}\right)\end{array}$$

where ${\widehat{f}}^{{\,}m}$ is the latter part of the modality encoder to handle a fixed-size input ${\widetilde{{{{\boldsymbol{x}}}}}}_{n}^{m}$. However, given the sampled latent variables {***c***_*n*_, ***u***_*n*_}, to calculate the likelihood ${p}_{{{{\boldsymbol{\theta }}}}}({{{{\boldsymbol{x}}}}}_{n}^{m}| {{{{\boldsymbol{c}}}}}_{n},{{{{\boldsymbol{u}}}}}_{n})$, we also need to generate a mean ${{{{\boldsymbol{\lambda }}}}}_{n}^{m}$ of variable size for ${{{{\boldsymbol{x}}}}}_{n}^{m}$. To achieve this, we decompose the modality decoding process as

18 $$\begin{array}{ll}{{{{\boldsymbol{\lambda }}}}}_{n}^{m}&={g}^{m}({{{{\boldsymbol{c}}}}}_{n},{{{{\boldsymbol{u}}}}}_{n};{{{{\boldsymbol{\theta }}}}}^{m})\\ &={h}^{-1}\left[{\widehat{g}}^{m}({{{{\boldsymbol{c}}}}}_{n},{{{{\boldsymbol{u}}}}}_{n};{{{{\boldsymbol{\theta }}}}}^{m})\right]\\ &={h}^{-1}\left({\widetilde{{{{\boldsymbol{\lambda }}}}}}_{n}^{m}\right)\end{array}$$

where ${\widehat{g}}^{m}$ is the front part of the modality decoder to generate the mean ${\widetilde{{{{\boldsymbol{\lambda }}}}}}_{n}^{m}$ of fixed-size *D*^*m*^, and *h*^−1^ (the inverse function of *h*) is the masking function to remove the padded missing features ${\overline{{{{\mathcal{F}}}}}}_{n}^{m}$ from ${\widetilde{{{{\boldsymbol{\lambda }}}}}}_{n}^{m}$ to generate ${{{{\boldsymbol{\lambda }}}}}_{n}^{m}$. Note that ${\widetilde{{{{\boldsymbol{\lambda }}}}}}_{n}^{m}$ can also be taken as the imputed values for downstream analyses (see ‘Imputation for missing modalities and features’ and ‘Batch correction via latent variable manipulation’).

### Self-supervised modality alignment

To achieve cross-modal inference in downstream tasks, we resort to aligning different modalities in the latent space. Leveraging self-supervised learning, we first use each cell’s multimodal observation $\{{\{{{{{\boldsymbol{x}}}}}_{n}^{m}\}}_{m\in {{{{\mathcal{M}}}}}_{n}},{s}_{n}\}$ to construct unimodal observations ${\{{{{{\boldsymbol{x}}}}}_{n}^{m},{s}_{n}\}}_{m\in {{{{\mathcal{M}}}}}_{n}}$, each of which is associated with the latent variables ***z***^*m*^ = {***c***^*m*^, ***u***^*m*^}. We then construct a pretext task, which enforces modality alignment by regularizing on the joint space of unimodal variational posteriors with the dispersion of latent variables as a penalty (Fig. 1b, top right), corresponding to a modality alignment loss

19 $$\begin{array}{rcl}\displaystyle{l}^{{{{\rm{mod}}}}}({{{\boldsymbol{\phi }}}};{{{{\boldsymbol{x}}}}}_{n},{s}_{n})&\triangleq & \alpha \int\,v(\widetilde{{{{\boldsymbol{z}}}}}){q}_{{{{\boldsymbol{\phi }}}}}(\widetilde{{{{\boldsymbol{z}}}}}| {{{{\boldsymbol{x}}}}}_{n},{s}_{n})d\widetilde{{{{\boldsymbol{z}}}}}\\ \displaystyle &=&\alpha \,{{\mathbb{E}}}_{{q}_{{{{\boldsymbol{\phi }}}}}(\widetilde{{{{\boldsymbol{z}}}}}| {{{{\boldsymbol{x}}}}}_{n},{s}_{n})}v(\widetilde{{{{\boldsymbol{z}}}}})\end{array}$$

where *α* > 0 is the loss weight, $\widetilde{{{{\boldsymbol{z}}}}}={\{{{{{\boldsymbol{z}}}}}^{m}\}}_{m\in {{{{\mathcal{M}}}}}_{n}}$ is the set of latent variables, and ${q}_{{{{\boldsymbol{\phi }}}}}(\widetilde{{{{\boldsymbol{z}}}}}| {{{{\boldsymbol{x}}}}}_{n},{s}_{n})$ represents the joint distribution of unimodal variational posteriors because

20 $$\begin{array}{ll}{q}_{{{{\boldsymbol{\phi }}}}}(\widetilde{{{{\boldsymbol{z}}}}}| {{{{\boldsymbol{x}}}}}_{n},{s}_{n})&={q}_{{{{\boldsymbol{\phi }}}}}({\{{{{{\boldsymbol{z}}}}}^{m}\}}_{m\in {{{{\mathcal{M}}}}}_{n}}| {{{{\boldsymbol{x}}}}}_{n},{s}_{n})\\ &=\mathop{\prod}\limits_{m\in {{{{\mathcal{M}}}}}_{n}}{q}_{{{{\boldsymbol{\phi }}}}}({{{{\boldsymbol{z}}}}}^{m}| {{{{\boldsymbol{x}}}}}_{n},{s}_{n})\\ &=\mathop{\prod}\limits_{m\in {{{{\mathcal{M}}}}}_{n}}{q}_{{{{\boldsymbol{\phi }}}}}({{{{\boldsymbol{z}}}}}^{m}| {{{{\boldsymbol{x}}}}}_{n}^{m},{s}_{n})\end{array}$$

In Eq. (19), $v(\widetilde{{{{\boldsymbol{z}}}}})$ is the sum of squared deviations, which measures the dispersion among different elements in $\widetilde{{{{\boldsymbol{z}}}}}$ and is used to regularize ${q}_{{{{\boldsymbol{\phi }}}}}(\widetilde{{{{\boldsymbol{z}}}}}| {{{{\boldsymbol{x}}}}}_{n},{s}_{n})$; it is defined as

21 $$\begin{array}{ll}v(\widetilde{{{{\boldsymbol{z}}}}})&\triangleq \mathop{\sum}\limits_{m\in {{{{\mathcal{M}}}}}_{n}}\parallel {{{{\boldsymbol{z}}}}}^{m}-\bar{{{{\boldsymbol{z}}}}}{\parallel }_{2}^{2}\end{array}$$

where $\bar{{{{\boldsymbol{z}}}}}=\frac{1}{| {{{{\mathcal{M}}}}}_{n}| }{\sum }_{m\in {{{{\mathcal{M}}}}}_{n}}{{{{\boldsymbol{z}}}}}^{m}$ is the mean, and ∥ ⋅ ∥_2_ is the Euclidean distance.

Note that the computation of ${q}_{{{{\boldsymbol{\phi }}}}}({{{{\boldsymbol{z}}}}}^{m}| {{{{\boldsymbol{x}}}}}_{n}^{m},{s}_{n})$ in Eq. (20) is efficient. Because ${q}_{{{{\boldsymbol{\phi }}}}}({{{{\boldsymbol{z}}}}}^{m}| {{{{\boldsymbol{x}}}}}_{n}^{m},{s}_{n})={q}_{{{{\boldsymbol{\phi }}}}}({{{\boldsymbol{z}}}}| {{{{\boldsymbol{x}}}}}_{n}^{m},{s}_{n}){| }_{{{{\boldsymbol{z}}}} = {{{{\boldsymbol{z}}}}}^{m}}$, according to Eq. (10), we have

22 $$\begin{array}{ll}{q}_{{{{\boldsymbol{\phi }}}}}({{{\boldsymbol{z}}}}| {{{{\boldsymbol{x}}}}}_{n}^{m},{s}_{n})&\propto p({{{\boldsymbol{z}}}}){\widetilde{q}}_{{{{\boldsymbol{\phi }}}}}({{{\boldsymbol{z}}}}| {s}_{n}){\widetilde{q}}_{{{{\boldsymbol{\phi }}}}}({{{\boldsymbol{z}}}}| {{{{\boldsymbol{x}}}}}_{n}^{m})\\ \end{array}$$

As the mean and covariance of each Gaussian term on the righthand side of Eq. (22) was already obtained when inferring *q*_***ϕ***_(***z***|***x***_*n*_, *s*_*n*_) (Eq. (10)), the mean and covariance of ${q}_{{{{\boldsymbol{\phi }}}}}({{{\boldsymbol{z}}}}| {{{{\boldsymbol{x}}}}}_{n}^{m},{s}_{n})$ can be directly calculated using Eq. (15), avoiding the need of passing each constructed unimodal observation to the encoders.

### Information-theoretic disentanglement of latent variables

To better disentangle the biological state ***c*** and the technical noise ***u***, we adopt an information-theoretic approach, the information bottleneck (IB)^34^, to control information flow during inference. We define two types of IB, where the technical IB prevents batch-specific information being encoded into ***c*** by minimizing the mutual information (MI) between *s* and ***c***, and the biological IB prevents biological information being encoded into ***u*** by minimizing the MI between ***x*** and ***u*** (Fig. 1b, bottom right). Let I( ⋅ , ⋅ ) denote the MI between two variables. We implement both IBs by minimizing the weighted sum of I(*s*, ***c***) and I(***x***, ***u***),

23 $$\begin{array}{ll}\displaystyle{\beta }^{s}{{{\rm{I}}}}(s,{{{\boldsymbol{c}}}})+{\beta }^{x}{{{\rm{I}}}}({{{\boldsymbol{x}}}},{{{\boldsymbol{u}}}})=\displaystyle{\beta }^{s}{{\mathbb{E}}}_{\displaystyle p(s,{{{\boldsymbol{c}}}})}\left[\log \frac{p(s,{{{\boldsymbol{c}}}})}{p(s)p({{{\boldsymbol{c}}}})}\right]+{\beta }^{x}{{\mathbb{E}}}_{\displaystyle p({{{\boldsymbol{x}}}},{{{\boldsymbol{u}}}})}\left[\log \frac{p({{{\boldsymbol{x}}}},{{{\boldsymbol{u}}}})}{p({{{\boldsymbol{x}}}})p({{{\boldsymbol{u}}}})}\right]\\ \approx \frac{1}{N}\mathop{\sum}\limits_{n}\left[{\beta }^{s}{{\mathbb{E}}}_{\displaystyle{q}_{{{{\boldsymbol{\phi }}}}}({{{\boldsymbol{c}}}}| {{{{\boldsymbol{x}}}}}_{{n}},{\displaystyle{s}}_{{n}})}\left[\log {p}_{\widehat{{{{\boldsymbol{\eta }}}}}}({s}_{n}| {{{\boldsymbol{c}}}})\right]+{\beta }^{x}{{{\rm{KL}}}}\left[{q}_{{{{\boldsymbol{\phi }}}}}({{{\boldsymbol{u}}}}| {{{{\boldsymbol{x}}}}}_{n}, {\displaystyle s}_{n})\parallel p({{{\boldsymbol{u}}}})\right]\right.\\ \left.-{\beta }^{x}{{\mathbb{E}}}_{\displaystyle{q}_{{{{\boldsymbol{\phi }}}}}({{{\boldsymbol{u}}}}| {{{{\boldsymbol{x}}}}}_{n},{\displaystyle{s}}_{n})}\left[\log {p}_{{{{\boldsymbol{\theta }}}}}({s}_{n}| {{{\boldsymbol{u}}}})\right]\right]+{{{\rm{const.}}}}\end{array}$$

where *β*^*s*^, *β*^*x*^ > 0 are the weights, and ${p}_{\widehat{{{{\boldsymbol{\eta }}}}}}(s| {{{\boldsymbol{c}}}})$ is a learned likelihood with parameters $\widehat{{{{\boldsymbol{\eta }}}}}$ (see Supplementary Note 4 for the detailed derivation). Minimizing $\left[{\beta }^{s}I(s,{{{\boldsymbol{c}}}})+{\beta }^{x}I({{{\boldsymbol{x}}}},{{{\boldsymbol{u}}}})\right]$ is thus approximately equal to minimizing the IB loss ${\widehat{l}}^{{{{\rm{IB}}}}}$ with respect to ***ϕ*** for all cells,

24 $$\begin{array}{l}\displaystyle{\widehat{l}}^{{{{\rm{IB}}}}}({{{\boldsymbol{\phi }}}};{{{{\boldsymbol{x}}}}}_{n},{s}_{n},\widehat{{{{\boldsymbol{\eta }}}}})\\ \displaystyle \triangleq {\beta }^{s}{{\mathbb{E}}}_{{q}_{{{{\boldsymbol{\phi }}}}}({{{\boldsymbol{c}}}}| {{{{\boldsymbol{x}}}}}_{n},{s}_{n})}\left[\log {p}_{\widehat{{{{\boldsymbol{\eta }}}}}}({s}_{n}| {{{\boldsymbol{c}}}})\right]+{\beta }^{x}{{{\rm{KL}}}}\left[{q}_{{{{\boldsymbol{\phi }}}}}({{{\boldsymbol{u}}}}| {{{{\boldsymbol{x}}}}}_{n},{s}_{n})\parallel p({{{\boldsymbol{u}}}})\right]\\ \displaystyle -{\beta }^{x}{{\mathbb{E}}}_{{q}_{{{{\boldsymbol{\phi }}}}}({{{\boldsymbol{u}}}}| {{{{\boldsymbol{x}}}}}_{n},{s}_{n})}\left[\log {p}_{{{{\boldsymbol{\theta }}}}}({s}_{n}| {{{\boldsymbol{u}}}})\right]\end{array}$$

For ${p}_{\widehat{{{{\boldsymbol{\eta }}}}}}(s| {{{\boldsymbol{c}}}})$, we model it as ${p}_{\widehat{{{{\boldsymbol{\eta }}}}}}(s| {{{\boldsymbol{c}}}})={{\mathrm{Categorical}}}\,\left(s| {{{\boldsymbol{\kappa }}}}\right)$, where ${{{\boldsymbol{\kappa }}}}=r({{{\boldsymbol{c}}}};\widehat{{{{\boldsymbol{\eta }}}}})$ is the probability vector and *r* is a classifier neural network parameterized by $\widehat{{{{\boldsymbol{\eta }}}}}$. To learn the classifier, we minimize the following expected negative log-likelihood with respect to $\widehat{{{{\boldsymbol{\eta }}}}}$,

25 $$\begin{array}{rcl}\displaystyle{{\mathbb{E}}}_{p({{{\boldsymbol{c}}}},s)}\left[-\log {p}_{\widehat{{{{\boldsymbol{\eta }}}}}}(s| {{{\boldsymbol{c}}}})\right]&=&{{\mathbb{E}}}_{p({{{\boldsymbol{x}}}},s)}{{\mathbb{E}}}_{p({{{\boldsymbol{c}}}}| {{{\boldsymbol{x}}}},s)}\left[-\log {p}_{\widehat{{{{\boldsymbol{\eta }}}}}}(s| {{{\boldsymbol{c}}}})\right]\\ \displaystyle &\approx & \frac{1}{N}\mathop{\sum}\limits_{n}{{\mathbb{E}}}_{p({{{\boldsymbol{c}}}}| {{{{\boldsymbol{x}}}}}_{n},{s}_{n})}\left[-\log {p}_{\widehat{{{{\boldsymbol{\eta }}}}}}({s}_{n}| {{{\boldsymbol{c}}}})\right]\\ \displaystyle &\approx & \frac{1}{N}\mathop{\sum}\limits_{n}{{\mathbb{E}}}_{{q}_{{{{\boldsymbol{\phi }}}}}({{{\boldsymbol{c}}}}| {{{{\boldsymbol{x}}}}}_{n},{s}_{n})}\left[-\log {p}_{\widehat{{{{\boldsymbol{\eta }}}}}}({s}_{n}| {{{\boldsymbol{c}}}})\right]\end{array}$$

from which we can define a classifier loss

26 $$\begin{array}{rcl}\displaystyle{\widehat{l}}^{r}(\widehat{{{{\boldsymbol{\eta }}}}};{{{{\boldsymbol{x}}}}}_{n},{s}_{n},{{{\boldsymbol{\phi }}}})&\triangleq & {{\mathbb{E}}}_{{q}_{{{{\boldsymbol{\phi }}}}}({{{\boldsymbol{c}}}}| {{{{\boldsymbol{x}}}}}_{n},{s}_{n})}\left[-\log {p}_{\widehat{{{{\boldsymbol{\eta }}}}}}({s}_{n}| {{{\boldsymbol{c}}}})\right]\end{array}$$

To further enhance latent disentanglement for cross-modal inference, we also apply IBs on the data generated from our self-supervised tasks, that is, minimizing $\left[{\beta }^{s}I(s,{{{{\boldsymbol{c}}}}}^{m})+{\beta }^{x}I({{{{\boldsymbol{x}}}}}^{m},{{{{\boldsymbol{u}}}}}^{m})\right]$ for each modality *m*. Similar to Eqs. (23) and (24), this can be achieved by minimizing ${\widehat{l}}^{{{{\rm{IB}}}}}({{{\boldsymbol{\phi }}}};{{{{\boldsymbol{x}}}}}_{n}^{m},{s}_{n},{{{{\boldsymbol{\eta }}}}}^{m})$, where ***η***^*m*^ is the parameters of the classifier neural network *r*^*m*^ to generate the probability vector ***κ***^*m*^ = *r*^*m*^(***c***^*m*^; ***η***^*m*^) for the likelihood ${p}_{{{{{\boldsymbol{\eta }}}}}^{m}}(s| {{{{\boldsymbol{c}}}}}^{m})={{\mathrm{Categorical}}}\,\left(s| {{{{\boldsymbol{\kappa }}}}}^{m}\right)$. Together with the IB loss of Eq. (24), the total IB loss is defined as

27 $$\begin{array}{l}{l}^{{{{\rm{IB}}}}}({{{\boldsymbol{\phi }}}};{{{{\boldsymbol{x}}}}}_{n},{s}_{n},{{{\boldsymbol{\eta }}}})\triangleq {\widehat{l}}^{{{{\rm{IB}}}}}({{{\boldsymbol{\phi }}}};{{{{\boldsymbol{x}}}}}_{n},{s}_{n},\widehat{{{{\boldsymbol{\eta }}}}})+\mathop{\sum}\limits_{m\in {{{{\mathcal{M}}}}}_{n}}{\widehat{l}}^{{{{\rm{IB}}}}}({{{\boldsymbol{\phi }}}};{{{{\boldsymbol{x}}}}}_{n}^{m},{s}_{n},{{{{\boldsymbol{\eta }}}}}^{m})\end{array}$$

where ${{{\boldsymbol{\eta }}}}=\{\widehat{{{{\boldsymbol{\eta }}}}},{\{{{{{\boldsymbol{\eta }}}}}^{m}\}}_{m\in {{{\mathcal{M}}}}}\}$. To learn ${p}_{{{{{\boldsymbol{\eta }}}}}^{m}}(s| {{{{\boldsymbol{c}}}}}^{m})$, we can also minimize ${{\mathbb{E}}}_{p({{{{\boldsymbol{c}}}}}^{m},s)}\left[-\log {p}_{{{{{\boldsymbol{\eta }}}}}^{m}}(s| {{{{\boldsymbol{c}}}}}^{m})\right]$, which corresponds to minimizing ${\widehat{l}}^{r}({{{{\boldsymbol{\eta }}}}}^{m};{{{{\boldsymbol{x}}}}}_{n}^{m},{s}_{n},{{{\boldsymbol{\phi }}}})$ according to Eqs. (25) and (26). With the classifier loss of Eq. (26), we define the total classifier loss as

28 $$\begin{array}{l}{l}^{r}({{{\boldsymbol{\eta }}}};{{{{\boldsymbol{x}}}}}_{n},{s}_{n},{{{\boldsymbol{\phi }}}})={\widehat{l}}^{r}(\widehat{{{{\boldsymbol{\eta }}}}};{{{{\boldsymbol{x}}}}}_{n},{s}_{n},{{{\boldsymbol{\phi }}}})+\mathop{\sum}\limits_{m\in {{{{\mathcal{M}}}}}_{n}}{\widehat{l}}^{r}({{{{\boldsymbol{\eta }}}}}^{m};{{{{\boldsymbol{x}}}}}_{n}^{m},{s}_{n},{{{\boldsymbol{\phi }}}})\end{array}$$

### Training MIDAS

To train the encoders and decoders of MIDAS, considering the training objectives defined in Eqs. (8), (19) and (27), we minimize the following objective with respect to {***θ***, ***ϕ***} for all observations ${\{{{{{\boldsymbol{x}}}}}_{n},{s}_{n}\}}_{n\in {{{\mathcal{N}}}}}$:

29 $$\begin{array}{l}{l}^{f,g}({{{\boldsymbol{\theta }}}},{{{\boldsymbol{\phi }}}};{{{{\boldsymbol{x}}}}}_{n},{s}_{n},{{{\boldsymbol{\eta }}}})={l}^{{{{\rm{ELBO}}}}}({{{\boldsymbol{\theta }}}},{{{\boldsymbol{\phi }}}};{{{{\boldsymbol{x}}}}}_{n},{s}_{n})+{l}^{{{{\rm{mod}}}}}({{{\boldsymbol{\phi }}}};{{{{\boldsymbol{x}}}}}_{n},{s}_{n})+{l}^{{{{\rm{IB}}}}}({{{\boldsymbol{\phi }}}};{{{{\boldsymbol{x}}}}}_{n},{s}_{n},{{{\boldsymbol{\eta }}}})\end{array}$$

Here, the loss *l*^ELBO^ is defined based on the negative of the ELBO of Eq. (8), that is,

30 $$\begin{array}{rcl}\displaystyle{l}^{{{{\rm{ELBO}}}}}({{{\boldsymbol{\theta }}}},{{{\boldsymbol{\phi }}}};{{{{\boldsymbol{x}}}}}_{n},{s}_{n})&\triangleq &-{{\mathbb{E}}}_{{q}_{{{{\boldsymbol{\phi }}}}}({{{\boldsymbol{c}}}},{{{\boldsymbol{u}}}}| {{{{\boldsymbol{x}}}}}_{n},{s}_{n})}\left[\gamma \log {p}_{{{{\boldsymbol{\theta }}}}}({s}_{n}| {{{\boldsymbol{u}}}})+\mathop{\sum}\limits_{m\in {{{{\mathcal{M}}}}}_{n}}\log {p}_{{{{\boldsymbol{\theta }}}}}({{{{\boldsymbol{x}}}}}_{n}^{m}| {{{\boldsymbol{c}}}},{{{\boldsymbol{u}}}})\right]\\ \displaystyle &&+{{{\rm{KL}}}}\left[{q}_{{{{\boldsymbol{\phi }}}}}({{{\boldsymbol{c}}}},{{{\boldsymbol{u}}}}| {{{{\boldsymbol{x}}}}}_{n},{s}_{n})\parallel p({{{\boldsymbol{c}}}},{{{\boldsymbol{u}}}})\right]\end{array}$$

where *γ* ≥ 1 is an additional weight that can be set to a higher value to encourage ***u*** to encode more batch-specific information. In Eq. (29), because the classifier parameters ***η*** are unknown and the learning of ***η*** depends on ***ϕ***, as in Eq. (28), we iteratively minimize Eqs. (28) and (29) with stochastic gradient descent (SGD), forming the MIDAS training algorithm (Algorithm 1). To better guide the optimization of the IB loss in Eq. (29) for disentangling latent variables, we increase the number of updates (that is, with *K* > 1) of Eq. (28) for the classifier parameters ***η*** in each iteration to ensure that these classifiers stay close to their optimal solutions.

**Algorithm 1.** The MIDAS training algorithm.

**Input**: A single-cell multimodal mosaic dataset ${\{{{{{\boldsymbol{x}}}}}_{n},{s}_{n}\}}_{n\in {{{\mathcal{N}}}}}$

**Output**: Decoder parameters ***θ***, encoder parameters ***ϕ*** and classifier parameters ***η***

(1) Randomly initialize parameters {***θ***, ***ϕ***, ***η***}

(2) **for** iteration *t* = 1, 2, …, *T* **do**

(3)  Sample a minibatch ${\{{{{{\boldsymbol{x}}}}}_{n},{s}_{n}\}}_{n\in {{{{\mathcal{N}}}}}_{t}}$ from the dataset, where ${{{{\mathcal{N}}}}}_{t}\subset {{{\mathcal{N}}}}$

(4)  **for** step *k* = 1, 2, …, *K* **do**

(5)   Freeze ***ϕ*** and update ***η*** via SGD with loss $\frac{1}{| {{{{\mathcal{N}}}}}_{t}| }{\sum }_{n\in {{{{\mathcal{N}}}}}_{t}}{l}^{r}({{{\boldsymbol{\eta }}}};{{{{\boldsymbol{x}}}}}_{n},{s}_{n},{{{\boldsymbol{\phi }}}})$  ⊳ See Eq. (28)

(6)  **end** **for**

(7)  Freeze ***η*** and update {***θ***, ***ϕ***} via SGD with loss $\displaystyle\frac{1}{| {{{{\mathcal{N}}}}}_{t}| }{\sum }_{n\in {{{{\mathcal{N}}}}}_{t}}{l}^{f,g}({{{\boldsymbol{\theta }}}},{{{\boldsymbol{\phi }}}};{{{{\boldsymbol{x}}}}}_{n},{s}_{n},{{{\boldsymbol{\eta }}}})$  ⊳ See Eq. (29)

(8) **end** **for**

### Mosaic integration on latent space

A key goal of single-cell mosaic integration is to extract biologically meaningful low-dimensional cell embeddings from the mosaic data for downstream analysis, where the technical variations are removed. To achieve this, for each cell, we first use the trained MIDAS to infer the latent posterior *q*_***ϕ***_(***c***, ***u***|***x***_*n*_, *s*_*n*_) through Eq. (10), obtaining the mean ${{{{\boldsymbol{\mu }}}}}_{n}=\{{{{{\boldsymbol{\mu }}}}}_{n}^{c},{{{{\boldsymbol{\mu }}}}}_{n}^{u}\}$ and variance ${{{{\boldsymbol{\nu }}}}}_{n}=\{{{{{\boldsymbol{\nu }}}}}_{n}^{c},{{{{\boldsymbol{\nu }}}}}_{n}^{u}\}$. We then take the maximum a posteriori (MAP) estimation of {***c***, ***u***} as the integration result on the latent space, which is the mean ***μ***_*n*_ because *q*_***ϕ***_(***c***, ***u***|***x***_*n*_, *s*_*n*_) is Gaussian. Finally, we take ${{{{\boldsymbol{\mu }}}}}_{n}^{c}$, the MAP estimation of ***c***, as the cell embedding.

### Imputation for missing modalities and features

Based on the MAP estimation $\{{{{{\boldsymbol{\mu }}}}}_{n}^{c},{{{{\boldsymbol{\mu }}}}}_{n}^{u}\}$ inferred from the single-cell mosaic data (see ‘Mosaic integration on latent space’), it is straightforward to impute missing modalities and features. We first pass $\{{{{{\boldsymbol{\mu }}}}}_{n}^{c},{{{{\boldsymbol{\mu }}}}}_{n}^{u}\}$ to the decoders to generate padded feature mean ${\widetilde{{{{\boldsymbol{\lambda }}}}}}_{n}^{m}$ for each modality $m\in {{{\mathcal{M}}}}$ via Eq. (18). We then sample from a Bernoulli distribution with mean ${\widetilde{{{{\boldsymbol{\lambda }}}}}}_{n}^{{{{\rm{ATAC}}}}}$ to generate the imputed ATAC counts and from two Poisson distributions with means ${\widetilde{{{{\boldsymbol{\lambda }}}}}}_{n}^{{{{\rm{RNA}}}}}$ and ${\widetilde{{{{\boldsymbol{\lambda }}}}}}_{n}^{{{{\rm{ADT}}}}}$ to generate the imputed RNA and ADT counts, respectively.

### Batch correction via latent variable manipulation

Besides performing mosaic integration on the latent space (see ‘Mosaic integration on latent space’), we can also perform it on the feature space, that is, imputing missing values and correcting batch effects for the count data. Mosaic integration on feature space is important because it is required by many downstream tasks, such as cell typing and trajectory inference.

With the latent variables’ MAP estimation $\{{{{{\boldsymbol{\mu }}}}}_{n}^{c},{{{{\boldsymbol{\mu }}}}}_{n}^{u}\}$, we can perform imputation and batch correction simultaneously by manipulating the technical noise. Concretely, let ${{{{\boldsymbol{c}}}}}_{n}={{{{\boldsymbol{\mu }}}}}_{n}^{c}$ and ${{{{\boldsymbol{u}}}}}_{n}={{{{\boldsymbol{\mu }}}}}_{n}^{u}$. We first calculate the mean of ***u***_*n*_ within each batch $b\in {{{\mathcal{B}}}}$

31 $$\begin{array}{ll}{\bar{{{{\boldsymbol{u}}}}}}_{b}&=\frac{1}{| {{{{\mathcal{N}}}}}_{b}| }\mathop{\sum}\limits_{n\in {{{{\mathcal{N}}}}}_{b}}{{{{\boldsymbol{u}}}}}_{n}\end{array}$$

where ${{{{\mathcal{N}}}}}_{b}\subseteq {{{\mathcal{N}}}}$ is the set of cell IDs belonging to batch *b*. Next, we calculate the mean of ${\bar{{{{\boldsymbol{u}}}}}}_{b}$ over all batches

32 $$\begin{array}{ll}\bar{{{{\boldsymbol{u}}}}}&=\frac{1}{B}\mathop{\sum}\limits_{b}{\bar{{{{\boldsymbol{u}}}}}}_{b}\end{array}$$

We then look for the batch b^*^ with a mean ${\bar{{{{\boldsymbol{u}}}}}}_{{b}^{* }}$ closest to $\bar{{{{\boldsymbol{u}}}}}$ and treat ${\bar{{{{\boldsymbol{u}}}}}}_{{b}^{* }}$ as the ‘standard’ technical noise, where

33 $$\begin{array}{ll}{b}^{* }&=\arg \mathop{\min }\limits_{b}\parallel {\bar{{{{\boldsymbol{u}}}}}}_{b}-\bar{{{{\boldsymbol{u}}}}}{\parallel }_{2}\end{array}$$

Finally, for each cell, we correct the batch effect by substituting ***u***_*n*_ with ${\bar{{{{\boldsymbol{u}}}}}}_{{b}^{* }}$ and pass $\{{{{{\boldsymbol{c}}}}}_{n},{\bar{{{{\boldsymbol{u}}}}}}_{{b}^{* }}\}$ to the decoders to generate imputed and batch-corrected data (similar to ‘Imputation for missing modalities and features’, but here we use $\{{{{{\boldsymbol{c}}}}}_{n},{\bar{{{{\boldsymbol{u}}}}}}_{{b}^{* }}\}$ instead of {***c***_*n*_, ***u***_*n*_} to correct the batch effect).

### Model transfer via transfer learning

When MIDAS has been pretrained on a reference dataset, we can conduct model transfer to transfer the model’s learned knowledge to a query dataset through transfer learning; that is, on the query dataset, we fine-tune the pretrained model instead of train the model from scratch. Because, compared to the reference dataset, the query dataset can contain different numbers of batches collected from different platforms, the batch ID-related modules need to be redefined. Thus, during transfer learning, we reparameterize and reinitialize the batch ID encoder and decoder {*f*^*s*^, *g*^*s*^} and the batch classifiers $\{r,{\{{r}^{m}\}}_{m\in {{{\mathcal{M}}}}}\}$ and only fine-tune the modality encoders and decoders ${\{{f}^{m},{g}^{m}\}}_{m\in {{{\mathcal{M}}}}}$.

A core advantage of our model transfer scheme is that it can flexibly transfer the knowledge of multimodal data to various types of query datasets, even to those with fewer modalities, improving the de novo integration of single-cell data.

### Label transfer via reciprocal reference mapping and kNN-based cell annotation

While model transfer implicitly transfers knowledge through model parameters, label transfer explicitly transfers knowledge in the form of data labels. These labels can be different kinds of downstream analysis results, such as cell types, cell cycles or pseudotime. Through accurate label transfer, we can not only avoid the expensive de novo integration and downstream analysis but also improve label quality.

#### Reciprocal reference mapping

Typically, the first step of label transfer is reference mapping, which aligns the query cells with the reference cells so that labels can be transferred reliably. For MIDAS, we can naively achieve reference mapping in two ways: (1) mapping the query data onto the reference space (that is, applying the model pretrained on the reference data to infer the biological states for the query data^50,51^) and (2) mapping the reference data onto the query space (that is, applying the model fine-tuned on the query data (see ‘Model transfer via transfer learning’) to infer the biological states for the reference data^14,52^). However, the first way suffers from the ‘generalization problem’ because the pretrained model is hard to generalize to the query data, which usually contains unseen technical variations, whereas the second way suffers from the ‘forgetting problem’ because the fine-tuned model may lose information learned on the reference data, affecting the inferred biological states.

To tackle both problems, we propose a reciprocal reference mapping scheme, where we fine-tune the pretrained model on the query dataset to avoid the generalization problem and meanwhile feed the model with the historical data sampled from the reference dataset to prevent forgetting. In doing this, the model can find a mapping suitable for both reference and query datasets and can then align them on the latent space by inferring their biological states.

#### kNN-based cell annotation with novel cell-type identification

Based on the aligned latent representations (embeddings), the kNN classifier is used to transfer the reference labels to the query dataset. When the query and reference datasets belong to the same tissue (for example, PBMCs), we train the kNN classifier using the reference embeddings and labels and then use it to classify the query cells.

However, if the query and reference datasets are from distinct tissues (for example, BMMCs versus PBMCs), we might encounter new cell types in the query dataset that are not present in the reference dataset. To address this issue, we propose a strategy for novel cell-type detection. Specifically, we assign the label ‘query’ to all the query cells and use the cell embeddings and labels from both the query and reference datasets to train the kNN classifier. Subsequently, we use the classifier to predict the class probabilities for the query cells.

To detect new cell types, we use a thresholding approach on the predicted probabilities of the ‘query’ class, that is, we leverage a Gaussian mixture model with two components to group the probabilities into two distinct clusters. This clustering process allows us to establish a suitable threshold for the probabilities. For the cluster with a higher mean, its cells have higher probabilities belonging to the ‘query’ class; we consider these cells as unique to the query dataset and assign them the label ‘unknown’. Conversely, for the cluster with a lower mean, its cells have lower probabilities belonging to the ‘query’ class; we consider these cells to belong to the types present in the reference dataset and assign each of these cells the label of the class with the highest predicted probability among all classes except the ‘query’ class.

The above kNN and Gaussian mixture model algorithms are implemented by the KNeighborsClassifier function (n_neighbors = 100 and weights = ‘uniform’) and the GaussianMixture function (n_components = 2 and tol = 10^−4^) from the scikit-learn^58^ (v1.2.2) Python package, respectively. Similar to model transfer (see ‘Model transfer via transfer learning’), in label transfer, knowledge can also be flexibly and accurately transferred to various types of query datasets.

### Modality contribution to the integrated clustering

We assess the contribution of different modalities to clustering by measuring the agreement between single-modality clustering and multimodalities cell clustering. For each cell, the normalized consistency ratio of the nearest neighbors in the single modal clustering and multimodalities clustering is used to represent contribution of the modal for the final integrated clustering.

### Regulatory network inference from scRNA-seq datasets

The GRNBoost2 algorithm^59^ from the Arboreto (v0.1.5) Python package is used to infer the regulatory network from scRNA-seq datasets. Weighted regulatory links between genes and transcription factors are provided from GRNBoost2. The weights of shared links from different data are compared to indicate the regulatory network retention.

### Correlation of expression fold change values between raw and batch-corrected data

For each cell type, expression fold change values of genes and proteins are calculated against all other cells using the FoldChange function in the Seurat (v4.3.0) R package. The Pearson correlation coefficient is used to measure linear correlations of fold change values between raw and batch-corrected data.

### Generating Seurat cell-type labels

To generate cell-type labels for both qualitative and quantitative evaluation, we used the third-party tool Seurat to annotate cell types for different datasets through label transfer. We took the CITE-seq PBMC atlas from Hao et al.^15^ as the reference set and used the FindTransferAnchors and TransferData functions in Seurat to perform label transfer, where ‘cca’ was used as the reduction method for reference mapping. For cells without raw RNA expression, we first used ATAC data to create a gene activity matrix using the GeneActivity function in the Signac^60^ (v1.9.0) R package. The gene activity matrix was subsequently used for label transfer.

### Evaluation metrics

To evaluate the performance of MIDAS and the state-of-the-art tools on multimodal integration, we use metrics from scIB on batch correction and biological conservation and also propose our own metrics on modality alignment to better evaluate mosaic integration, extending scIB to scMIB (Supplementary Table 4). Because mosaic integration should generate low-dimensional representations and the imputed and batch-corrected data, scMIB is performed on both embedding space and feature space. To evaluate the batch correction and biological conservation metrics on the feature space, we convert the imputed and batch-corrected feature into a similarity graph via the PCA+WNN strategy (see ‘Implementation of comparing methods’) and then use this graph for evaluation. Our metrics for batch correction, modality alignment and biological conservation are defined as follows.

#### Batch correction metrics

The batch correction metrics comprise graph integration local inverse Simpson’s index (iLISI; ${y}_{{{{\rm{embed}}}}}^{{{{\rm{i}}}}{{{\rm{LISI}}}}}$ and ${y}_{{{{\rm{feat}}}}}^{{{{\rm{i}}}}{{{\rm{LISI}}}}}$), graph connectivity (${y}_{{{{\rm{embed}}}}}^{{{{\rm{gc}}}}}$ and ${y}_{{{{\rm{feat}}}}}^{{{{\rm{gc}}}}}$) and kNN batch effect test (kBET; ${y}_{{{{\rm{embed}}}}}^{{{{\rm{k}}}}{{{\rm{BET}}}}}$ and ${y}_{{{{\rm{feat}}}}}^{{{{\rm{k}}}}{{{\rm{BET}}}}}$), where ${y}_{{{{\rm{embed}}}}}^{{{{\rm{i}}}}{{{\rm{LISI}}}}}$, ${y}_{{{{\rm{embed}}}}}^{{{{\rm{gc}}}}}$ and ${y}_{{{{\rm{embed}}}}}^{{{{\rm{k}}}}{{{\rm{BET}}}}}$ are defined in embedding space and ${y}_{{{{\rm{feat}}}}}^{{{{\rm{i}}}}{{{\rm{LISI}}}}}$, ${y}_{{{{\rm{feat}}}}}^{{{{\rm{gc}}}}}$ and ${y}_{{{{\rm{feat}}}}}^{{{{\rm{k}}}}{{{\rm{BET}}}}}$ are defined in feature space.

##### Graph iLISI

The graph iLISI metric is extended from the iLISI^61^, which is used to measure the batch mixing degree. The iLISI scores are computed based on kNN graphs by computing the inverse Simpson’s index for diversity. The scores estimate the effective number of batches present in the neighborhood. iLISI ranges from 1 to *N*, where *N* equals the number of batches. Scores close to the real batch numbers denote good mixing. However, the typical iLISI score is not applicable to graph-based outputs. scIB proposed the graph iLISI, which uses a graph-based distance metric to determine the nearest neighbor list and avoids skews on graph-based integration outputs. The graph iLISI scores are scaled to [0, 1], where 0 indicates strong separation, and 1 indicates perfect mixing.

##### Graph connectivity

Graph connectivity is proposed by scIB to inspect whether cells with the same label are connected in the kNN graph of all cells. For each label *c*, we get the largest connected graph component of *c*-labeled cells and divide the largest connected graph component size by the population size of *c*-labeled cells to represent the graph connectivity for cell label *c*. We then calculate the connectivity values for all labels and take the average as the total graph connectivity. The score ranges from 0 to 1. A score of 1 means that all cells with the same cell identity from different batches are connected in the integrated kNN graph, which also indicates the perfect batch mixing and vice versa.

##### kBET

The kBET^62^ is used to measure batch mixing at the local level of the kNN. Certain fractions of random cells are repeatedly selected to test whether the local label distributions are statistically similar to the global label distributions (null hypothesis). The kBET value is the rejection rate over all tested neighborhoods, and values close to 0 indicate that the batches are well mixed. scIB adjusts the kBET with a diffusion-based correction to enable unbiased comparison on graph- and non-graph-based integration results. kBET values are first computed for each label and then averaged and subtracted from 1 to get a final kBET score.

#### Modality alignment metrics

The modality alignment metrics comprise modality averaged silhouette width (ASW; *y*^ASW^), fraction of samples closer than the true match (FOSCTTM; *y*^FOSCTTM^), label transfer F1 (*y*^ltF1^), ATAC area under the receiver operating characteristic (AUROC; *y*^AUROC^), RNA Pearson’s *r* (*y*^RNAr^) and ADT Pearson’s *r* (*y*^ADTr^), where *y*^ASW^, *y*^FOSCTTM^ and *y*^ltF1^ are defined in embedding space, and *y*^AUROC^, *y*^RNAr^ and *y*^ADTr^ are defined in feature space.

##### Modality ASW

The modality ASW is used to measure the alignment of distributions between different modality embeddings. The ASW^63^ is originally used to measure the separation of clusters. In scIB, ASW is also modified to measure the performance of batch effect removal, resulting in a batch ASW that ranges from 0 to 1, where 1 denotes perfect batch mixing, and 0 denotes strong batch separation. By replacing batch embeddings with modality embeddings, we can define a modality ASW in the same manner as the batch ASW, where 1 denotes perfect modality alignment, and 0 denotes strong modality separation. For MIDAS, the modality embeddings are generated by feeding the trained model with each modality individually.

##### FOSCTTM

The FOSCTTM^64^ is used to measure the alignment of values between different modality embeddings. Let ${y}_{{m}_{1},{m}_{2}}^{{{{\rm{FOSCTTM}}}}}$ be the FOSCTTM for a modality pair {*m*_1_, *m*_2_}; it is defined as

34 $$\begin{array}{ll}{y}_{{m}_{1},{m}_{2}}^{{{{\rm{FOSCTTM}}}}}&=\frac{1}{2N}\left(\mathop{\sum}\limits_{i}\frac{{N}_{i}^{{m}_{1}}}{N}+\mathop{\sum}\limits_{i}\frac{{N}_{i}^{{m}_{2}}}{N}\right)\\ {N}_{i}^{{m}_{1}}&=| \left\{j| \parallel {{{{\boldsymbol{e}}}}}_{i}^{{m}_{1}}-{{{{\boldsymbol{e}}}}}_{j}^{{m}_{2}}{\parallel }_{2} < \parallel {{{{\boldsymbol{e}}}}}_{i}^{{m}_{1}}-{{{{\boldsymbol{e}}}}}_{i}^{{m}_{2}}{\parallel }_{2}\right\}| \\ {N}_{i}^{{m}_{2}}&=| \left\{j| \parallel {{{{\boldsymbol{e}}}}}_{j}^{{m}_{1}}-{{{{\boldsymbol{e}}}}}_{i}^{{m}_{2}}{\parallel }_{2} < \parallel {{{{\boldsymbol{e}}}}}_{i}^{{m}_{1}}-{{{{\boldsymbol{e}}}}}_{i}^{{m}_{2}}{\parallel }_{2}\right\}| \end{array}$$

where *N* is the number of cells, *i* and *j* are the cell indices, and ${{{{\boldsymbol{e}}}}}_{i}^{{m}_{1}}$ and ${{{{\boldsymbol{e}}}}}_{i}^{{m}_{2}}$ are the embeddings of cell *i* in modalities *m*_1_ and *m*_2_, respectively. ${N}_{i}^{{m}_{1}}$ is the number of cells in modality *m*_2_ that are closer to ${{{{\boldsymbol{e}}}}}_{i}^{{m}_{1}}$ than ${{{{\boldsymbol{e}}}}}_{i}^{{m}_{2}}$ is to ${{{{\boldsymbol{e}}}}}_{i}^{{m}_{1}}$, and it is similar for ${N}_{i}^{{m}_{2}}$. We first get the embeddings of individual modalities, calculate the FOSCTTM values for each modality pair and then average these values and subtract it from 1 to obtain a final FOSCTTM score. Higher FOSCTTM scores indicate better modality alignment.

##### Label transfer F1

The label transfer F1 is used to measure the alignment of cell types between different modality embeddings. This can be achieved by testing whether cell-type labels can be transferred from one modality to another without any bias. For each pair of modalities, we first build a kNN graph between their embeddings and then transfer labels from one modality to the other based on the nearest neighbors. The transferred labels are compared to the original labels by the micro F1 score, which is defined as the label transfer F1. We take the F1 score averaged from all comparison pairs as the final label transfer F1 score.

##### ATAC AUROC

The ATAC AUROC is used to measure the alignment of different modalities in the ATAC feature space. It has been previously used to evaluate the quality of ATAC predictions^65^. For each method to be evaluated, we first use it to convert different modality combinations that do not contain ATAC into ATAC features, respectively, calculate the AUROC of each converted result by taking the true ATAC features as the ground truth and finally take the average of these AUROCs as the final score. Taking MIDAS as an example, if ATAC, RNA and ADT data are involved, the evaluation is based on the combinations {RNA}, {ADT} and {RNA, ADT}. For each combination, we feed the data into the trained model to generate the imputed data of all modalities {ATAC, RNA, ADT} (see, ‘Imputation for missing modalities and features’), where the generated ATAC features are used for AUROC calculation.

##### RNA Pearson’s *r*

The RNA Pearson’s *r* value is used to measure the alignment of different modalities in the RNA feature space. For each method to be evaluated, we first use it to convert different modality combinations that do not contain RNA into RNA features, respectively, calculate the Pearson’s *r* value between each converted result and the true RNA features and finally take the average of these Pearson’s *r* values as the final score.

##### ADT Pearson’s *r*

The ADT Pearson’s *r* value is used to measure the alignment of different modalities in the ADT feature space. The calculation of the ADT Pearson’s *r* value is similar to that of the RNA Pearson’s *r* value.

#### Biological conservation metrics

The biological conservation metrics comprise normalized MI (NMI; ${y}_{{{{\rm{embed}}}}}^{{{{\rm{NMI}}}}}$ and ${y}_{{{{\rm{feat}}}}}^{{{{\rm{NMI}}}}}$), adjusted Rand index (ARI; ${y}_{{{{\rm{embed}}}}}^{{{{\rm{ARI}}}}}$ and ${y}_{{{{\rm{feat}}}}}^{{{{\rm{ARI}}}}}$), isolated label F1 (${y}_{{{{\rm{embed}}}}}^{{{{\rm{il}}}}{{{\rm{F}}}}1}$ and ${y}_{{{{\rm{feat}}}}}^{{{{\rm{il}}}}{{{\rm{F}}}}1}$) and graph cell-type LISI (cLISI; ${y}_{{{{\rm{embed}}}}}^{{{{\rm{c}}}}{{{\rm{LISI}}}}}$ and ${y}_{{{{\rm{feat}}}}}^{{{{\rm{c}}}}{{{\rm{LISI}}}}}$), where ${y}_{{{{\rm{embed}}}}}^{{{{\rm{NMI}}}}}$, ${y}_{{{{\rm{embed}}}}}^{{{{\rm{ARI}}}}}$, ${y}_{{{{\rm{embed}}}}}^{{{{\rm{il}}}}{{{\rm{F}}}}1}$ and ${y}_{{{{\rm{embed}}}}}^{{{{\rm{c}}}}{{{\rm{LISI}}}}}$ are defined in embedding space, and ${y}_{{{{\rm{feat}}}}}^{{{{\rm{NMI}}}}}$, ${y}_{{{{\rm{feat}}}}}^{{{{\rm{ARI}}}}}$, ${y}_{{{{\rm{feat}}}}}^{{{{\rm{il}}}}{{{\rm{F}}}}1}$ and ${y}_{{{{\rm{feat}}}}}^{{{{\rm{c}}}}{{{\rm{LISI}}}}}$ are defined in feature space.

##### NMI

The NMI is used to measure the similarity between two clustering results, namely the predefined cell-type labels and the clustering result obtained from the embeddings or the graph. Optimized Louvain clustering is used here according to scIB. The NMI scores are scaled to [0, 1], where 0 and 1 correspond to uncorrelated clustering and a perfect match, respectively.

##### ARI

The ARI also measures the overlap of two clustering results. The Rand index (RI^66^) considers not only cell pairs that are assigned in the same clusters but also ones in different clusters in the predicted (Louvain clustering) and true (cell-type) clusters. The ARI corrects the RI for randomly correct labels. An ARI of 1 represents a perfect match, and 0 represents random labeling.

##### Isolated label F1

scIB proposes the isolated label F1 score to evaluate integration performance, specifically focusing on cells with the label that is shared by few batches. Cell labels presented in the least number of batches are identified as isolated labels. The F1 score for measuring the clustering performance on isolated labels is defined as the isolated label F1 score. It reflects how well the isolated labels separate from other cell identities, ranging from 0 to 1, where 1 means all the isolated label cells and no others are grouped into one cluster.

##### Graph cLISI

The graph cLISI is similar to the graph iLISI but focuses on cell-type labels rather than batch labels. Unlike iLISI that highlights the mixing of groups, cLISI values the separation of groups^61^. The graph-adjusted cLISI is scaled to [0, 1], with a value of 0 corresponding to low cell-type separation and a value of 1 corresponding to strong cell-type separation.

#### Overall scores

##### scIB

We compute the scIB overall score using the batch correction and biological conservation metrics defined either on the embedding space (for algorithms generating embeddings or graphs) or the feature space (for algorithms generating batch-corrected features). Following Luecken et al.^43^, the overall score *y* is the sum of the averaged batch correction metric *y*^batch^ weighted by 0.4 and the averaged biological conservation metric *y*^bio^ weighted by 0.6,

35 $$\begin{array}{ll}{y}^{{{{\rm{batch}}}}}&=({y}_{\omega }^{{{{\rm{i}}}}{{{\rm{LISI}}}}}+{y}_{\omega }^{{{{\rm{gc}}}}}+{y}_{\omega }^{{{{\rm{k}}}}{{{\rm{BET}}}}})/3\\ {y}^{{{{\rm{bio}}}}}&=({y}_{\omega }^{{{{\rm{NMI}}}}}+{y}_{\omega }^{{{{\rm{ARI}}}}}+{y}_{\omega }^{{{{\rm{il}}}}{{{\rm{F}}}}1}+{y}_{\omega }^{{{{\rm{c}}}}{{{\rm{LISI}}}}})/4\\ y&=0.4\cdot {y}^{{{{\rm{batch}}}}}+0.6\cdot {y}^{{{{\rm{bio}}}}}\end{array}$$

where *ω* = embed for embedding or graph outputs, and *ω* = feat for feature outputs.

##### scMIB

As an extension of scIB, the scMIB overall score *y* is computed from the batch correction, modality alignment and biological conservation metrics defined on both the embedding and feature space. It is the sum of the averaged batch correction metric *y*^batch^ weighted by 0.3, the averaged modality alignment metric ${y}^{{{{\rm{mod}}}}}$ weighted by 0.3 and the averaged biological conservation metric *y*^bio^ weighted by 0.4:

36 $$\begin{array}{ll}{y}^{{{{\rm{batch}}}}}=({y}_{{{{\rm{embed}}}}}^{{{{\rm{i}}}}{{{\rm{LISI}}}}}+{y}_{{{{\rm{embed}}}}}^{{{{\rm{gc}}}}}+{y}_{{{{\rm{embed}}}}}^{{{{\rm{k}}}}{{{\rm{BET}}}}}+{y}_{{{{\rm{feat}}}}}^{{{{\rm{i}}}}{{{\rm{LISI}}}}}+{y}_{{{{\rm{feat}}}}}^{{{{\rm{gc}}}}}+{y}_{{{{\rm{feat}}}}}^{{{{\rm{k}}}}{{{\rm{BET}}}}})/6\\ {y}^{{{{\rm{mod}}}}}=({y}^{{{{\rm{ASW}}}}}+{y}^{{{{\rm{FOSCTTM}}}}}+{y}^{{{{\rm{lt}}}}{{{\rm{F}}}}1}+{y}^{{{{\rm{AUROC}}}}}+{y}^{{{{\rm{RNA}}}}{{{\rm{r}}}}}+{y}^{{{{\rm{ADT}}}}{{{\rm{r}}}}})/6\\ {y}^{{{{\rm{bio}}}}}=({y}_{{{{\rm{embed}}}}}^{{{{\rm{NMI}}}}}+{y}_{{{{\rm{embed}}}}}^{{{{\rm{ARI}}}}}+{y}_{{{{\rm{embed}}}}}^{{{{\rm{il}}}}{{{\rm{F}}}}1}+{y}_{{{{\rm{embed}}}}}^{{{{\rm{c}}}}{{{\rm{LISI}}}}}+{y}_{{{{\rm{feat}}}}}^{{{{\rm{NMI}}}}}+{y}_{{{{\rm{feat}}}}}^{{{{\rm{ARI}}}}}+{y}_{{{{\rm{feat}}}}}^{{{{\rm{il}}}}{{{\rm{F}}}}1}+{y}_{{{{\rm{feat}}}}}^{{{{\rm{c}}}}{{{\rm{LISI}}}}})/8\\ y=0.3\cdot {y}^{{{{\rm{batch}}}}}+0.3\cdot {y}^{{{{\rm{mod}}}}}+0.4\cdot {y}^{{{{\rm{bio}}}}}\end{array}$$

### Datasets

All datasets of human PBMCs were publicly available (Supplementary Table 1). Count matrices of gene unique molecular identifiers (UMIs), ATAC fragments and ADTs were downloaded for data analysis.

#### DOGMA dataset

The DOGMA dataset contains four batches profiled by DOGMA-seq, which measures RNA, ATAC and ADT data simultaneously. Trimodal data from this dataset were obtained from Gene Expression Omnibus (GEO)^67^ under accession ID GSE166188 (ref. ^3^).

#### TEA dataset

The TEA dataset contains five batches profiled by TEA-seq, which measures RNA, ATAC and ADT data simultaneously. Trimodal data from these batches were obtained from GEO under accession ID GSE158013 (ref. ^4^).

#### TEA Multiome dataset

The TEA Multiome dataset measuring paired RNA and ATAC data was obtained from GEO under accession ID GSE158013 (ref. ^4^). This dataset contains two batches profiled by 10x Chromium Single Cell Multiome ATAC + Gene Expression.

#### 10x Multiome dataset

The 10x Multiome dataset measuring paired RNA and ATAC data was collected from 10x Genomics (https://www.10xgenomics.com/resources/datasets/)^68–71^.

#### ASAP dataset

The ASAP dataset was obtained from GEO under accession ID GSE156473 (ref. ^3^). Two batches profiled by ASAP-seq are used, which include ATAC and ADT data.

#### ASAP CITE dataset

The ASAP CITE dataset was obtained from GEO under accession ID GSE156473 (ref. ^3^). Two batches profiled by CITE-seq are used, which include RNA and ADT data.

#### WNN CITE dataset

The WNN CITE dataset measuring paired RNA and ADT data was obtained from https://atlas.fredhutch.org/nygc/multimodal-pbmc ref. ^15^. This dataset was profiled by CITE-seq. We selected the eight PBMC batches generated before the administration of HIV vaccine for integration.

#### BMMC mosaic dataset

The BMMC mosaic dataset included three batches. The ICA batch measuring RNA data was obtained from https://www.dropbox.com/s/xe5tithw1xjxrfs/ica_bone_marrow.h5?dl=0 (ref. ^72^), where the first batch (‘MantonBM1’) of the original data is used. The ASAP batch measuring ADT and ATAC data was obtained from GEO under accession ID GSE156477 (ref. ^3^). The CITE batch measuring RNA and ADT data was obtained from GEO under accession ID GSE128639 (ref. ^13^).

### Data preprocessing

The count matrices of RNA and ADT were processed via Seurat. The ATAC fragment files were processed using Signac, and peaks were called via the Python package MACS2 (ref. ^73^; v2.2.7.1). We performed quality control separately for each batch. Briefly, metrics of detected gene number per cell, total UMI number, percentage of mitochondrial RNA reads, total protein tag number, total fragment number, transcription start site score and nucleosome signal were evaluated. We manually checked the distributions of these metrics and set customized criteria to filter low-quality cells in each batch. The number of cells that passed quality control in each batch is shown in Supplementary Table 1.

For each batch, we adopted common normalization strategies for RNA, ADT and ATAC data, respectively. Specifically, for RNA data, UMI count matrices are normalized and log transformed using the NormalizeData function in Seurat. For ADT data, tag count matrices are centered log ratio normalized using the NormalizeData function in Seurat. For ATAC data, fragment matrices are term frequency inverse document frequency normalized using the RunTFIDF function in Signac.

To integrate batches profiled by various technologies, we need to create a union of features for RNA, ADT and ATAC data, respectively. For RNA data, first, low-frequency genes are removed based on gene occurrence frequency across all batches. We then select 4,000 highly variable genes using the FindVariableFeatures function with default parameters in each batch. The union of these highly variable genes is ranked using the SelectIntegrationFeatures function, and the top 4,000 genes are selected. In addition, we also retain genes that encode proteins targeted by the antibodies. For ADT data, the union of antibodies in all batches is retained for data integration. For ATAC data, we used the reduce function in Signac to merge all intersecting peaks across batches and then recalculated the fragment counts in the merged peaks. The merged peaks are used for data integration.

The input data for MIDAS are UMI counts for RNA data, tag counts for ADT data and binarized fragment counts for ATAC data. For each modality, the union of features from all batches are used. Counts of missing features are set to 0. Binary feature masks are generated accordingly, where 1 and 0 denote presented and missing features, respectively.

### Implementation of MIDAS

We implement the MIDAS architecture using PyTorch^74^. The sizes of the shared hidden layers for different modality encoders are set to 1,024–128, whereas the sizes of the shared hidden layers for different modality decoders are set to 128–1,024. Additionally, the sizes of the biological state and technical noise latent variables are set to 32 and 2, respectively (refer to Supplementary Fig. 1 and Supplementary Table 12 for details). Each hidden layer is constructed using four PyTorch modules: Linear, LayerNorm, Mish and Dropout. The input and output layers have different sizes depending on the datasets used (refer to Supplementary Table 13 for details). To effectively reduce the number of model parameters, similar to Wu et al.^65^, the input and reconstruction layers for the ATAC modality are both split into 22 independent, fully connected layers based on the genomic regions of different human chromosomes (excluding sex chromosomes).

To train MIDAS, we set the modality alignment loss weight (*α*) to 50, the technical IB loss weight (*β*^*s*^) to 30, the biological IB loss weight (*β*^*x*^) to 4 and the technical noise likelihood loss weight (*γ*) to 1,000. The number of updates (*K*) of the batch classifiers $\{r,{\{{r}^{m}\}}_{m\in {{{\mathcal{M}}}}}\}$ in each iteration is set to 3. We split the dataset into training and validation sets in a ratio of 95:5. The minibatch size is set to 256, and we use the AdamW^75^ optimizer with a learning rate of 10^−4^ for implementing SGD. We train the model for a maximum of 2,000 epochs and use early stopping to terminate training. The dropout rates for all hidden layers are set to 0.2. All the hyperparameter settings for MIDAS training are listed in Supplementary Table 14.

### Implementation of comparing methods

We compared MIDAS with 19 recent methods on different trimodal and bimodal integration tasks (see Supplementary Table 3 for an overview). If a method cannot handle missing features for a certain modality, we used the feature intersection of different batches of that modality for integration. For a fair comparison, we set the size of the low-dimensional representations generated by each method to be 32, the same as that of the biological states inferred by MIDAS. For other settings of each method, if not specified, their default values were used. For trimodal rectangular integration tasks, because few methods are directly applicable to ATAC, RNA and ADT trimodal data, we decomposed the rectangular integration into two steps, that is, batch correction for each modality independently and modality fusion for all batch-corrected modalities. We then combined different batch correction and modality fusion methods to achieve rectangular integration, resulting in nine different strategies in total.

#### Methods compared in trimodal rectangular integration tasks

##### BBKNN+average

The Python package BBKNN^76^ (v1.5.1) is used for batch correction (embedding space), and graph averaging is used for modality fusion. For each batch, we use functions from Seurat to perform dimensionality reduction on the count data. We first use RunTFIDF and RunSVD functions to obtain the low-dimensional representation of ATAC data and then use NormalizeData, ScaleData and RunPCA functions to obtain the low-dimensional representations of RNA and ADT data, respectively. For the obtained low-dimensional representation of each modality, we use the bbknn function of the Scanpy^77^ (v1.9.1) Python package to remove the batch effect and obtain a similarity graph. Finally, we average the similarity graphs of different modalities to obtain the output.

##### Harmony+WNN

The R package Harmony^61^ (v0.1.1) is used for batch correction (embedding space), and the WNN algorithm^15^ of the Seurat package is used for modality fusion. We use the same processing method as BBKNN+average to obtain low-dimensional representations of different batches of ATAC, RNA and ADT data, respectively. For the obtained low-dimensional representation of each modality, we use the RunHarmony function of the Harmony package to remove batch effects. We then use Seurat’s FindMultiModalNeighbors function, that is, the WNN algorithm, to fuse the low-dimensional representations of different modalities to obtain the graph output.

##### LIGER+WNN

The R package LIGER^78^ (v1.0.0) is used for batch correction (embedding space), and WNN is used for modality fusion. For each batch, we use Seurat’s RunTFIDF and ScaleData functions for ATAC data normalization and the NormalizeData and ScaleData functions for RNA and ADT data normalization. For each modality, we then use the RunOptimizeALS and RunQuantileNorm functions of the LIGER package for dimensionality reduction and batch effect removal. Finally, we use the WNN algorithm FindMultiModalNeighbors function to fuse the low-dimensional representations of different modalities to obtain the graph output.

##### MOFA+

The R package MOFA+^24^ (v1.4.0) is used for simultaneous batch correction (embedding space) and modality fusion. We first use the same processing method as LIGER+WNN to normalize each modality separately and then use the run_mofa and get_factors functions of the MOFA+ package to achieve simultaneous batch effect removal and modality fusion on the normalized data, obtaining low-dimensional representations output.

##### PCA+WNN

Singular value decomposition is used for the dimensionality reduction of ATAC data, and principal component analysis is used for the dimensionality reduction of RNA and ADT data. No batch correction is applied. WNN is then used for modality fusion. We use the same processing method as BBKNN+average to obtain low-dimensional representations of different batches of ATAC, RNA and ADT data, respectively. We then use the WNN algorithm FindMultiModalNeighbors function to fuse the low-dimensional representations of different modalities to obtain the graph output.

##### Scanorama-embed+WNN

The Python package Scanorama^78^ (v1.7.2) is used for batch correction (embedding space), and WNN is used for modality fusion. For each modality, we use the integrate function from the Scanorama package for dimensionality reduction and batch effect removal. We then use the WNN algorithm FindMultiModalNeighbors function to fuse the low-dimensional representations of different modalities to obtain the graph output.

##### Scanorama-feat+WNN

Scanorama is used for batch correction (feature space), and WNN is used for modality fusion. For each modality, we perform batch correction using the correct function of the Scanorama package. For the batch-corrected count data, we then use the PCA+WNN strategy to get the graph output.

##### Seurat-CCA+WNN

Seurat’s CCA^13^ is used for batch correction (feature space), and WNN is used for modality fusion. For each modality, we use Seurat’s FindIntegrationAnchors function (reduction = ‘cca’), that is, the CCA algorithm, to anchor different batches and use its IntegrateData function to correct batch effects. For the batch-corrected count data, we then use the PCA+WNN strategy to get the graph output.

##### Seurat-RPCA+WNN

Seurat’s RPCA^13^ is used for batch correction (feature space), and WNN is used for modality fusion. It uses the same strategy as Seurat-CCA+WNN, except that the FindIntegrationAnchors function is applied with reduction = ‘rpca’.

#### Methods compared in trimodal mosaic integration tasks

##### Multigrate

The Python package Multigrate^29^ (v0.0.2) is available at https://github.com/theislab/multigrate. For data inputs, we took the intersection of genes in scRNA-seq data and proteins in ADT data across different batches. We processed the data using the default method of Multigrate. The values of parameters KL and integ were set to 0.1 and 3,000, respectively.

##### scMoMaT

The Python package scMoMaT^27^ (v0.2.0) is designed to integrate multimodal mosaic data. The code is available at https://github.com/PeterZZQ/scMoMaT. We take the same preprocessed data as MIDAS. For each modality, because scMoMaT does not handle missing features, we only use the intersected features of different batches of the preprocessed data for integration. We set the minibatch size to 0.1 × *N* for training, where *N* is the number of cells.

##### scVAEIT

The Python package scVAEIT^26^ is designed to integrate multimodal mosaic data. The code is available at https://github.com/jaydu1/scVAEIT. After filtering the low-quality cells and features as MIDAS did, we size normalized and log normalized the counts of genes and proteins separately while binarizing the peaks by changing all nonzero values to 1.

##### StabMap

The R package StabMap^28^ (v0.1.8) is designed to integrate single-cell data with non-overlapping features. The code is available at https://github.com/MarioniLab/StabMap. To select suitable highly variable features, we set different parameters for different modalities (mean > 0.01 and *P* ≤ 0.05 for RNA; mean > 0.25 and *P* ≤ 0.05 for ATAC; mean > 0.01 and *P* ≤ 0.1 for ADT). In the case of diagonal integration, because there are no shared features between different modalities, we convert the ATAC regions into the nearest genes using the ClosestFeature function in Signac and convert the ADT proteins into the corresponding genes. In addition, to obtain more shared features between different modalities in diagonal integration, we relaxed the conditions of highly variable features (mean > 0.01 and *P* ≤ 0.1 for RNA; mean > 0.25 and *P* ≤ 0.05 for ATAC; all features for ADT). In diagonal integration, we choose the RNA batch as the reference set; in other cases, we choose the batch with the largest number of modalities as the reference set.

#### Methods compared in bimodal (ATAC and RNA) integration tasks

##### Cobolt

The Python package Cobolt^21^ (v1.0.1) is designed to integrate bimodal mosaic data from ATAC and RNA data. The code is available at https://github.com/epurdom/cobolt. We take the same preprocessed data as MIDAS and retain the intersected features of each modality for different batches. For Cobolt to read, we store the preprocessed data of each modality in each batch as a SingleData object. We set the learning rate to 5 × 10^−4^.

##### MultiVI

MultiVI^22^ is designed to integrate bimodal mosaic data from ATAC and RNA data. The code is integrated into the Python package scvi-tools (v1.0.0), which is available at https://github.com/scverse/scvi-tools. We take the same preprocessed data as MIDAS. For each modality, we also retain intersected features of different batches. In the model setup, we use batch_key to specify the cell modality and use categorical_covariate_keys to specify the cell batch.

##### uniPort

The Python package uniPort^23^ (v1.2.2) is designed to integrate heterogeneous single-cell bimodal data. The code is available at https://github.com/caokai1073/uniPort. Because uniPort supports horizontal, vertical and diagonal integration, we combine all three integration methods to achieve our tasks.

##### GLUE

The Python package GLUE^25^ (v0.3.2) is designed for integrating unpaired multimodal data (for example, scRNA-seq data, scATAC-seq data and snmC-seq data) using graph-linked unified embeddings. Due to GLUE’s inability to handle trimodal integration with ADT data, we limited the evaluation to bimodal ATAC and RNA integration tasks. The code is available at https://github.com/gao-lab/GLUE, whereas the GTF file we used in the experiments can be obtained from https://ftp.ebi.ac.uk/pub/databases/gencode/Gencode_human/release_43/gencode.v43.annotation.gtf.gz. To remove batch effects, we set use_batch = ‘batch’ in the experiments.

#### Methods compared in bimodal (RNA and ADT) integration tasks

##### totalVI

totalVI^18^ is designed to integrate bimodal mosaic data from RNA and ADT data. The code is integrated into the Python package scvi-tools (v1.0.0). As totalVI does not handle missing genes, we took the intersection of genes in RNA data from different input batches. For the ADT data, the union of proteins from different batches is used.

##### sciPENN

The Python package sciPENN^19^ (v1.0.0) is designed to integrate bimodal data from RNA and ADT data and is available at https://github.com/jlakkis/sciPENN. Because sciPENN cannot handle missing genes, we took the intersection of RNA features and the union of ADT features for different input batches.

### Reporting summary

Further information on research design is available in the Nature Portfolio Reporting Summary linked to this article.

## Online content

Any methods, additional references, Nature Portfolio reporting summaries, source data, extended data, supplementary information, acknowledgements, peer review information; details of author contributions and competing interests; and statements of data and code availability are available at 10.1038/s41587-023-02040-y.

## Supplementary information

Supplementary Information Supplementary Notes 1–4, Figs. 1–26 and Tables 1–14.

Reporting Summary

## Data Availability

All single-cell datasets of human PBMCs and BMMCs used in this paper are publicly available. See Supplementary Table 1 for detailed information.

## Code availability

MIDAS was implemented using the Python (v3.8.8) package PyTorch (v2.0.0) with code available at https://github.com/labomics/midas.
