# Supplementary material for: Mosaic integration and knowledge transfer of single-cell multimodal data with MIDAS
Source: Nat Biotechnol. 2024 Jan 23;42(10):1594–605. doi: 10.1038/s41587-023-02040-y (PMC11471558; doi:10.1038/s41587-023-02040-y)
Supplement: Supplementary file 1 — Supplementary Notes 1–4, Figs. 1–26 and Tables 1–14. [file 41587_2023_2040_MOESM1_ESM.pdf]

nature biotechnology

Article https://doi.org/10.1038/s41587-023-02040-y

Mosaic integration and knowledge transfer of single-cell multimodal data with MIDAS

In the format provided by the authors and unedited

Supplementary Notes

Supplementary Note 1: Evaluation of the robustness, versatility, and efficiency of MIDAS in mosaic integration tasks

Given the several hyperparameters in MIDAS architecture, we systematically evaluated the performance of MIDAS with different settings of each hyperparameter on eight mosaic integration tasks. The results show that the default values of 32 for biological state latent variable dimension (D^c) and 3 for hidden layer number (L) respectively achieve the highest overall scores and ensure compatibility with complex data while maintaining comparable performance (Supplementary Fig. 13). For the other five hyperparameters, MIDAS exhibited considerable robustness to hyperparameter settings across most tasks (Supplementary Fig. 13). However, disabling these five modules always results in a decrease of MIDAS's performance.

In real-world scenario, MIDAS may suffer from biological or technical differences across batches. Thus we conducted evaluations of MIDAS on integrating constructed mosaic datasets with missing cell types or different sequencing depths (Supplementary Tables 5 and 6). MIDAS is slightly affected by missing cell types. Loss of major cell types has a greater impact on MIDAS's performance than the loss of minor cell types (Supplementary Fig. 14a). The UMAP plots indicate that MIDAS can effectively prevent batch overcorrection while aligning cell types from different datasets (Supplementary Fig. 15). When given gradually decreasing different depths, the model performance decreased slightly in most datasets, except for the diagonal+full and diagonal datasets (Supplementary Fig. 14b). However, even when the sequencing depth sampling rate was 0.05, the scMIB score of MIDAS were still maintained above 0.7, except for the diagonal+full and diagonal datasets.

Apart from the 13 trimodal integration methods, we also compared MIDAS against other six methods on bimodal mosaic data (Supplementary Table 3). In all ten bimodal integration tasks (Supplementary Tables 7 and 8), MIDAS outperformed the other methods on both dogma and teadog datasets (Supplementary Figs. 16 and 17).

We evaluated the computing time and memory consumed by MIDAS and 19 other approaches on a Linux server with 36 CPU cores (two Intel Xeon Gold 6240 chips), 512 GB of RAM, and an NVIDIA Tesla V100S GPU. MIDAS is the fastest deep learning method with the least memory consumption for tri-omics integration tasks (Supplementary Fig. 18). As for the bi-omics integration tasks, MIDAS is comparable to other methods.

Supplementary Note 2: Detailed discussion of recent methods for single-cell multimodal integration and knowledge transfer

Recently, several methods for single-cell multimodal integration have been proposed. GLUE [1], designed for the integration of unpaired single-cell trimodal data, aligns the global distributions of different modalities with the use of prior information to improve cell-wise correspondence. It may face challenges when little prior information about the inter-modality (*e.g.*, ATAC *vs.* ADT) correspondence of single cells is available. In addition, it is not designed to utilize paired data to enhance modality alignment. With the widespread adoption and rapid development of scMulti-omics sequencing technologies, paired data are rapidly becoming more common and will soon be ubiquitous. By leveraging paired cell data, MIDAS can learn to better align different modalities in a self-supervised manner and can thus play a more important role than other available methods in the blooming scMulti-omics era.

Most recently, scVAEIT [2], scMoMaT [3], StabMap [4] and Multigrate [5] were proposed for mosaic integration. However, they do not have the functionalities of modality alignment and batch correction. StabMap also requires to manually select paired datasets as reference sets in advance, leading to biased results. A general mosaic integration method should allow input of diverse mosaic combinations, and support modality alignment and batch correction both in embedding space and feature space [6]. These functions are all essential and urgently needed in real-world

scenarios. Compared to scVAEIT, scMoMaT, StabMap and Multigrate, MIDAS is the only one that tackles the problem of general mosaic integration (Supplementary Table 11).

Recently proposed methods for scalable reference-to-query knowledge transfer for single-cell multimodal data have issues with generalization to unseen query data [7, 8] or the retention of information learned on the reference data [9, 10], which make the alignment of reference and query datasets difficult. In addition, they support limited numbers of specific modalities. The MIDAS knowledge transfer scheme stands out from these methods because it supports various types of mosaic query dataset and enables model transfer for sample-efficient mosaic integration and label transfer for automatic cell annotation. Moreover, the generalization and retention problems are mitigated through a novel reciprocal reference mapping scheme.

Supplementary Note 3: Derivation of the variational posterior $q_{\boldsymbol{\phi}}(\boldsymbol{z}|\boldsymbol{x}_n, s_n)$

The detailed derivation of the variational posterior $q_{\boldsymbol{\phi}}(\boldsymbol{z}|\boldsymbol{x}_n, s_n)$ for Eq. 9 is as follow:

$$
\begin{aligned}
p(\boldsymbol{z}|\boldsymbol{x}_n, s_n) &= \frac{p(\boldsymbol{z})p(s_n|\boldsymbol{z})p(\boldsymbol{x}_n|\boldsymbol{z})}{p(\boldsymbol{x}_n, s_n)} \\
&= \frac{p(\boldsymbol{z})}{p(\boldsymbol{x}_n, s_n)} p(s_n|\boldsymbol{z}) \prod_{m\in\mathcal{M}_n} p(\boldsymbol{x}_n^m|\boldsymbol{z}) \\
&= \frac{p(\boldsymbol{z})}{p(\boldsymbol{x}_n, s_n)} \frac{p(s_n)p(\boldsymbol{z}|s_n)}{p(\boldsymbol{z})} \prod_{m\in\mathcal{M}_n} \frac{p(\boldsymbol{x}_n^m)p(\boldsymbol{z}|\boldsymbol{x}_n^m)}{p(\boldsymbol{z})} \\
&= \frac{p(s_n)}{p(\boldsymbol{x}_n, s_n)} \left(\prod_{m\in\mathcal{M}_n} p(\boldsymbol{x}_n^m)\right) p(\boldsymbol{z}) \frac{p(\boldsymbol{z}|s_n)}{p(\boldsymbol{z})} \prod_{m\in\mathcal{M}_n} \frac{p(\boldsymbol{z}|\boldsymbol{x}_n^m)}{p(\boldsymbol{z})} \\
&\approx \frac{p(s_n)}{p(\boldsymbol{x}_n, s_n)} \left(\prod_{m\in\mathcal{M}_n} p(\boldsymbol{x}_n^m)\right) p(\boldsymbol{z}) \frac{q_{\boldsymbol{\phi}}(\boldsymbol{z}|s_n)}{p(\boldsymbol{z})} \prod_{m\in\mathcal{M}_n} \frac{q_{\boldsymbol{\phi}}(\boldsymbol{z}|\boldsymbol{x}_n^m)}{p(\boldsymbol{z})} \\
&\triangleq q_{\boldsymbol{\phi}}(\boldsymbol{z}|\boldsymbol{x}_n, s_n)
\end{aligned}
\tag{37}
$$

where $q_{\boldsymbol{\phi}}(\boldsymbol{z}|s_n)$ and $q_{\boldsymbol{\phi}}(\boldsymbol{z}|\boldsymbol{x}_n^m)$ are the variational approximations of the true posteriors $p(\boldsymbol{z}|s_n)$ and $p(\boldsymbol{z}|\boldsymbol{x}_n^m)$, respectively.

Supplementary Note 4: Derivation of the Information Bottleneck training objective

Here we derive the IB objective of Eq. 23. Starting from the definition of $\beta^s\,\mathrm{I}(s,\boldsymbol{c}) + \beta^x\,\mathrm{I}(\boldsymbol{x},\boldsymbol{u})$ we have:

$$
\begin{aligned}
\beta^s\,\mathrm{I}(s,\boldsymbol{c}) + \beta^x\,\mathrm{I}(\boldsymbol{x},\boldsymbol{u}) &= \beta^s \mathbb{E}_{p(s,\boldsymbol{c})}\left[\log\frac{p(s,\boldsymbol{c})}{p(s)p(\boldsymbol{c})}\right] + \beta^x \mathbb{E}_{p(\boldsymbol{x},\boldsymbol{u})}\left[\log\frac{p(\boldsymbol{x},\boldsymbol{u})}{p(\boldsymbol{x})p(\boldsymbol{u})}\right] \\
&= \beta^s \mathbb{E}_{p(s,\boldsymbol{c})}\big[\log \underbrace{p(s|\boldsymbol{c})}_{\approx p_{\widehat{\boldsymbol{\eta}}}(s|\boldsymbol{c})}\big] \underbrace{-\beta^s \mathbb{E}_{p(s,\boldsymbol{c})}\left[\log p(s)\right]}_{\text{const.}} + \beta^x \mathbb{E}_{p(\boldsymbol{x},\boldsymbol{u})}\left[\log\frac{p(\boldsymbol{u}|\boldsymbol{x})}{p(\boldsymbol{u})}\right] \\
&\approx \beta^s \mathbb{E}_{p(\boldsymbol{x},s)}\left[\mathbb{E}_{p(\boldsymbol{c}|\boldsymbol{x},s)}\left[\log p_{\widehat{\boldsymbol{\eta}}}(s|\boldsymbol{c})\right]\right] + \text{const.} + \beta^x \mathbb{E}_{p(\boldsymbol{x},s)}\left[\mathbb{E}_{p(\boldsymbol{u}|\boldsymbol{x},s)}\left[\log\frac{p(\boldsymbol{u}|\boldsymbol{x})}{p(\boldsymbol{u})}\right]\right] \\
&= \mathbb{E}_{p(\boldsymbol{x},s)}\left[\beta^s \mathbb{E}_{p(\boldsymbol{c}|\boldsymbol{x},s)}\left[\log p_{\widehat{\boldsymbol{\eta}}}(s|\boldsymbol{c})\right] + \beta^x \mathbb{E}_{p(\boldsymbol{u}|\boldsymbol{x},s)}\left[\log\frac{p(\boldsymbol{u}|\boldsymbol{x})}{p(\boldsymbol{u})}\right]\right] + \text{const.} \\
&\approx \frac{1}{N}\sum_n \left[\beta^s \mathbb{E}_{p(\boldsymbol{c}|\boldsymbol{x}_n,s_n)}\left[\log p_{\widehat{\boldsymbol{\eta}}}(s_n|\boldsymbol{c})\right] + \beta^x \mathbb{E}_{p(\boldsymbol{u}|\boldsymbol{x}_n,s_n)}\left[\log\frac{p(\boldsymbol{u}|\boldsymbol{x}_n)}{p(\boldsymbol{u})}\right]\right] + \text{const.} \\
&\approx \frac{1}{N}\sum_n \left[\beta^s \mathbb{E}_{q_{\boldsymbol{\phi}}(\boldsymbol{c}|\boldsymbol{x}_n,s_n)}\left[\log p_{\widehat{\boldsymbol{\eta}}}(s_n|\boldsymbol{c})\right] + \beta^x \mathbb{E}_{q_{\boldsymbol{\phi}}(\boldsymbol{u}|\boldsymbol{x}_n,s_n)}\left[\log\frac{q_{\boldsymbol{\phi}}(\boldsymbol{u}|\boldsymbol{x}_n)}{p(\boldsymbol{u})}\right]\right] + \text{const.}
\end{aligned}
\tag{38}
$$

To calculate $\mathbb{E}_{q_{\boldsymbol{\phi}}(\boldsymbol{u}|\boldsymbol{x}_n,s_n)}\left[\log\frac{q_{\boldsymbol{\phi}}(\boldsymbol{u}|\boldsymbol{x}_n)}{p(\boldsymbol{u})}\right]$, we first define its variational posterior term $q_{\boldsymbol{\phi}}(\boldsymbol{u}|\boldsymbol{x}_n)$ based on the true posterior $p(\boldsymbol{u}|\boldsymbol{x}_n)$:

$$
\begin{aligned}
p(\boldsymbol{u}|\boldsymbol{x}_n) &= p(\boldsymbol{u}|\boldsymbol{x}_n)\underbrace{\frac{p(s_n|\boldsymbol{u},\boldsymbol{x}_n)}{p(s_n|\boldsymbol{u},\boldsymbol{x}_n)}}_{=p(s_n|\boldsymbol{u})}\frac{p(\boldsymbol{x}_n)}{p(\boldsymbol{x}_n)} \\
&= \frac{p(\boldsymbol{u},\boldsymbol{x}_n,s_n)}{p(s_n|\boldsymbol{u})p(\boldsymbol{x}_n)} \\
&= \frac{p(\boldsymbol{u}|\boldsymbol{x}_n,s_n)p(\boldsymbol{x}_n,s_n)}{p(s_n|\boldsymbol{u})p(\boldsymbol{x}_n)} \\
&\approx \frac{q_{\boldsymbol{\phi}}(\boldsymbol{u}|\boldsymbol{x}_n,s_n)p(\boldsymbol{x}_n,s_n)}{p_{\boldsymbol{\theta}}(s_n|\boldsymbol{u})p(\boldsymbol{x}_n)} \\
&\triangleq q_{\boldsymbol{\phi}}(\boldsymbol{u}|\boldsymbol{x}_n)
\end{aligned}
\tag{39}
$$

where $p(s_n|\boldsymbol{u},\boldsymbol{x}_n) = p(s_n|\boldsymbol{u})$ since s and $\boldsymbol{x}$ are conditionally independent given $\boldsymbol{u}$. Substituting $q_{\boldsymbol{\phi}}(\boldsymbol{u}|\boldsymbol{x}_n)$ into the expectation $\mathbb{E}_{q_{\boldsymbol{\phi}}(\boldsymbol{u}|\boldsymbol{x}_n,s_n)}\left[\log\frac{q_{\boldsymbol{\phi}}(\boldsymbol{u}|\boldsymbol{x}_n)}{p(\boldsymbol{u})}\right]$ then yields:

$$
\begin{aligned}
\mathbb{E}_{q_{\boldsymbol{\phi}}(\boldsymbol{u}|\boldsymbol{x}_n,s_n)}\left[\log\frac{q_{\boldsymbol{\phi}}(\boldsymbol{u}|\boldsymbol{x}_n)}{p(\boldsymbol{u})}\right] &= \mathbb{E}_{q_{\boldsymbol{\phi}}(\boldsymbol{u}|\boldsymbol{x}_n,s_n)}\left[\log\frac{q(\boldsymbol{u}|\boldsymbol{x}_n,s_n)p(\boldsymbol{x}_n,s_n)}{p(\boldsymbol{u})p_{\boldsymbol{\theta}}(s_n|\boldsymbol{u})p(\boldsymbol{x}_n)}\right] \\
&= \mathbb{E}_{q_{\boldsymbol{\phi}}(\boldsymbol{u}|\boldsymbol{x}_n,s_n)}\left[\log\frac{q(\boldsymbol{u}|\boldsymbol{x}_n,s_n)}{p(\boldsymbol{u})} - \log p_{\boldsymbol{\theta}}(s_n|\boldsymbol{u}) + \log\frac{p(\boldsymbol{x}_n,s_n)}{p(\boldsymbol{x}_n)}\right] \\
&= \mathrm{KL}\left(q_{\boldsymbol{\phi}}(\boldsymbol{u}|\boldsymbol{x}_n,s_n)\|p(\boldsymbol{u})\right) - \mathbb{E}_{q_{\boldsymbol{\phi}}(\boldsymbol{u}|\boldsymbol{x}_n,s_n)}\left[\log p_{\boldsymbol{\theta}}(s_n|\boldsymbol{u})\right] + \text{const.}
\end{aligned}
\tag{40}
$$

By further substituting Eq. 40 into Eq. 38, we arrive at Eq. 23:

$$
\begin{aligned}
\beta^s \, \mathrm{I}(s, \boldsymbol{c}) + \beta^x \, \mathrm{I}(\boldsymbol{x}, \boldsymbol{u}) \approx & \frac{1}{N} \sum_n \Bigg[\beta^s \mathbb{E}_{q_{\boldsymbol{\phi}}(\boldsymbol{c}|\boldsymbol{x}_n, s_n)} \left[\log p_{\widehat{\boldsymbol{\eta}}}(s_n|\boldsymbol{c}) \right] + \beta^x \, \mathrm{KL} \left(q_{\boldsymbol{\phi}}(\boldsymbol{u}|\boldsymbol{x}_n, s_n) \| p(\boldsymbol{u}) \right) \\
& - \beta^x \mathbb{E}_{q_{\boldsymbol{\phi}}(\boldsymbol{u}|\boldsymbol{x}_n, s_n)} \left[\log p_{\boldsymbol{\theta}}(s_n|\boldsymbol{u}) \right] \Bigg] + \text{const.}
\end{aligned}
$$

where within the summation term on the right-hand side, the first term is approximated by feeding the sample $\boldsymbol{c}_n \sim q_{\boldsymbol{\phi}}(\boldsymbol{c}|\boldsymbol{x}_n, s_n)$ into the classifier r, the second term (the Gaussian KLD) is calculated analytically, and the third term is approximated by feeding the sample $\boldsymbol{u}_n \sim q_{\boldsymbol{\phi}}(\boldsymbol{u}|\boldsymbol{x}_n, s_n)$ into the batch-ID decoder g^s.

References

[1] Zhi-Jie Cao and Ge Gao. Multi-omics single-cell data integration and regulatory inference with graph-linked embedding. *Nature Biotechnology*, pages 1–9, May 2022. ISSN 1546-1696. doi: 10.1038/s41587-022-01284-4.

[2] Jin-Hong Du, Zhanrui Cai, and Kathryn Roeder. Robust probabilistic modeling for single-cell multimodal mosaic integration and imputation via scVAEIT. *Proceedings of the National Academy of Sciences*, 119(49): e2214414119, December 2022. doi: 10.1073/pnas.2214414119.

[3] Ziqi Zhang, Haoran Sun, Ragunathan Mariappan, Xi Chen, Xinyu Chen, Mika S. Jain, Mirjana Efremova, Sarah A. Teichmann, Vaibhav Rajan, and Xiuwei Zhang. scMoMaT jointly performs single cell mosaic integration and multi-modal bio-marker detection. *Nature Communications*, 14(1):384, January 2023. ISSN 2041-1723. doi: 10.1038/s41467-023-36066-2.

[4] Shila Ghazanfar, Carolina Guibentif, and John C. Marioni. Stabilized mosaic single-cell data integration using unshared features. *Nature Biotechnology*, pages 1–9, May 2023. ISSN 1546-1696. doi: 10.1038/s41587-023-01766-z.

[5] Mohammad Lotfollahi, Anastasia Litinetskaya, and Fabian J. Theis. Multigrate: Single-cell multi-omic data integration, March 2022.

[6] Ricard Argelaguet, Anna S. E. Cuomo, Oliver Stegle, and John C. Marioni. Computational principles and challenges in single-cell data integration. *Nature Biotechnology*, pages 1–14, May 2021. ISSN 1546-1696. doi: 10.1038/s41587-021-00895-7.

[7] Lei Xiong, Kang Tian, Yuzhe Li, Weixi Ning, Xin Gao, and Qiangfeng Cliff Zhang. Online single-cell data integration through projecting heterogeneous datasets into a common cell-embedding space. *Nature Communications*, 13(1):6118, October 2022. ISSN 2041-1723. doi: 10.1038/s41467-022-33758-z.

[8] Meng Yang, Yueyuxiao Yang, Chenxi Xie, Ming Ni, Jian Liu, Huanming Yang, Feng Mu, and Jian Wang. Contrastive learning enables rapid mapping to multimodal single-cell atlas of multimillion scale. *Nature Machine Intelligence*, pages 1–14, August 2022. ISSN 2522-5839. doi: 10.1038/s42256-022-00518-z.

[9] Mohammad Lotfollahi, Mohsen Naghipourfar, Malte D. Luecken, Matin Khajavi, Maren Büttner, Marco Wagenstetter, Žiga Avsec, Adam Gayoso, Nir Yosef, Marta Interlandi, Sergei Rybakov, Alexander V. Misharin, and Fabian J. Theis. Mapping single-cell data to reference atlases by transfer learning. *Nature Biotechnology*, pages 1–10, August 2021. ISSN 1546-1696. doi: 10.1038/s41587-021-01001-7.

[10] Fan Yang, Wenchuan Wang, Fang Wang, Yuan Fang, Duyu Tang, Junzhou Huang, Hui Lu, and Jianhua Yao. scBERT as a large-scale pretrained deep language model for cell type annotation of single-cell RNA-seq data. *Nature Machine Intelligence*, pages 1–15, September 2022. ISSN 2522-5839. doi: 10.1038/s42256-022-00534-z.

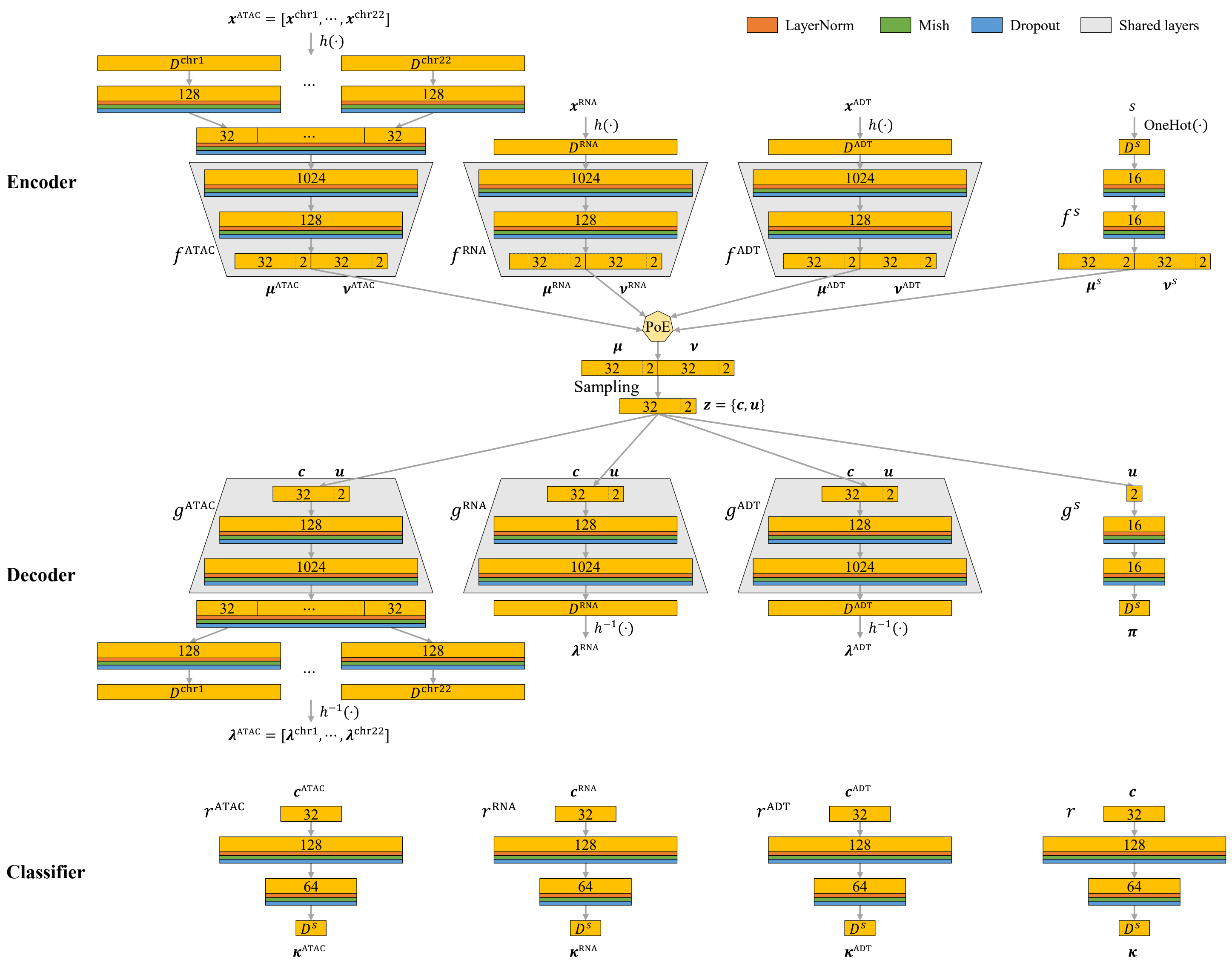

Supplementary Figure 1. Neural network architecture for MIDAS. For cell n, where the measured modalities are represented by $\mathcal{M}_n \subseteq \{\mathrm{ATAC}, \mathrm{RNA}, \mathrm{ADT}\}$, the counts $\boldsymbol{x} = \{\boldsymbol{x}^m\}_{m\in\mathcal{M}_n}$ and batch-ID s are inputted into the corresponding encoders $\{f^m\}_{m\in\mathcal{M}_n}$ and f^s. These encoders generate distribution parameters $\{\boldsymbol{\mu}^m, \boldsymbol{\nu}^m\}_{m\in\mathcal{M}_n}$ and $\{\boldsymbol{\mu}^s, \boldsymbol{\nu}^s\}$, which are then transformed into the distribution parameters $\{\boldsymbol{\mu}, \boldsymbol{\nu}\}$ (representing Gaussian mean and variance) of the latent posterior through PoE. Subsequently, a sample $\boldsymbol{z}$, consisting of the biological state $\boldsymbol{c}$ and technical noise $\boldsymbol{u}$, is drawn from the latent posterior and fed into the modality decoders $\{g^m\}_{m\in\mathcal{M}_n}$ to predict the likelihood distribution parameters $\{\boldsymbol{\lambda}^m\}_{m\in\mathcal{M}_n}$. Additionally, $\boldsymbol{u}$ is inputted into the batch-ID decoder g^s to predict the likelihood distribution parameter $\boldsymbol{\pi}$. The unimodal biological states $\{\boldsymbol{c}^m\}_{m\in\mathcal{M}_n}$, which are generated from the self-supervised modality alignment tasks, along with $\boldsymbol{c}$, are fed into the classifiers $\{r^m\}_{m\in\mathcal{M}_n}$ and r to predict the likelihood distribution parameters $\{\boldsymbol{\kappa}^m\}_{m\in\mathcal{M}_n}$ and $\boldsymbol{\kappa}$. h is the zero-padding function converting $\boldsymbol{x}^m$ of variable size into a fixed size vector, h^{-1} is the masking function used to remove the padded features, and OneHot is the one-hot encoding function. Each hidden layer is followed by three PyTorch modules: LayerNorm, Mish, and Dropout. The sizes (number of dimensions) are marked on each layer (yellow), where the values of $D^{\mathrm{ATAC}}, D^{\mathrm{RNA}}, D^{\mathrm{ADT}}$, and D^s depend on the task. To effectively reduce the number of learnable parameters in the model, the input and reconstruction layers for $\boldsymbol{x}^{\mathrm{ATAC}}$ are both split into 22 independent fully-connected layers based on the genomic regions of different human chromosomes (excluding the sex chromosomes).

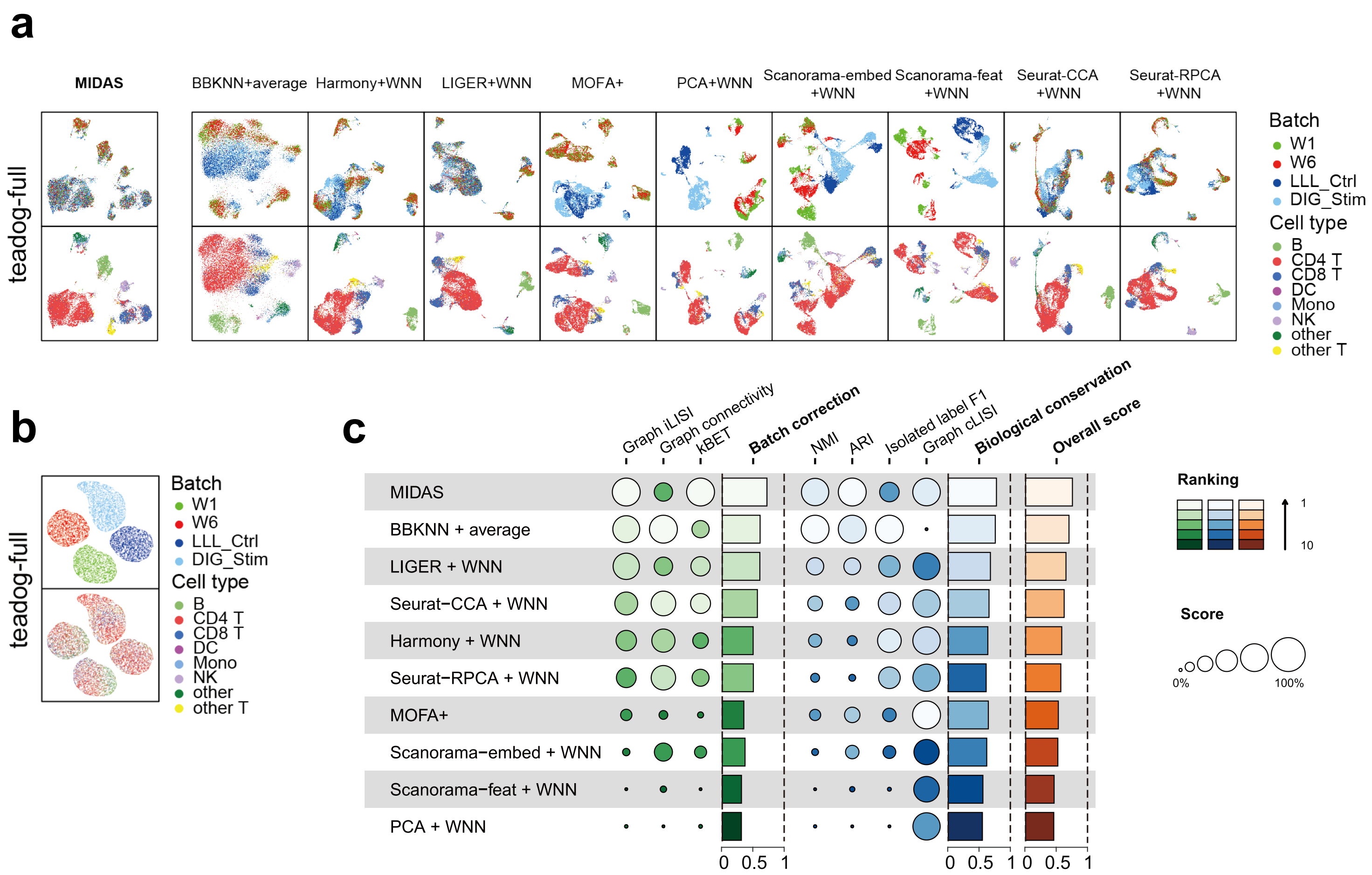

Supplementary Figure 2. Performance of MIDAS on teadog-full dataset. a, UMAP visualization of cell embeddings obtained by MIDAS and nine other strategies in the teadog-full datasets. The left two panels show inferred latent biological states and the right panels show dimensionality reduction results obtained with the other strategies. **b**, UMAP visualization of latent technical noise inferred by MIDAS in the teadog-full datasets. **c**, scIB benchmarking of performance on the teadog-full rectangular integration task.

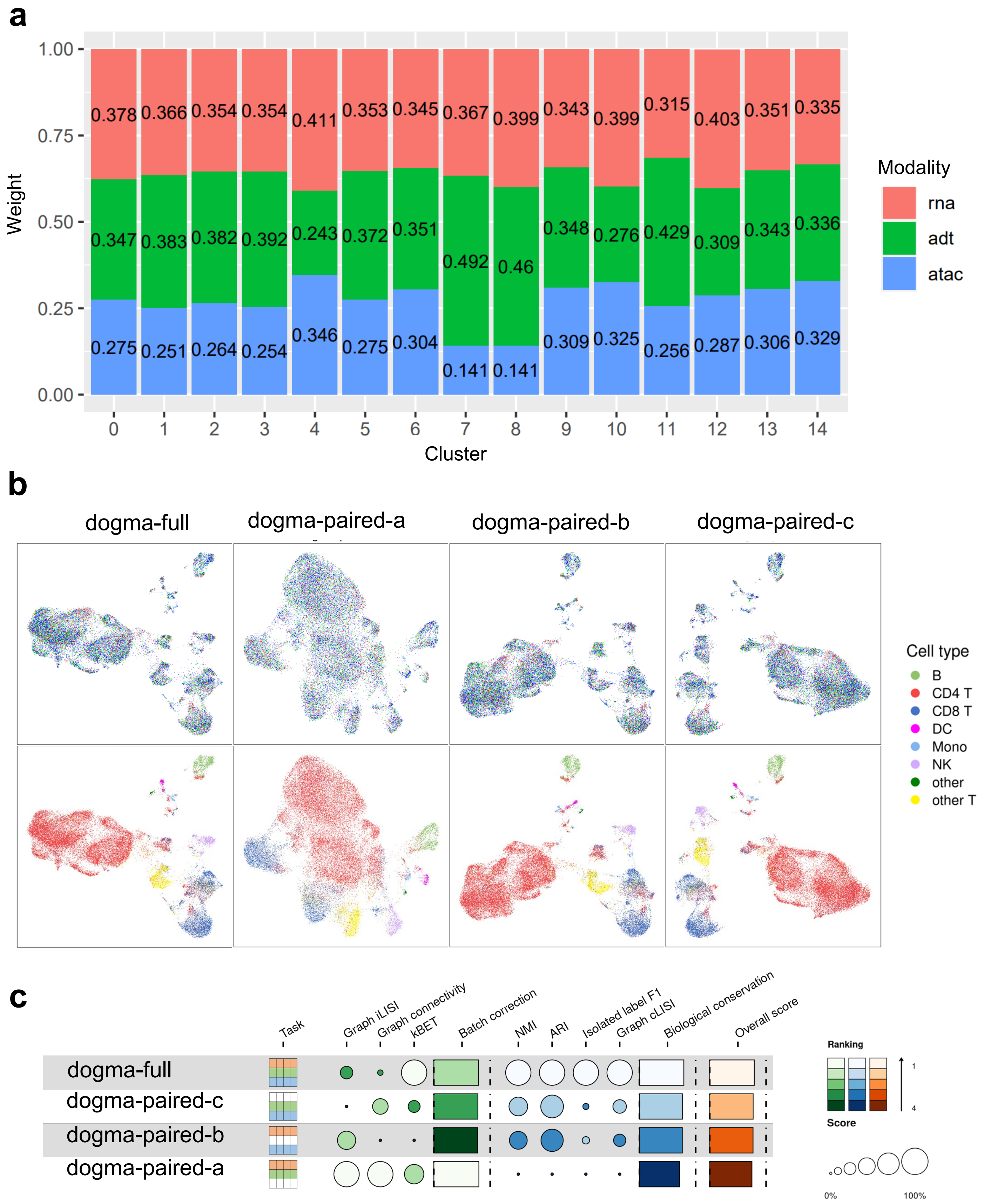

Supplementary Figure 3. The contribution of different modalities to the joint clustering. **a**, Contributions of RNA, ADT and ATAC to each cluster of DOGMA-full dataset. **b**, UMAP comparison of MIDAS embeddings on dogma-full, dogma-paired-a (remove ATAC modality), dogma-paired-b (remove RNA modality), and dogma-paired-c (remove ADT modality) integration tasks. In each task, cells in the top row are colored by batch, and cells in the bottom row are colored by cell type. **c**, scIB benchmarking of MIDAS performance on four tasks in (**b**). The removal of a modality significantly affects biological preservation, with the removal of ADT having the greatest impact.

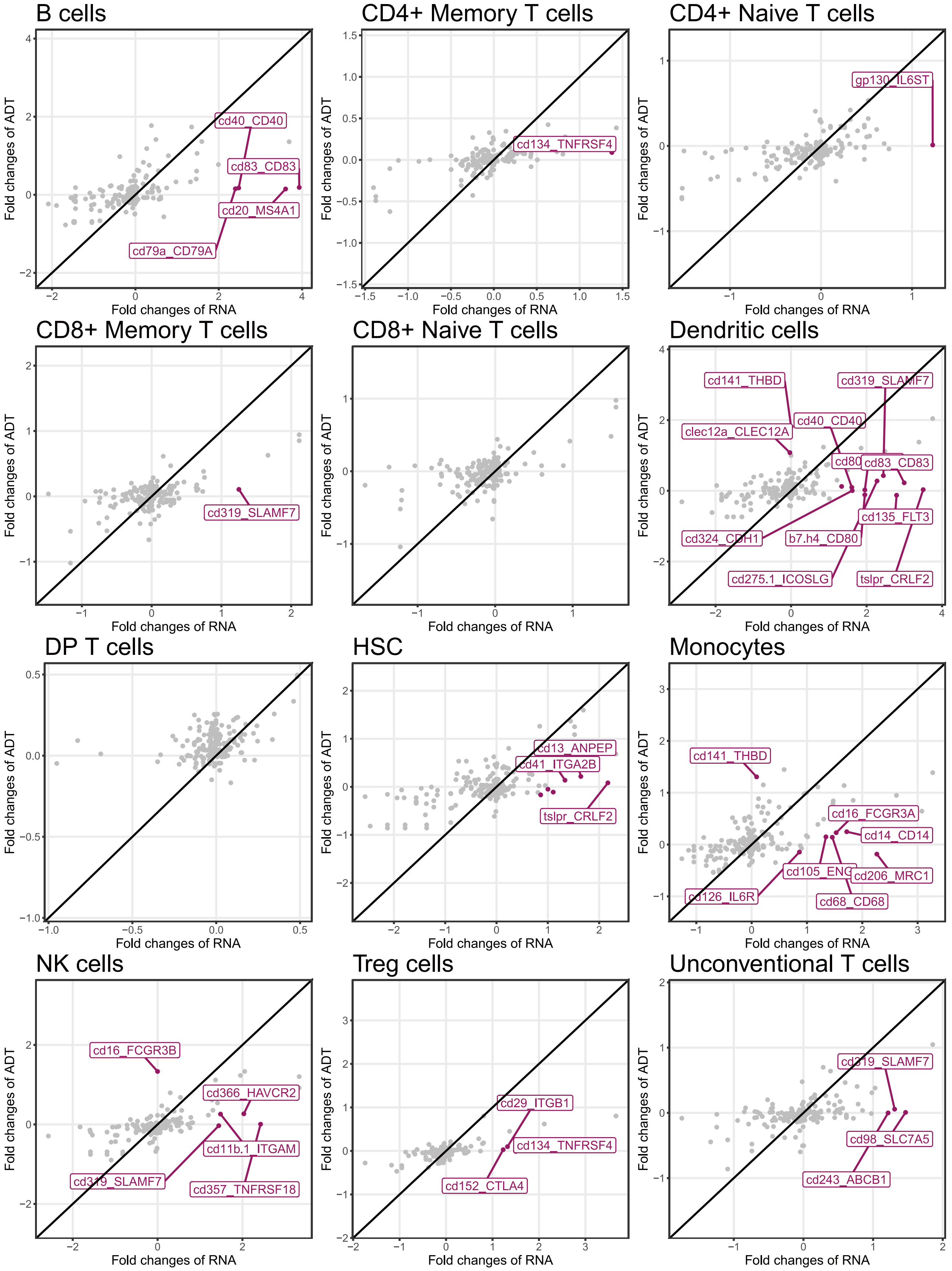

Supplementary Figure 4. Expression inconsistencies between proteins and their corresponding genes in all cell types.

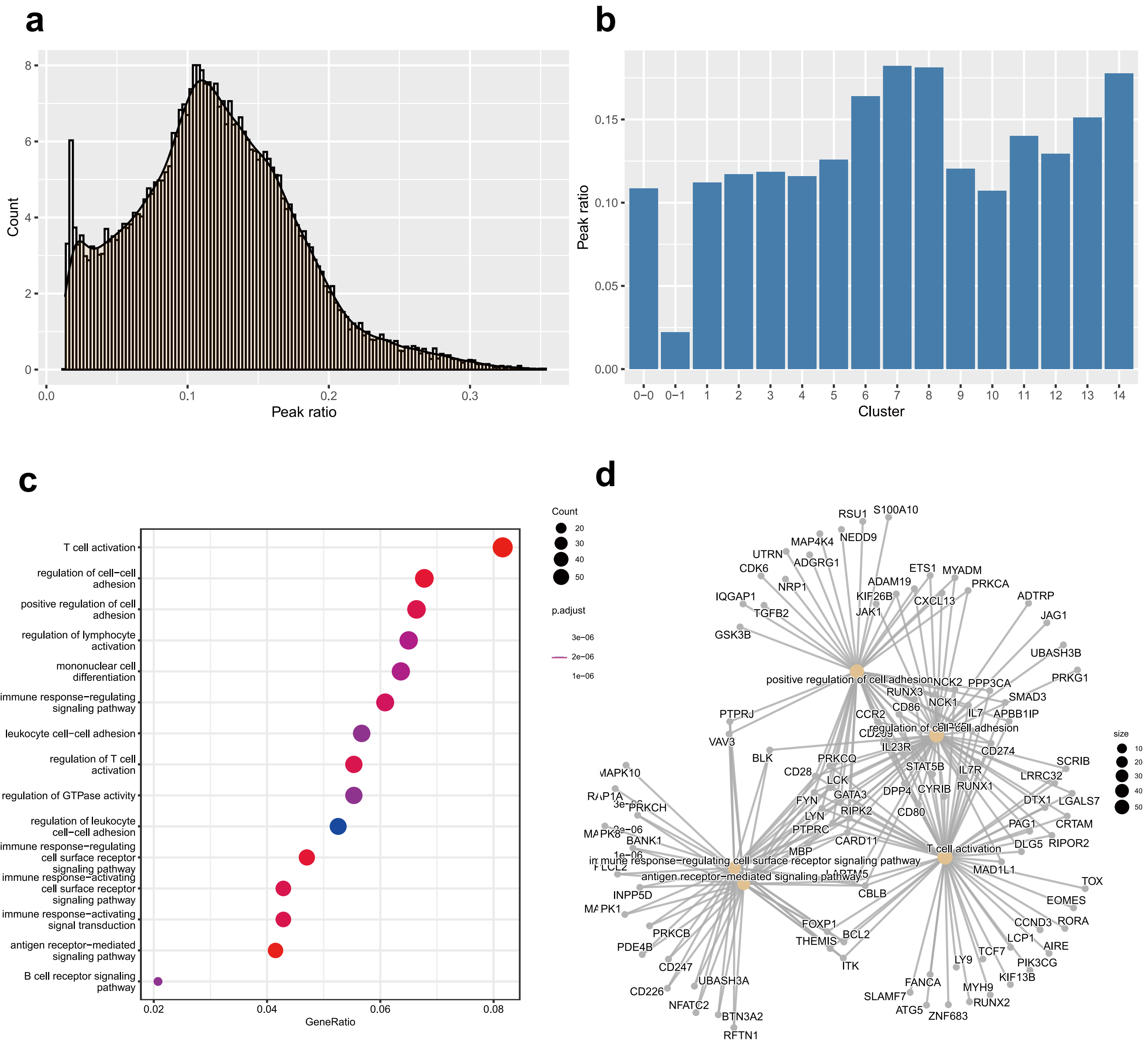

Supplementary Figure 5. Cells with low chromatin accessibility. a, ATAC peak ratio of all cells show a bi-modal distribution; Peak ratio of a cell is defined as the ratio of peak numbers of the cell to that of all the cells. **b**, ATAC peak ratio of cells in clusters. **c**, The Gene Ontology analysis for genes with deficient peak coverage by clusterProfiler (One-sided Fisher's exact test, p-value was adjusted by Benjamini & Hochberg method). **d**, Linkages of genes with dificient peak coverage and biological concepts.

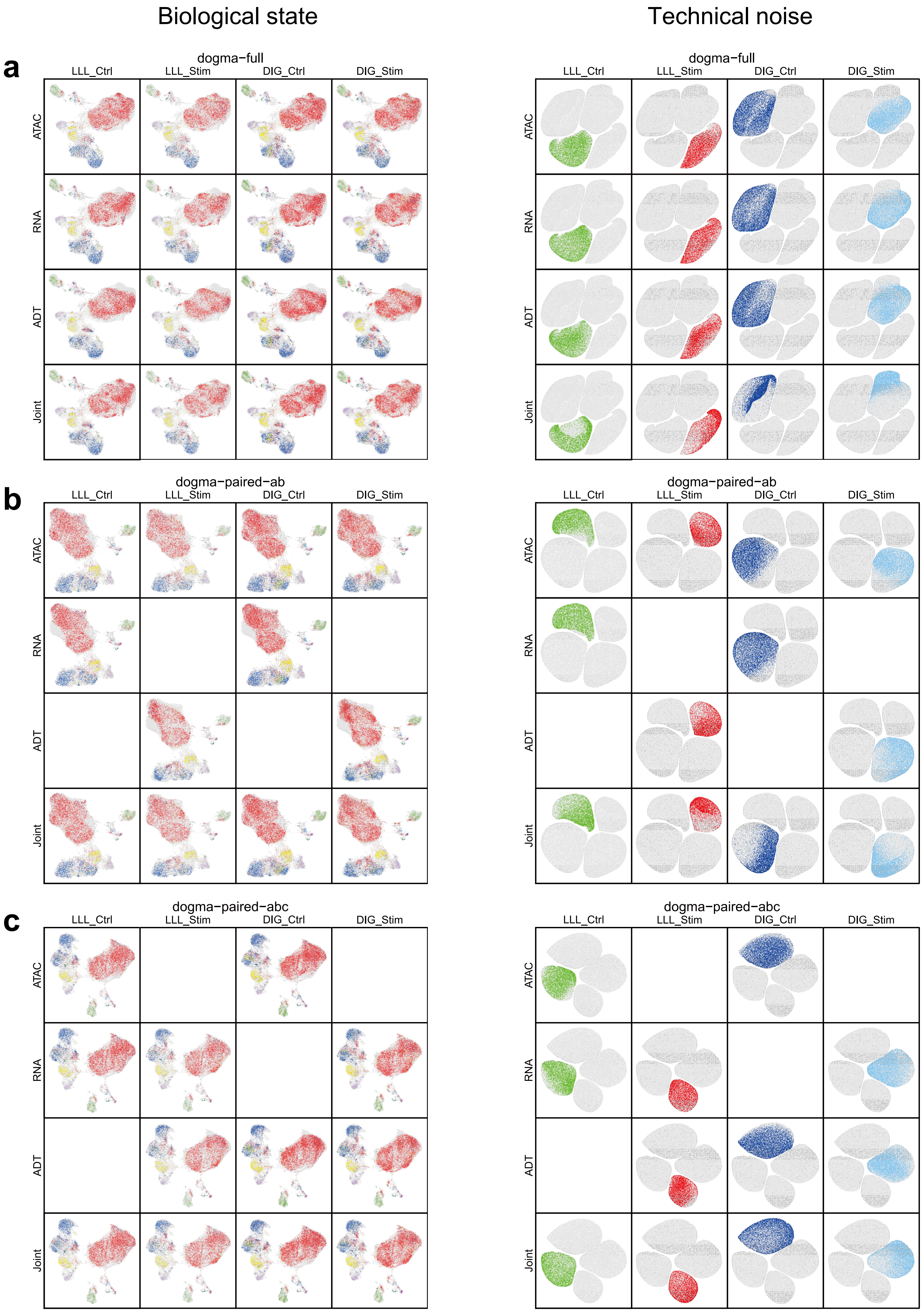
Biological state
Technical noise
a
dogma−full
LLL_Ctrl
LLL_Stim
DIG_Ctrl
DIG_Stim
ATAC
RNA
ADT
Joint
b
dogma−paired−ab
c
dogma−paired−abc

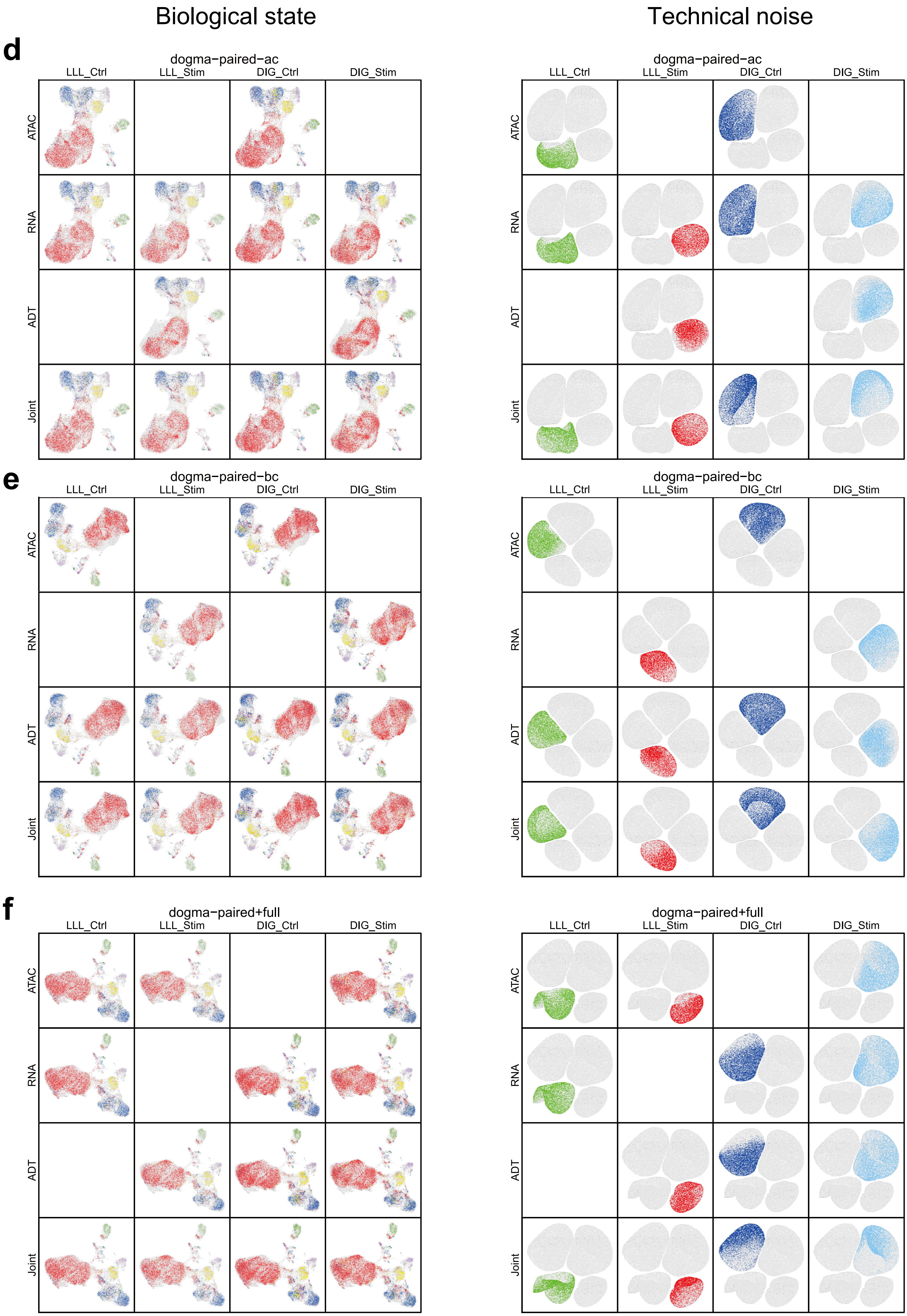

Biological state
Technical noise
d
dogma−paired−ac
e
dogma−paired−bc
f
dogma−paired+full
LLL_Ctrl
LLL_Stim
DIG_Ctrl
DIG_Stim
ATAC
RNA
ADT
Joint

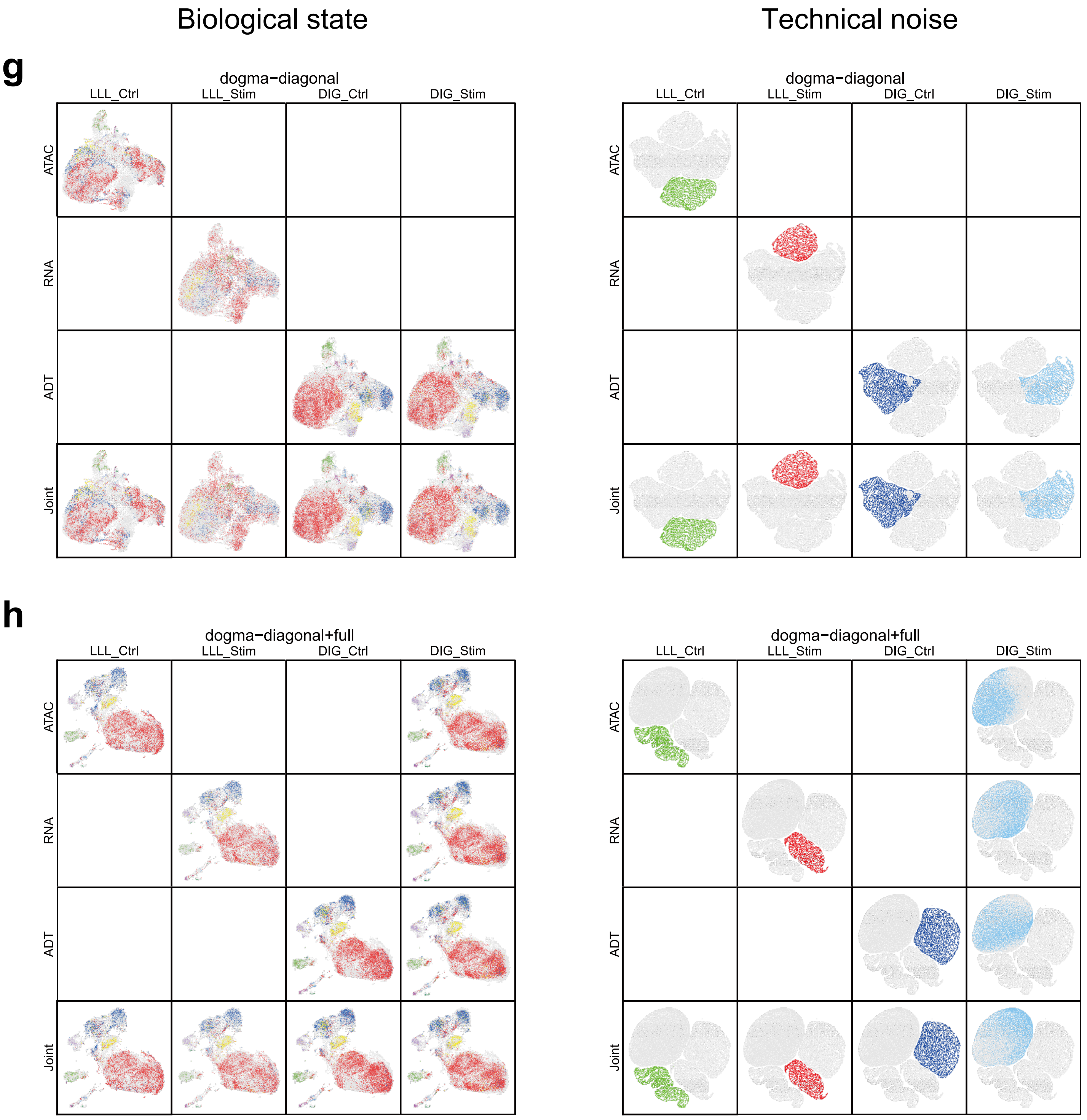

Supplementary Figure 6. UMAP visualization of the biological states (left, colored by cell type) and technical noise (right, colored by batch) inferred by MIDAS with eight dogma mosaic datasets (a–h).

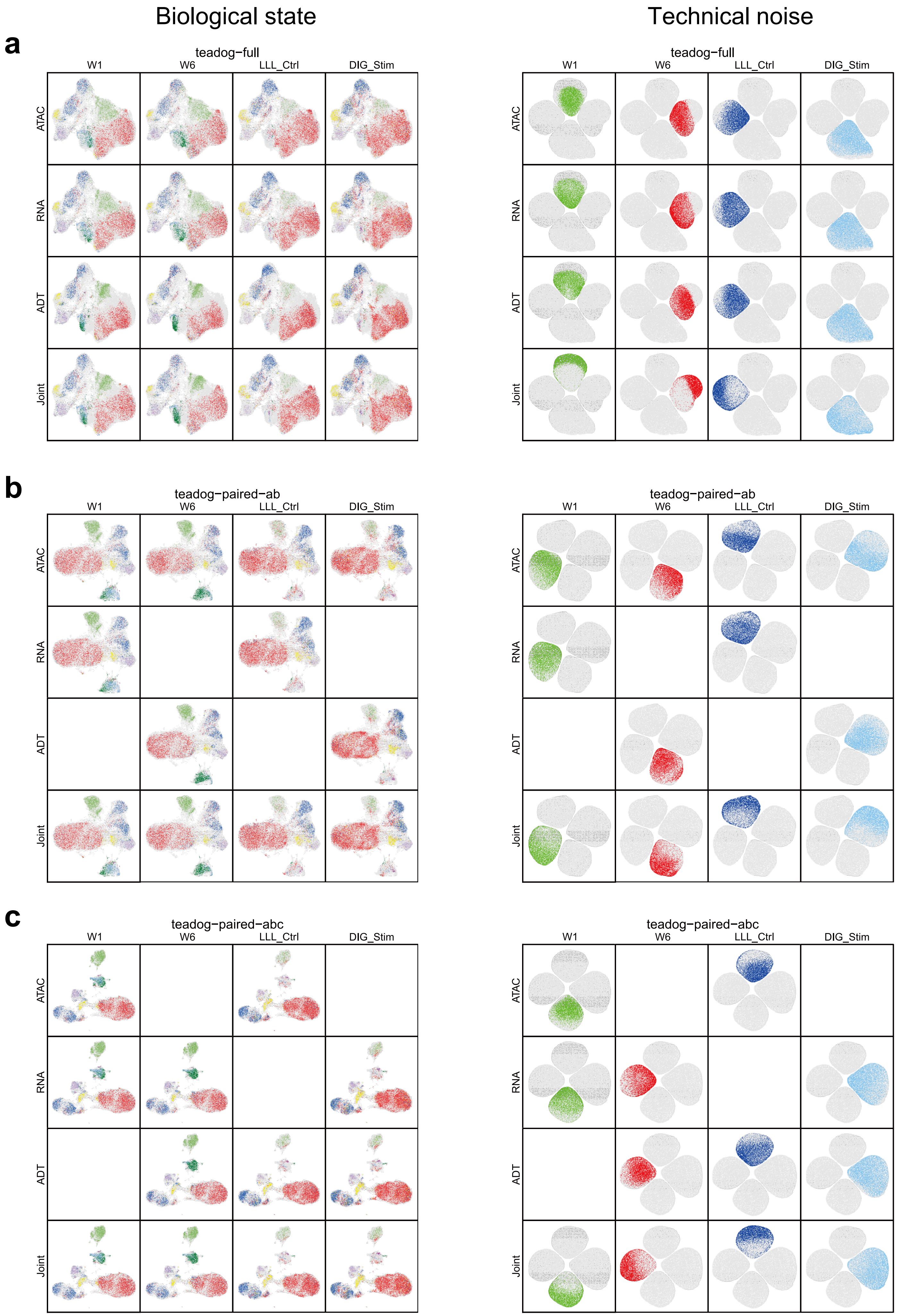

Biological state
Technical noise
a
teadog−full
W1
W6
LLL_Ctrl
DIG_Stim
ATAC
RNA
ADT
Joint
b
teadog−paired−ab
c
teadog−paired−abc

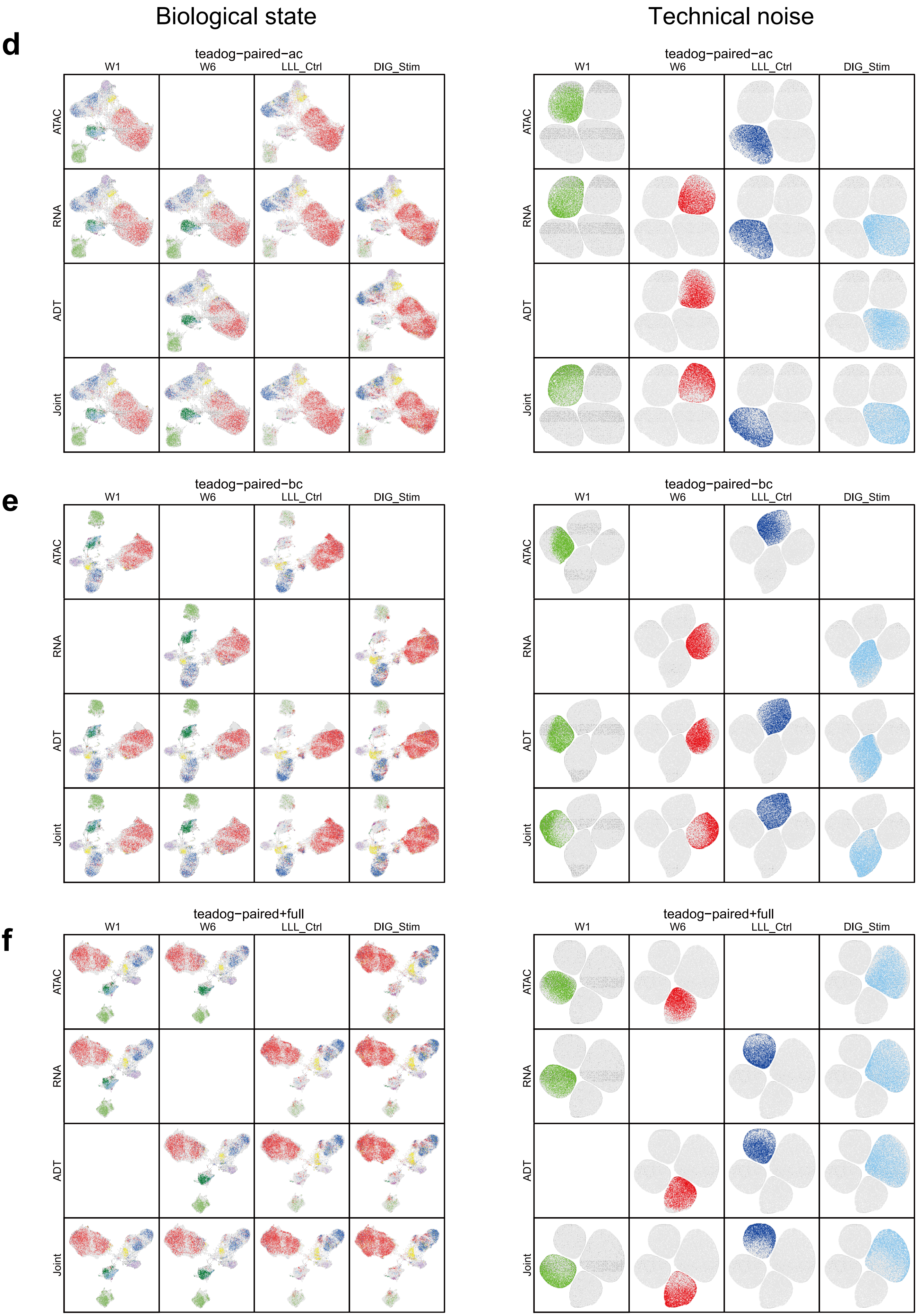

Biological state
Technical noise
d
teadog−paired−ac
W1
W6
LLL_Ctrl
DIG_Stim
ATAC
RNA
ADT
Joint
teadog−paired−ac
W1
W6
LLL_Ctrl
DIG_Stim
ATAC
RNA
ADT
Joint
e
teadog−paired−bc
W1
W6
LLL_Ctrl
DIG_Stim
ATAC
RNA
ADT
Joint
teadog−paired−bc
W1
W6
LLL_Ctrl
DIG_Stim
ATAC
RNA
ADT
Joint
f
teadog−paired+full
W1
W6
LLL_Ctrl
DIG_Stim
ATAC
RNA
ADT
Joint
teadog−paired+full
W1
W6
LLL_Ctrl
DIG_Stim
ATAC
RNA
ADT
Joint

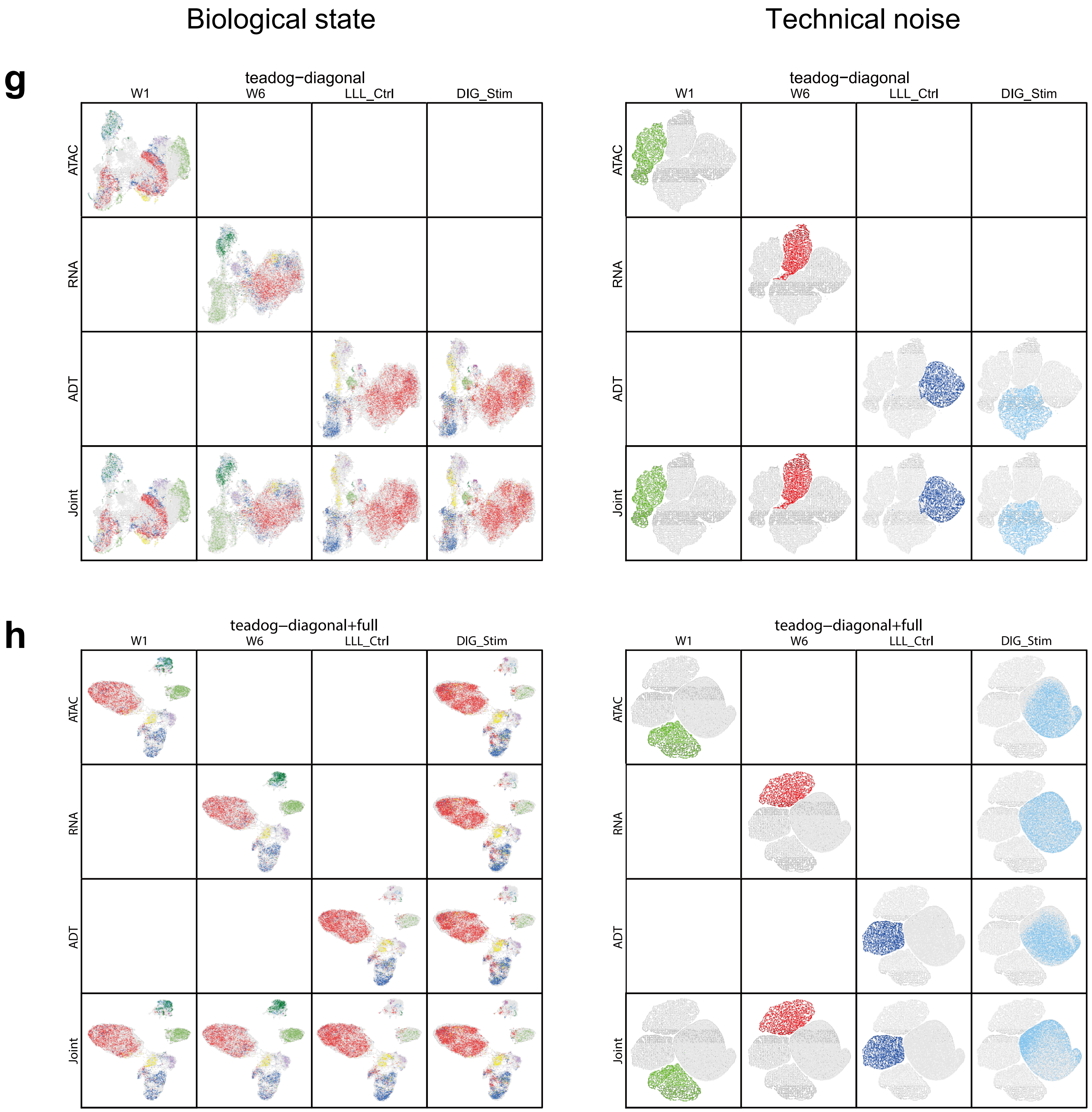

Supplementary Figure 7. UMAP visualization of the biological states (left, colored by cell type) and technical noise (right, colored by batch) inferred by MIDAS with eight teadog mosaic datasets (a–h).

a

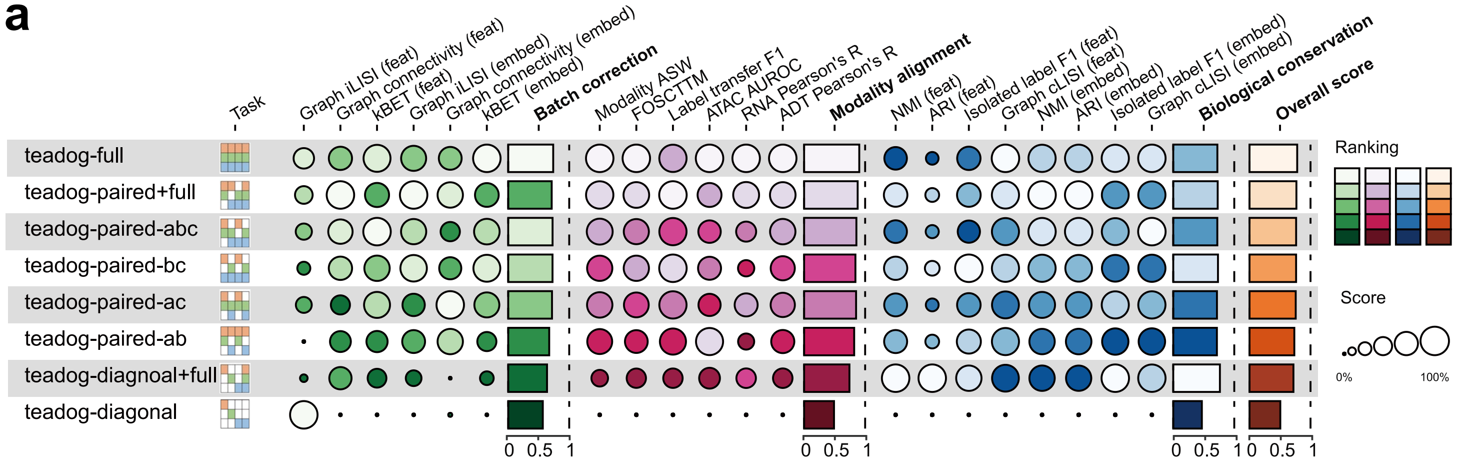
Task
Graph iLISI (feat)
Graph connectivity (feat)
kBET (feat)
Graph iLISI (embed)
Graph connectivity (embed)
kBET (embed)
Batch correction
Modality ASW
FOSCTTM
Label transfer F1
ATAC AUROC
RNA Pearson's R
ADT Pearson's R
Modality alignment
NMI (feat)
ARI (feat)
Isolated label F1 (feat)
Graph cLISI (feat)
NMI (embed)
ARI (embed)
Isolated label F1 (embed)
Graph cLISI (embed)
Biological conservation
Overall score
teadog-full
teadog-paired+full
teadog-paired-abc
teadog-paired-bc
teadog-paired-ac
teadog-paired-ab
teadog-diagnoal+full
teadog-diagonal
0 0.5 1
Ranking
Score
0%
100%

b

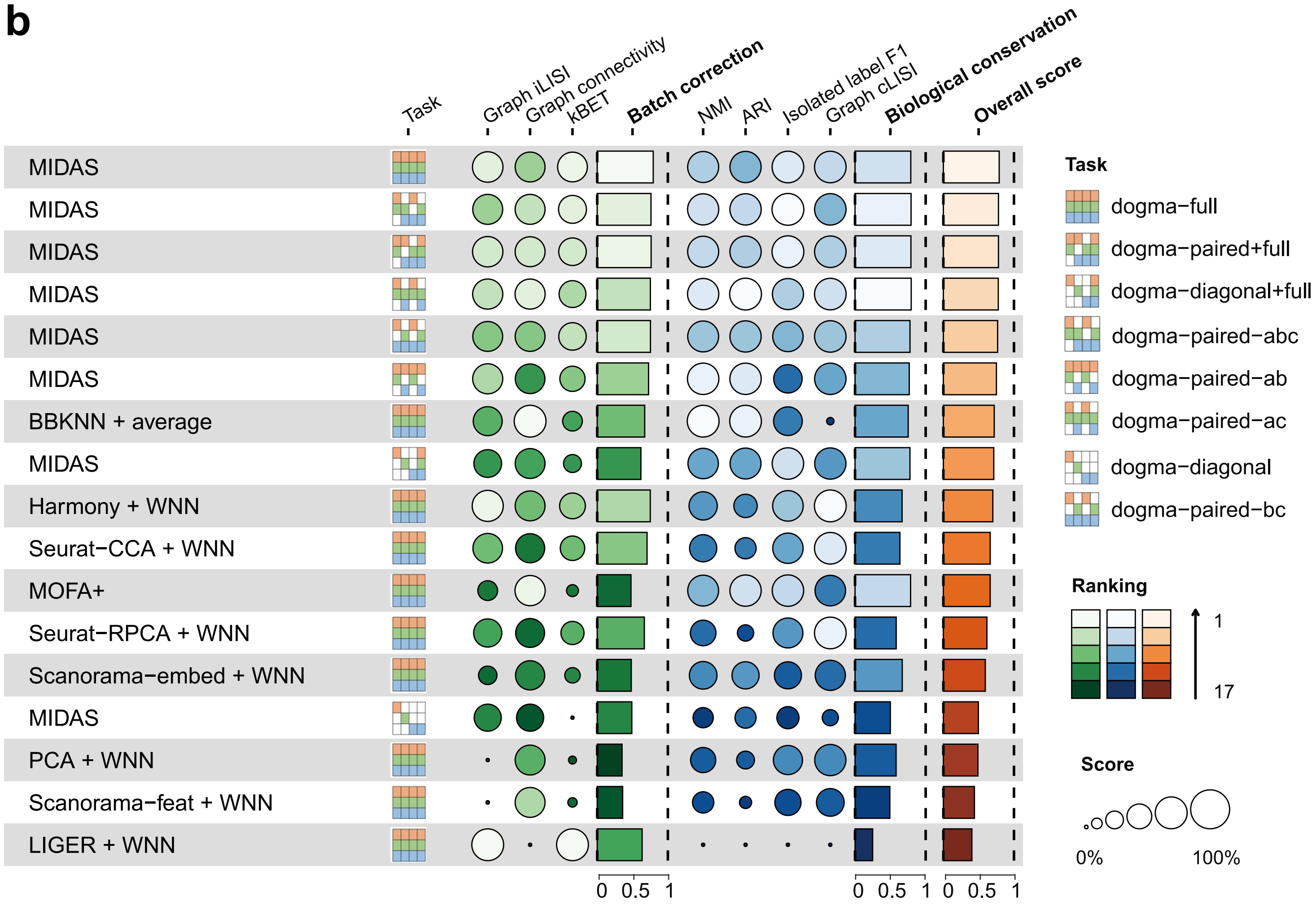
Task
Graph iLISI
Graph connectivity
kBET
Batch correction
NMI
ARI
Isolated label F1
Graph cLISI
Biological conservation
Overall score
MIDAS
MIDAS
MIDAS
MIDAS
MIDAS
MIDAS
BBKNN + average
MIDAS
Harmony + WNN
Seurat−CCA + WNN
MOFA+
Seurat−RPCA + WNN
Scanorama−embed + WNN
MIDAS
PCA + WNN
Scanorama−feat + WNN
LIGER + WNN
0 0.5 1
dogma−full
dogma−paired+full
dogma−diagonal+full
dogma−paired−abc
dogma−paired−ab
dogma−paired−ac
dogma−diagonal
dogma−paired−bc
Ranking
1
17
Score
0%
100%

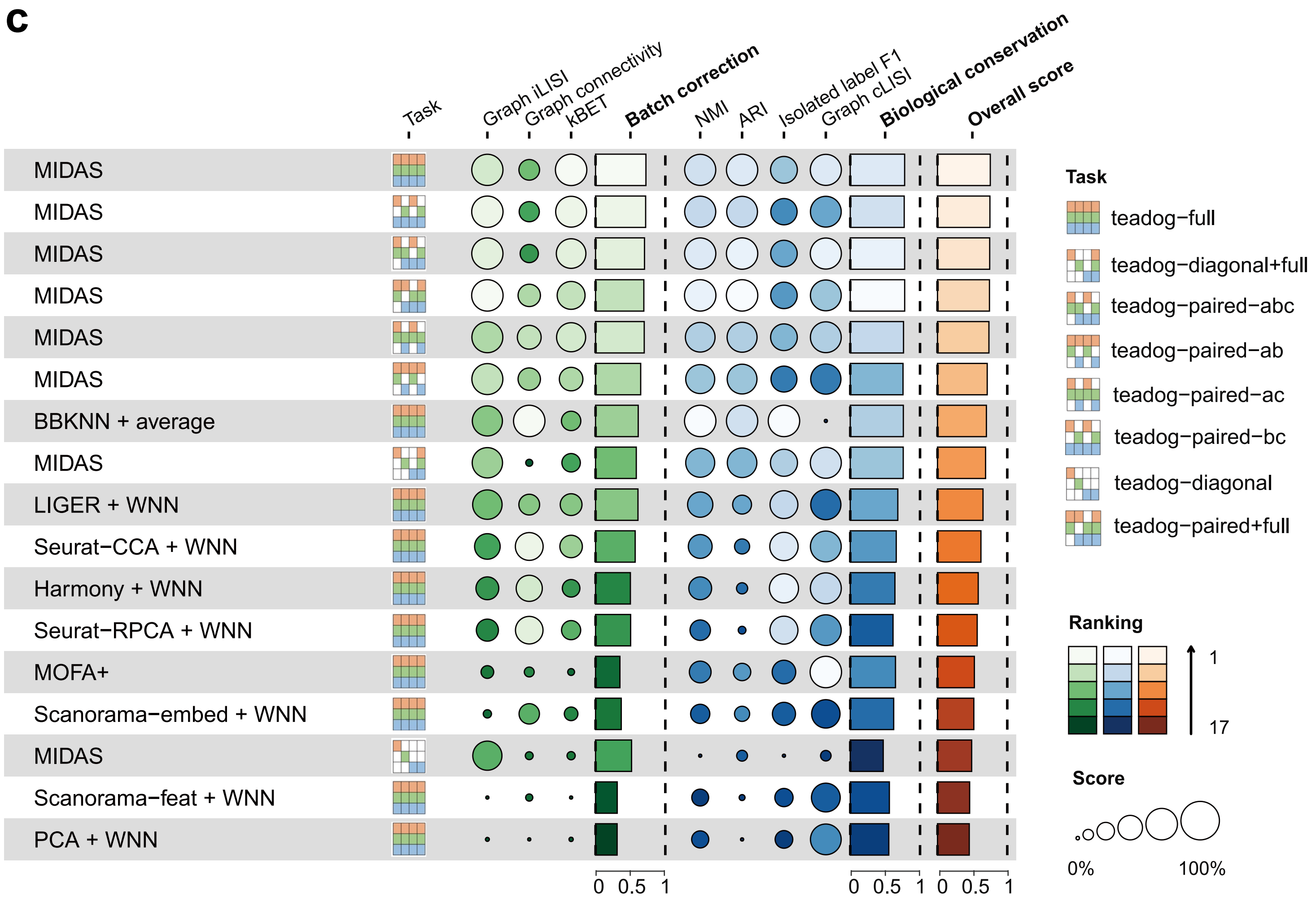

Supplementary Figure 8. scIB and scMIB benchmarking of the performance of MIDAS and other strategies. a, Benchmarking of MIDAS performance on teadog mosaic integration tasks using our proposed scMIB. **b**, scIB benchmarking of MIDAS performance on dogma mosaic integration tasks against nine other strategies on the dogma-full rectangular integration task. **c**, scIB benchmarking of MIDAS performance on teadog mosaic integration tasks against nine other strategies on the teadog-full rectangular integration task.

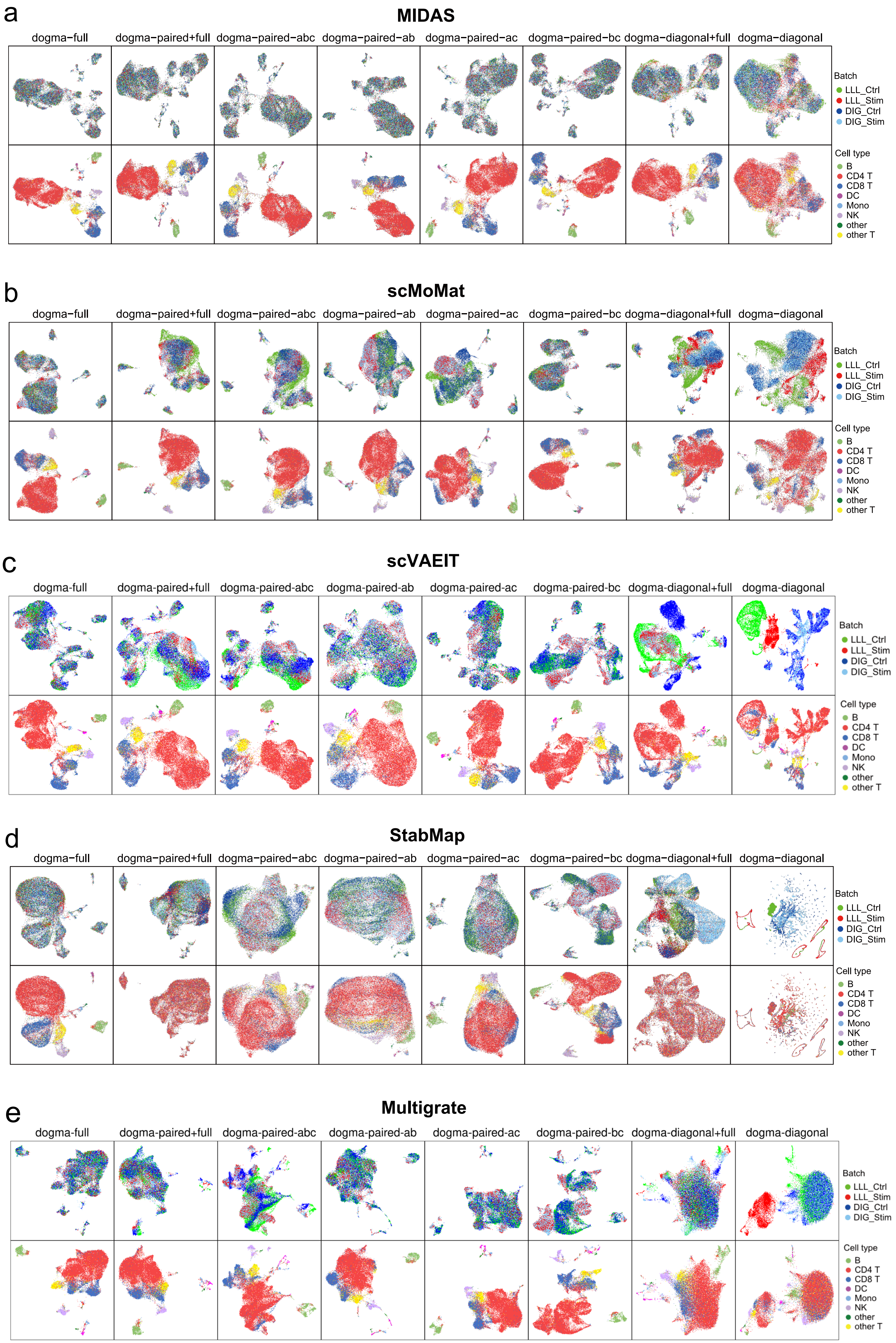

a
MIDAS
dogma−full
dogma−paired+full
dogma−paired−abc
dogma−paired−ab
dogma−paired−ac
dogma−paired−bc
dogma−diagonal+full
dogma−diagonal
Batch
LLL_Ctrl
LLL_Stim
DIG_Ctrl
DIG_Stim
Cell type
B
CD4 T
CD8 T
DC
Mono
NK
other
other T
b
scMoMat
dogma−full
dogma−paired+full
dogma−paired−abc
dogma−paired−ab
dogma−paired−ac
dogma−paired−bc
dogma−diagonal+full
dogma−diagonal
Batch
LLL_Ctrl
LLL_Stim
DIG_Ctrl
DIG_Stim
Cell type
B
CD4 T
CD8 T
DC
Mono
NK
other
other T
c
scVAEIT
dogma−full
dogma-paired+full
dogma-paired-abc
dogma-paired-ab
dogma-paired-ac
dogma-paired-bc
dogma-diagonal+full
dogma-diagonal
Batch
LLL_Ctrl
LLL_Stim
DIG_Ctrl
DIG_Stim
Cell type
B
CD4 T
CD8 T
DC
Mono
NK
other
other T
d
StabMap
dogma−full
dogma−paired+full
dogma−paired−abc
dogma−paired−ab
dogma−paired−ac
dogma−paired−bc
dogma−diagonal+full
dogma−diagonal
Batch
LLL_Ctrl
LLL_Stim
DIG_Ctrl
DIG_Stim
Cell type
B
CD4 T
CD8 T
DC
Mono
NK
other
other T
e
Multigrate
dogma-full
dogma-paired+full
dogma-paired-abc
dogma-paired-ab
dogma-paired-ac
dogma-paired-bc
dogma-diagonal+full
dogma-diagonal
Batch
LLL_Ctrl
LLL_Stim
DIG_Ctrl
DIG_Stim
Cell type
B
CD4 T
CD8 T
DC
Mono
NK
other
other T

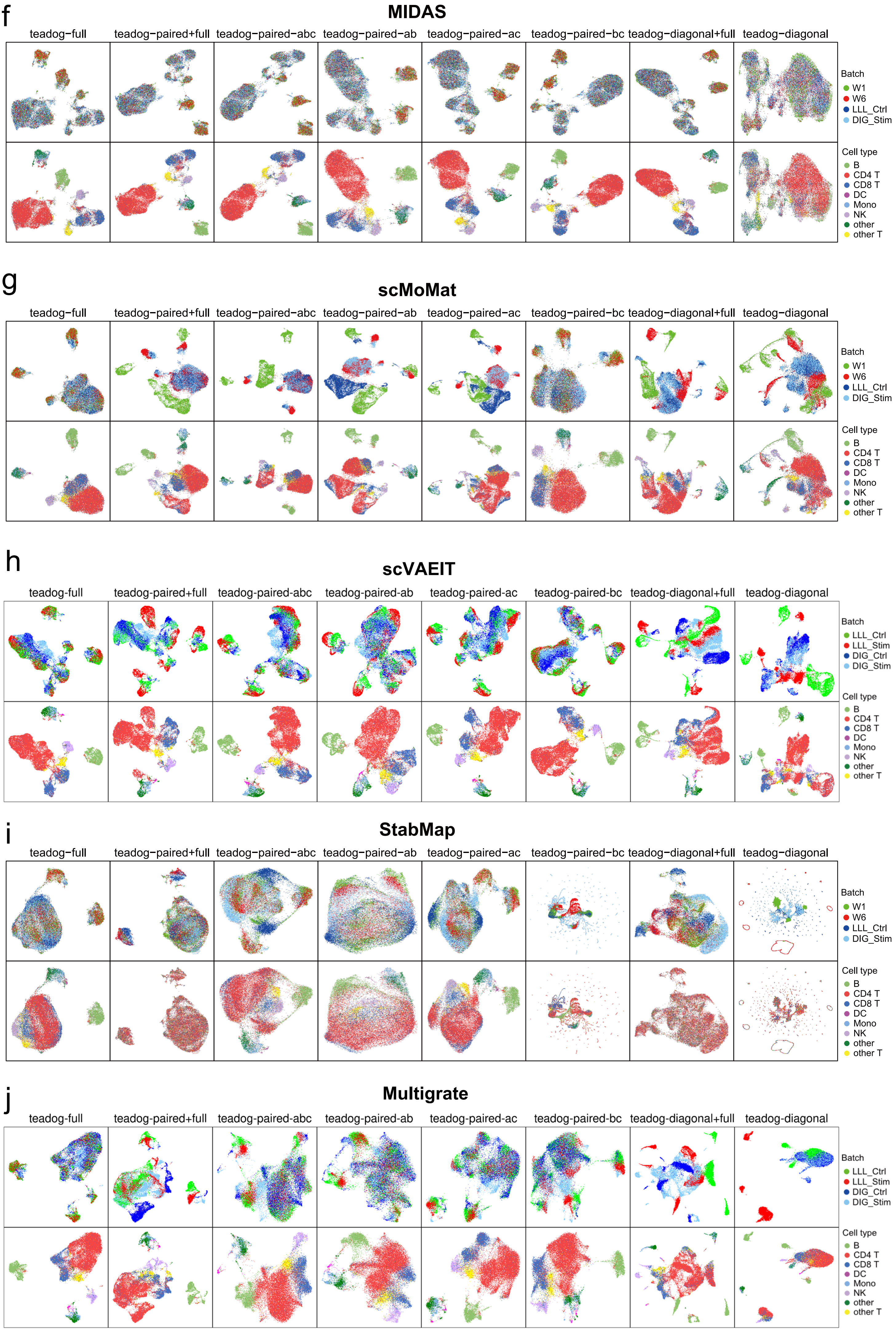

f
MIDAS
teadog−full
teadog−paired+full
teadog−paired−abc
teadog−paired−ab
teadog−paired−ac
teadog−paired−bc
teadog−diagonal+full
teadog−diagonal
Batch
W1
W6
LLL_Ctrl
DIG_Stim
Cell type
B
CD4 T
CD8 T
DC
Mono
NK
other
other T
g
scMoMat
teadog−full
teadog−paired+full
teadog−paired−abc
teadog−paired−ab
teadog−paired−ac
teadog−paired−bc
teadog−diagonal+full
teadog−diagonal
Batch
W1
W6
LLL_Ctrl
DIG_Stim
Cell type
B
CD4 T
CD8 T
DC
Mono
NK
other
other T
h
scVAEIT
teadog-full
teadog-paired+full
teadog-paired-abc
teadog-paired-ab
teadog-paired-ac
teadog-paired-bc
teadog-diagonal+full
teadog-diagonal
Batch
LLL_Ctrl
LLL_Stim
DIG_Ctrl
DIG_Stim
Cell type
B
CD4 T
CD8 T
DC
Mono
NK
other
other T
i
StabMap
teadog−full
teadog−paired+full
teadog−paired−abc
teadog−paired−ab
teadog−paired−ac
teadog−paired−bc
teadog−diagonal+full
teadog−diagonal
Batch
W1
W6
LLL_Ctrl
DIG_Stim
Cell type
B
CD4 T
CD8 T
DC
Mono
NK
other
other T
j
Multigrate
teadog-full
teadog-paired+full
teadog-paired-abc
teadog-paired-ab
teadog-paired-ac
teadog-paired-bc
teadog-diagonal+full
teadog-diagonal
Batch
LLL_Ctrl
LLL_Stim
DIG_Ctrl
DIG_Stim
Cell type
B
CD4 T
CD8 T
DC
Mono
NK
other
other T

Supplementary Figure 9. UMAP comparison of embeddings on dogma (a–e) and teadog (f–j) mosaic integration tasks. In each task, cells in the top row are colored by batch, and cells in the bottom row are colored by cell type.

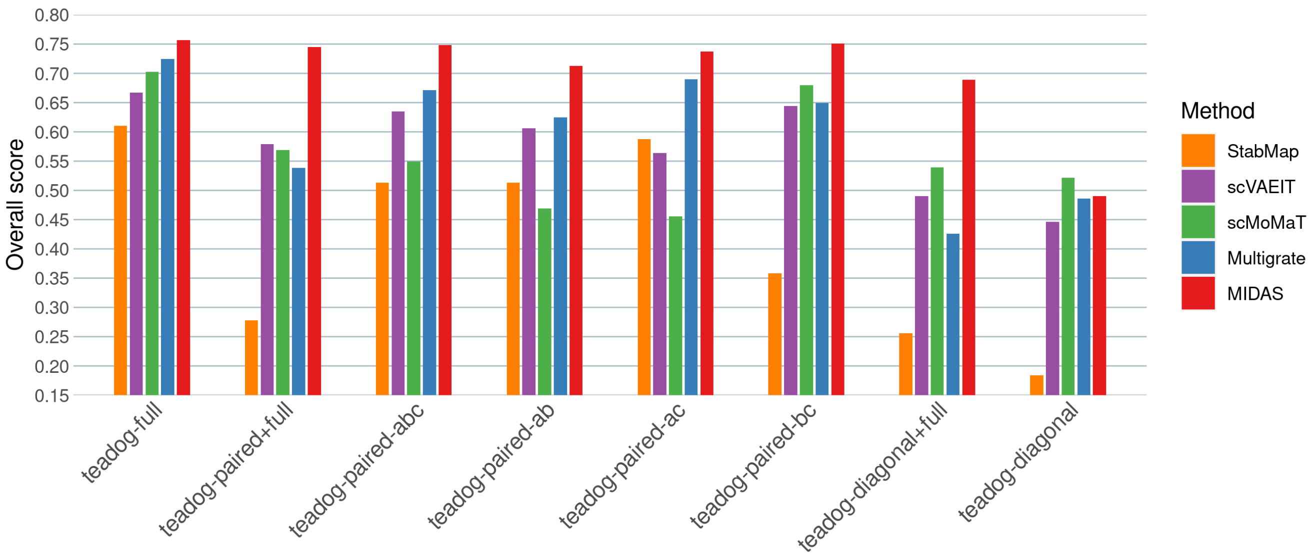

Supplementary Figure 10. Comparison of scIB overall scores on teadog mosaic integration tasks.

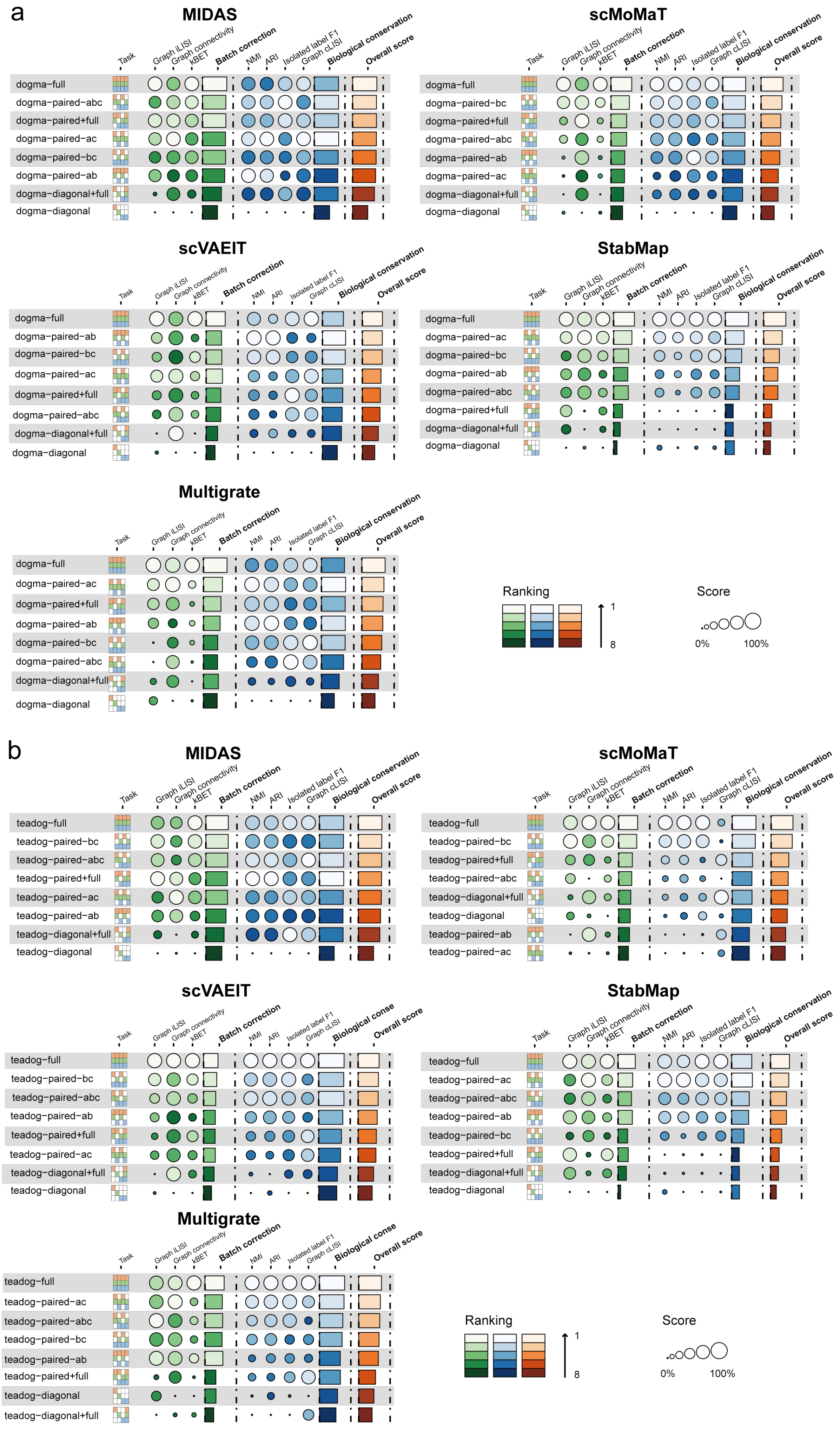

Supplementary Figure 11. Detailed comparison of scIB scores on dogma (a) and teadog (b) mosaic integration tasks.

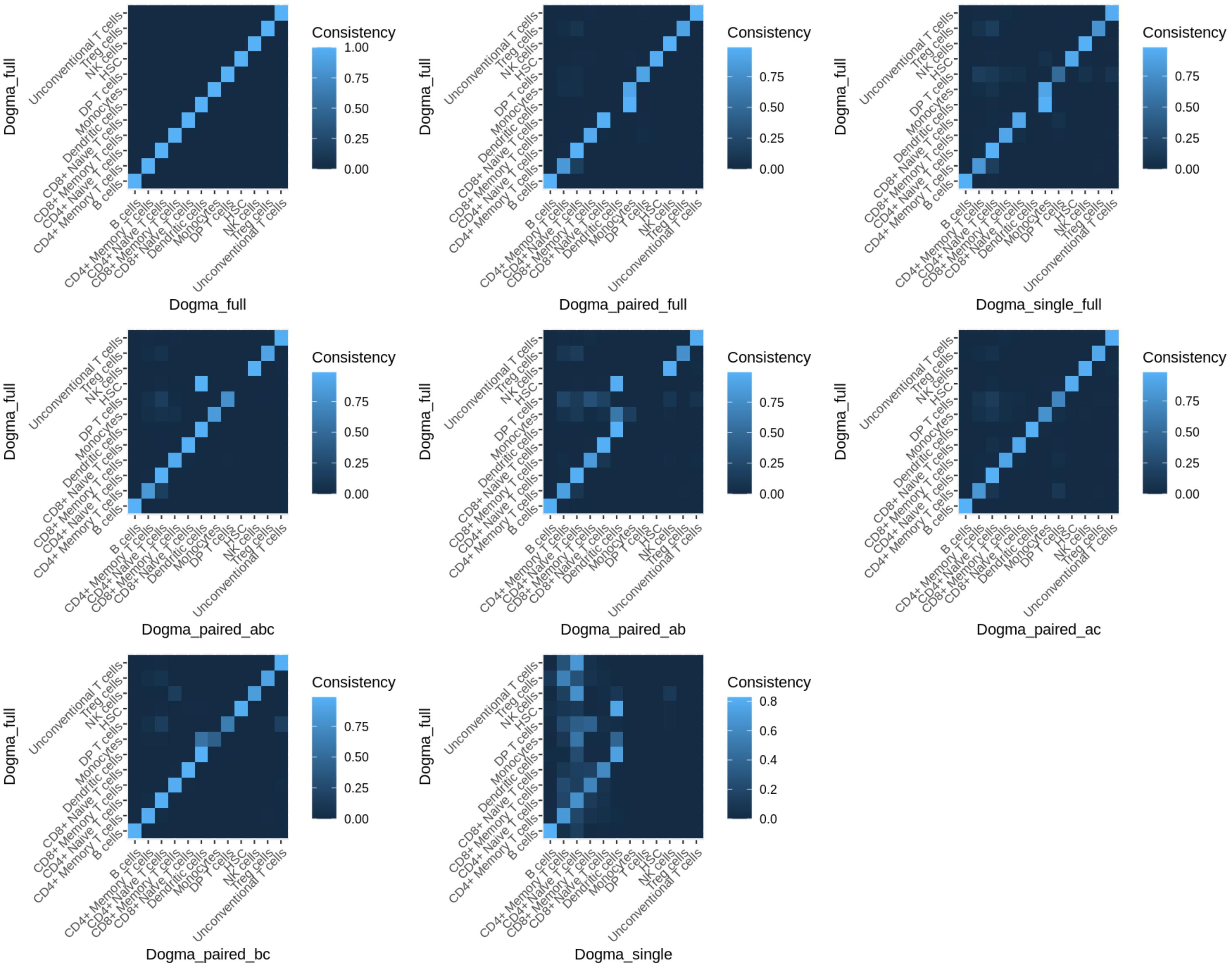

Supplementary Figure 12. Consistency of downstream labelling results got from different mosaic integration tasks. Each task result is compared to the dogma-full task result.

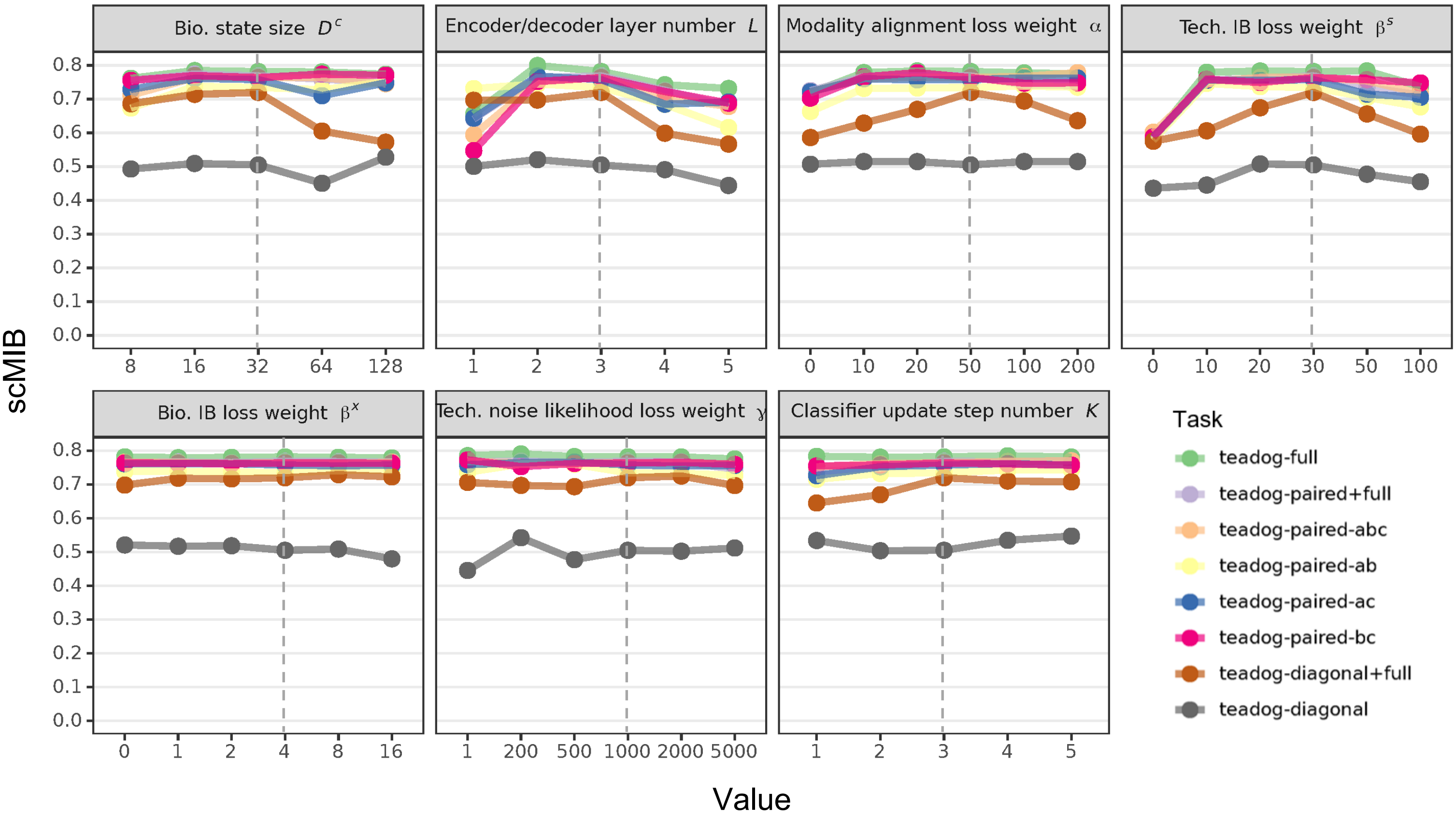

Supplementary Figure 13. Integration performance of MIDAS under different settings of hyperparameters. In each plot, the dashed line corresponds to the default value. To control computational costs, we only change one hyperparameter at a time while setting the others to their default values. The value "0" on the x-axis corresponds to the disability of the modality alignment module, technical information bottleneck module, and biological information bottleneck module, respectively. In the "Technical noise likelihood coefficient γ" panel, the value "1" on the x-axis represents the disability of the technical noise module. The teadog mosaic datasets were chosen as evaluation datasets, which are more challenging and compelling than the dogma mosaic datasets for highlighting the impact of changes in hyperparameters.

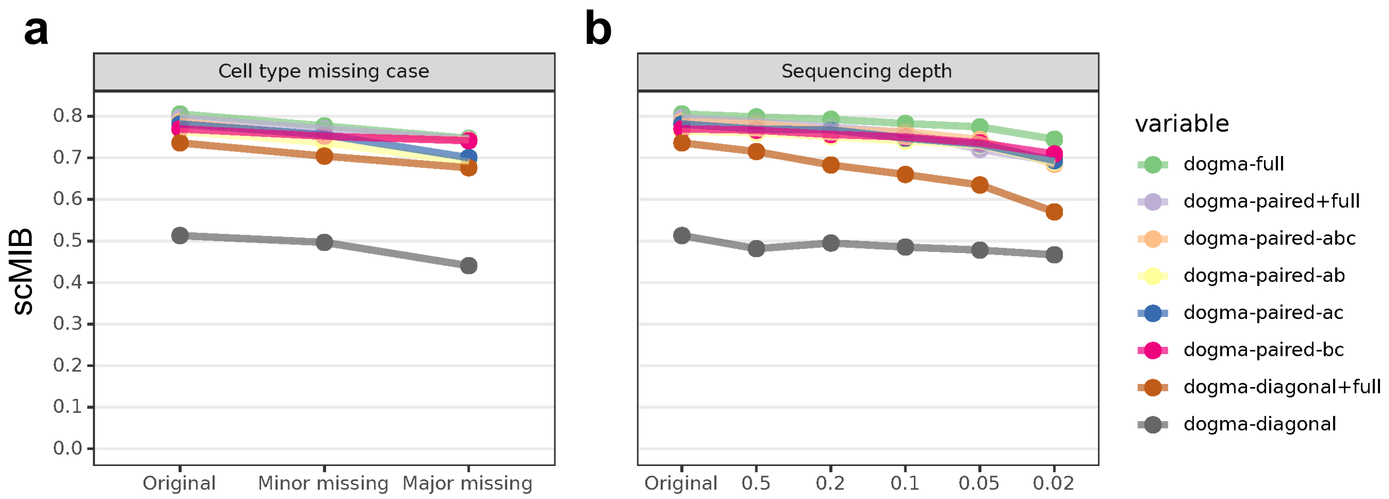

Supplementary Figure 14. Integration performance of MIDAS under different dataset variations including cell type missing (a) and sequencing depth changing (b). The dogma mosaic datasets were chosen as evaluation datasets, which exhibit fewer biological and technical variations across batches compared to the teadog mosaic datasets. This selection will be helpful in controlling the variation factors in the dataset, allowing for a clearer understanding of the impact of these changes.

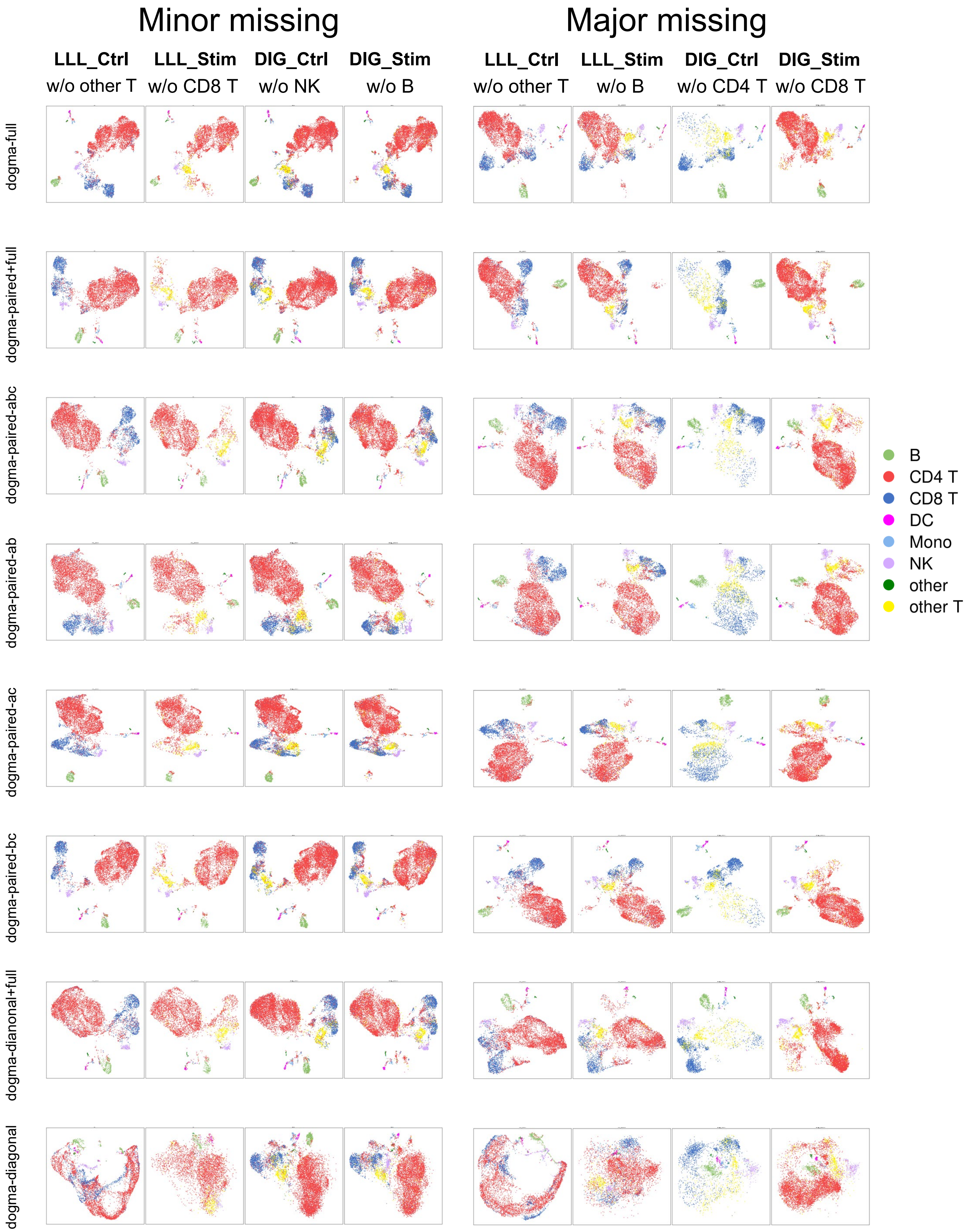

Supplementary Figure 15. UMAP comparison of MIDAS embeddings on dogma mosaic integration tasks with minor (left) and major (right) cell type missing.

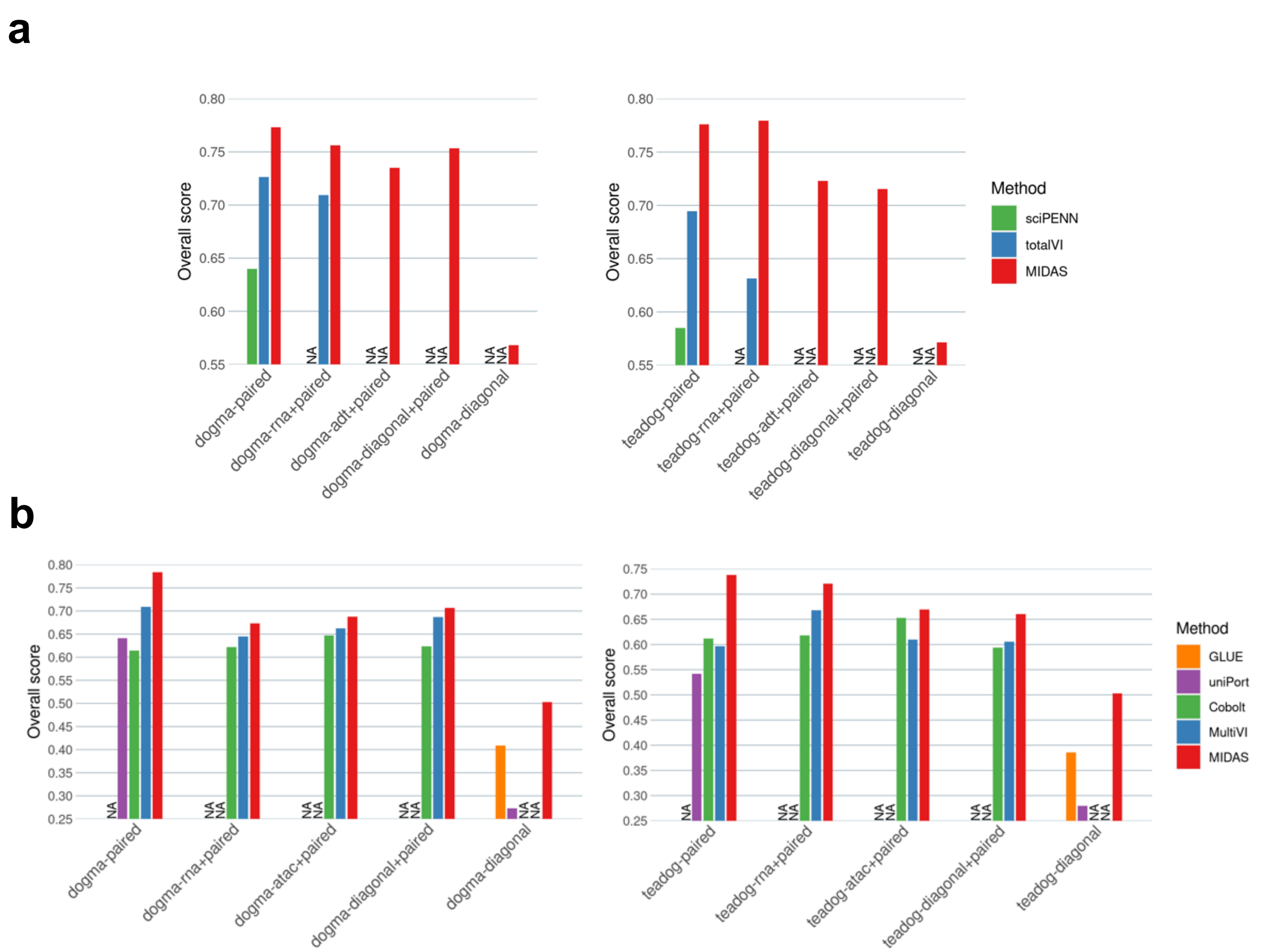

Supplementary Figure 16. Comparison of scIB overall scores on bimodal mosaic integration tasks. a, scIB overall scores of MIDAS, sciPENN and totalVI on RNA+ADT integration tasks based on dogma (left panel) and teadog (right panel) datasets. **b,** scIB overall scores of MIDAS, Cobolt, GLUE, MultiVI and uniPort on ATAC+RNA integration tasks based on dogma (left panel) and teadog (right panel) datasets.

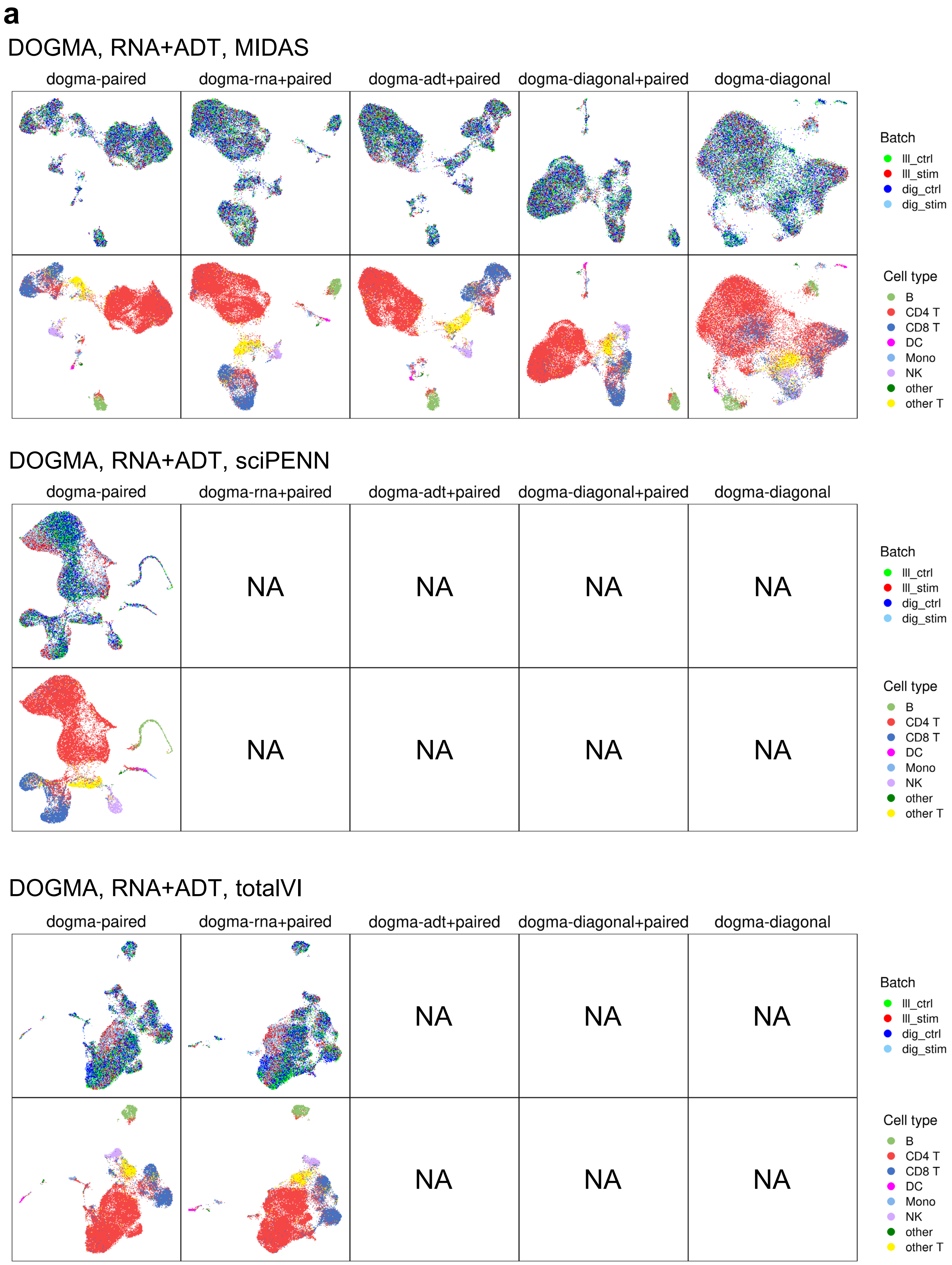
a
DOGMA, RNA+ADT, MIDAS
dogma-paired
dogma-rna+paired
dogma-adt+paired
dogma-diagonal+paired
dogma-diagonal
Batch
lll_ctrl
lll_stim
dig_ctrl
dig_stim
Cell type
B
CD4 T
CD8 T
DC
Mono
NK
other
other T
DOGMA, RNA+ADT, sciPENN
dogma-paired
dogma-rna+paired
dogma-adt+paired
dogma-diagonal+paired
dogma-diagonal
NA
NA
NA
NA
NA
NA
NA
NA
Batch
lll_ctrl
lll_stim
dig_ctrl
dig_stim
Cell type
B
CD4 T
CD8 T
DC
Mono
NK
other
other T
DOGMA, RNA+ADT, totalVI
dogma-paired
dogma-rna+paired
dogma-adt+paired
dogma-diagonal+paired
dogma-diagonal
NA
NA
NA
NA
NA
NA
Batch
lll_ctrl
lll_stim
dig_ctrl
dig_stim
Cell type
B
CD4 T
CD8 T
DC
Mono
NK
other
other T

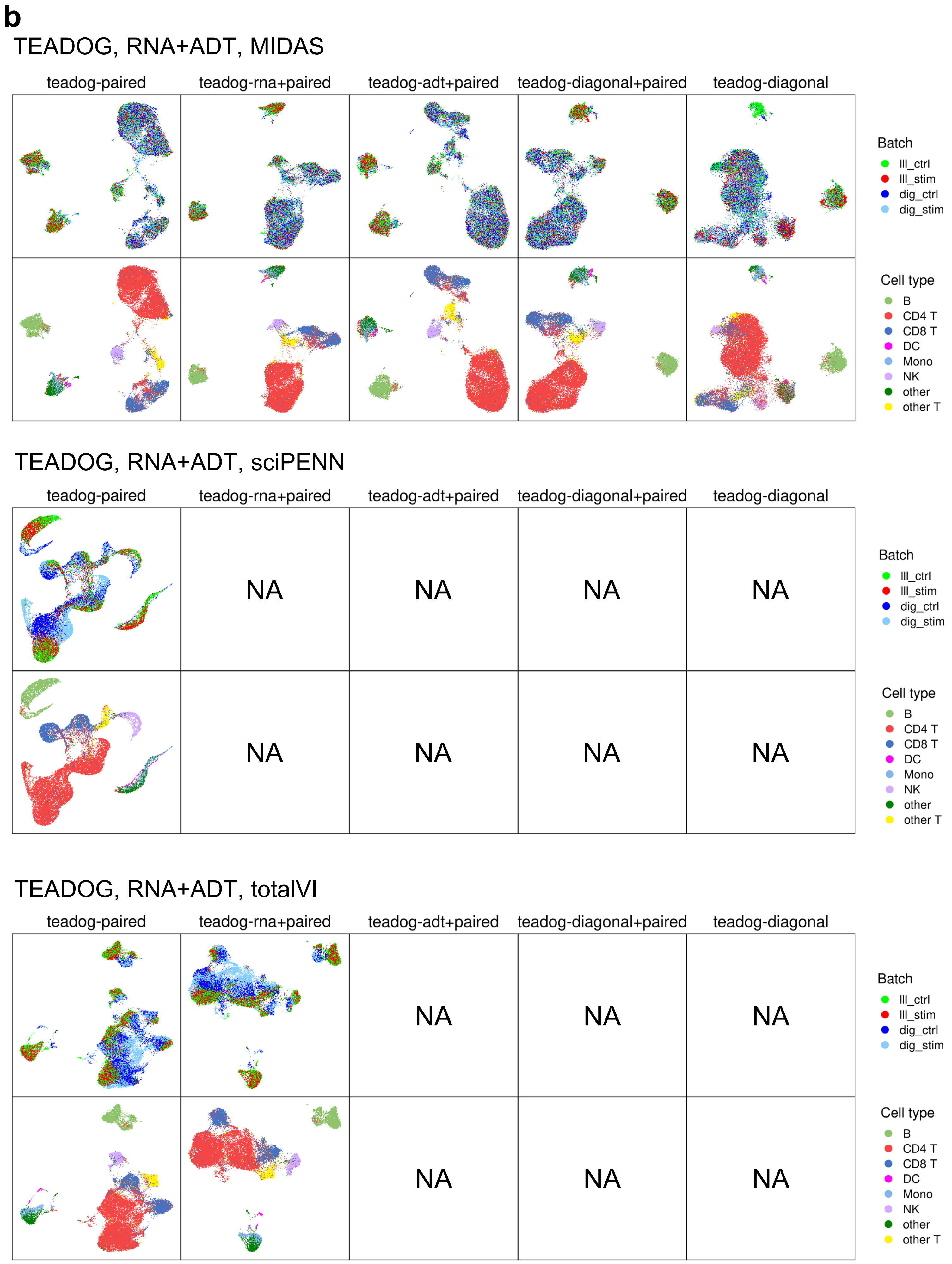
b
TEADOG, RNA+ADT, MIDAS
teadog-paired
teadog-rna+paired
teadog-adt+paired
teadog-diagonal+paired
teadog-diagonal
Batch
lll_ctrl
lll_stim
dig_ctrl
dig_stim
Cell type
B
CD4 T
CD8 T
DC
Mono
NK
other
other T
TEADOG, RNA+ADT, sciPENN
teadog-paired
teadog-rna+paired
teadog-adt+paired
teadog-diagonal+paired
teadog-diagonal
NA
NA
NA
NA
NA
NA
NA
NA
Batch
lll_ctrl
lll_stim
dig_ctrl
dig_stim
Cell type
B
CD4 T
CD8 T
DC
Mono
NK
other
other T
TEADOG, RNA+ADT, totalVI
teadog-paired
teadog-rna+paired
teadog-adt+paired
teadog-diagonal+paired
teadog-diagonal
NA
NA
NA
NA
NA
NA
Batch
lll_ctrl
lll_stim
dig_ctrl
dig_stim
Cell type
B
CD4 T
CD8 T
DC
Mono
NK
other
other T

c

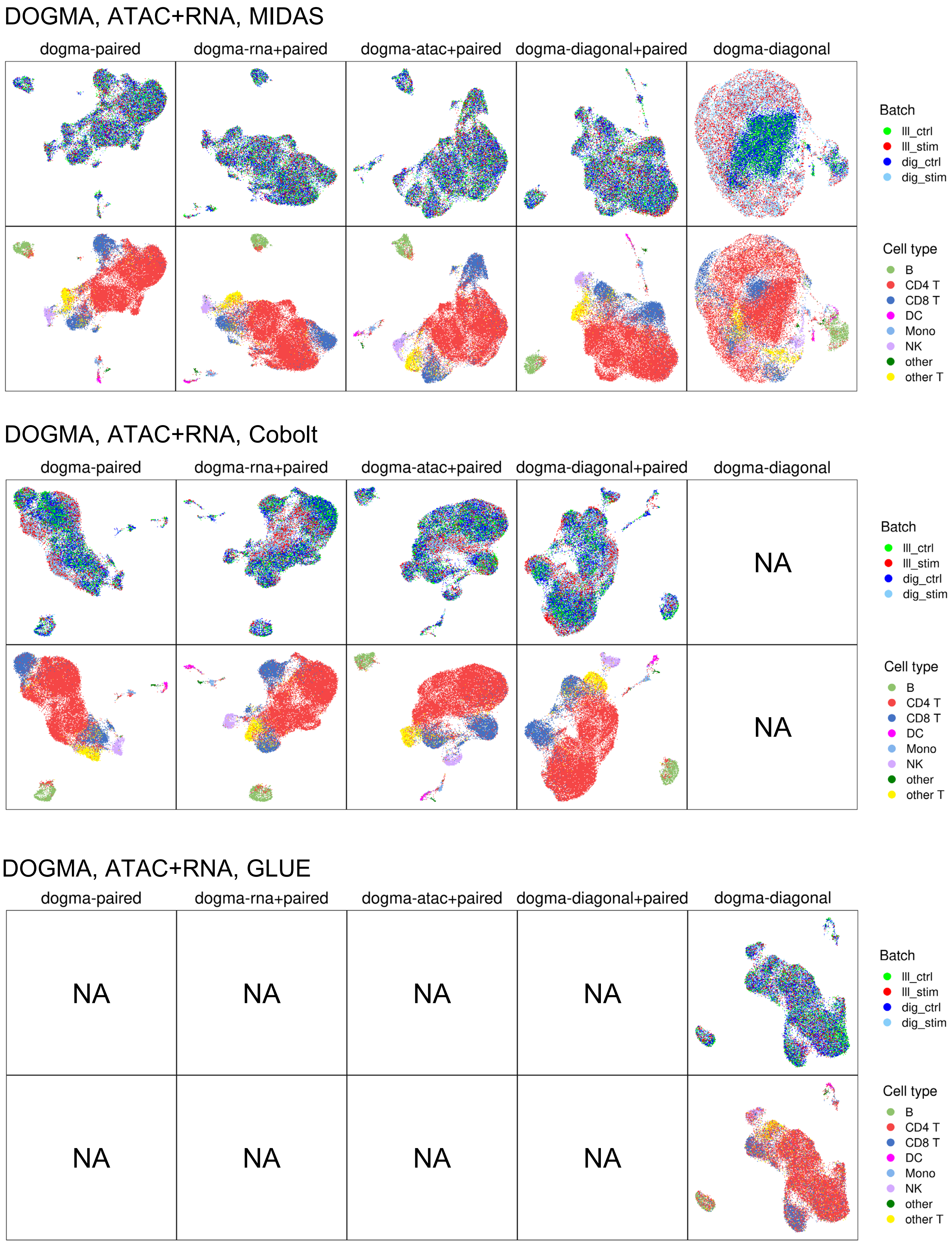
DOGMA, ATAC+RNA, MIDAS
dogma-paired
dogma-rna+paired
dogma-atac+paired
dogma-diagonal+paired
dogma-diagonal
Batch
lll_ctrl
lll_stim
dig_ctrl
dig_stim
Cell type
B
CD4 T
CD8 T
DC
Mono
NK
other
other T
DOGMA, ATAC+RNA, Cobolt
NA
DOGMA, ATAC+RNA, GLUE
NA

DOGMA, ATAC+RNA, MultiVI

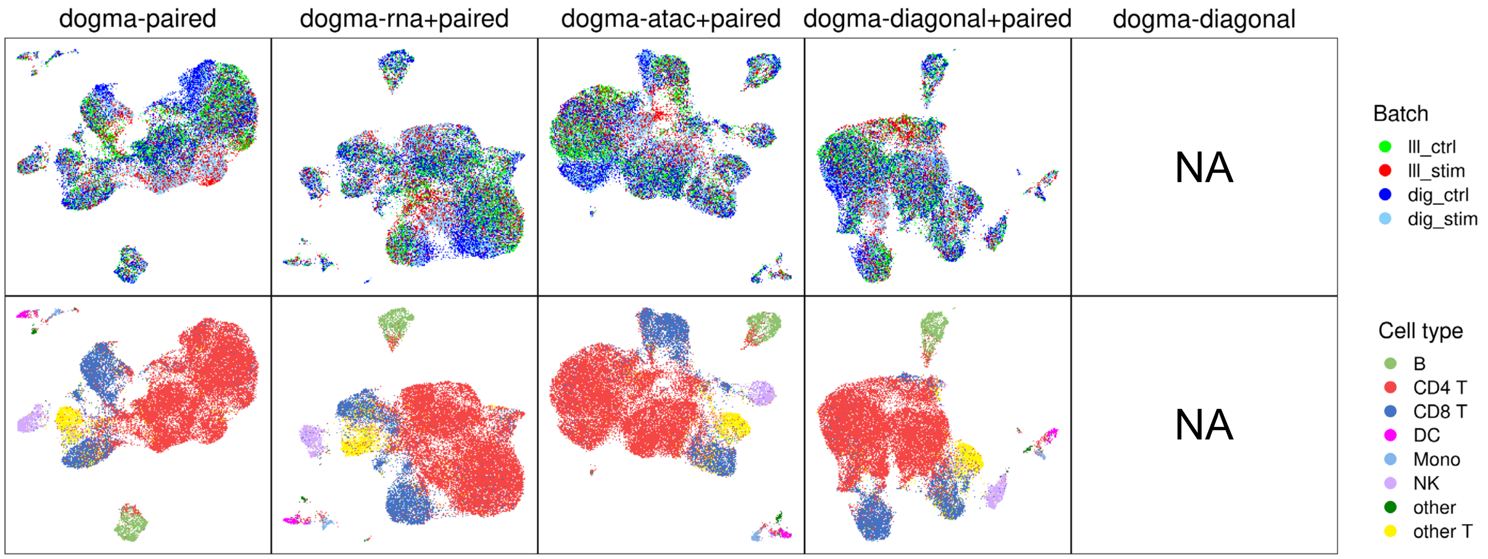

DOGMA, ATAC+RNA, uniPort

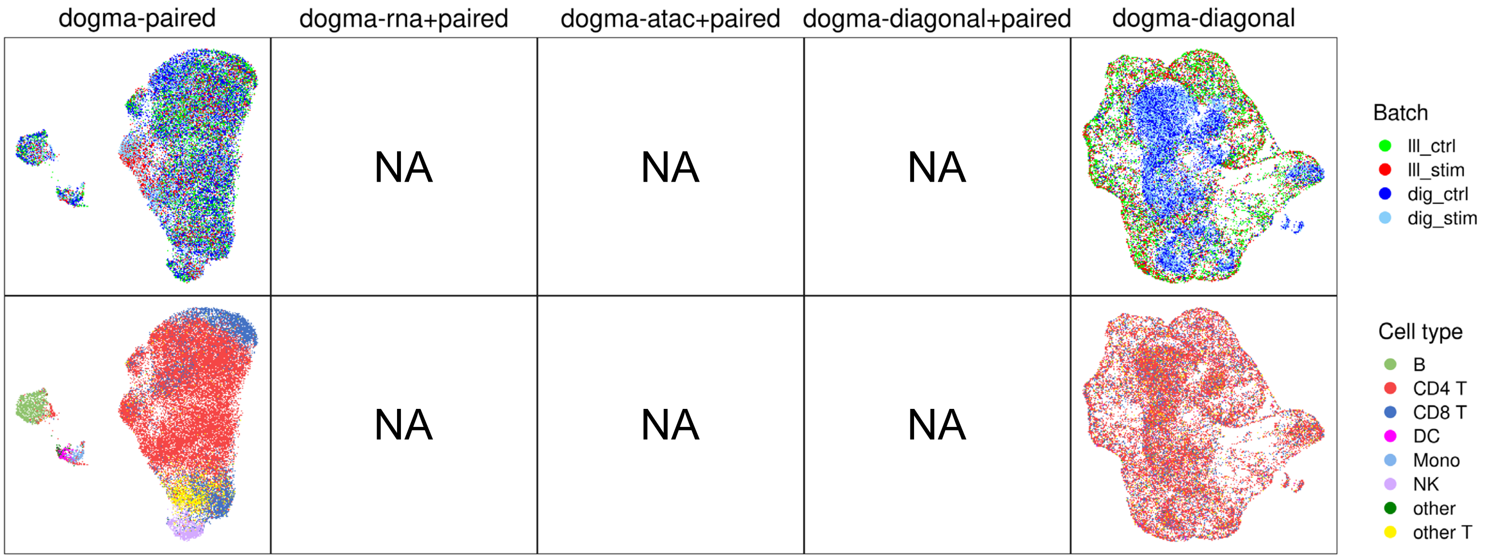

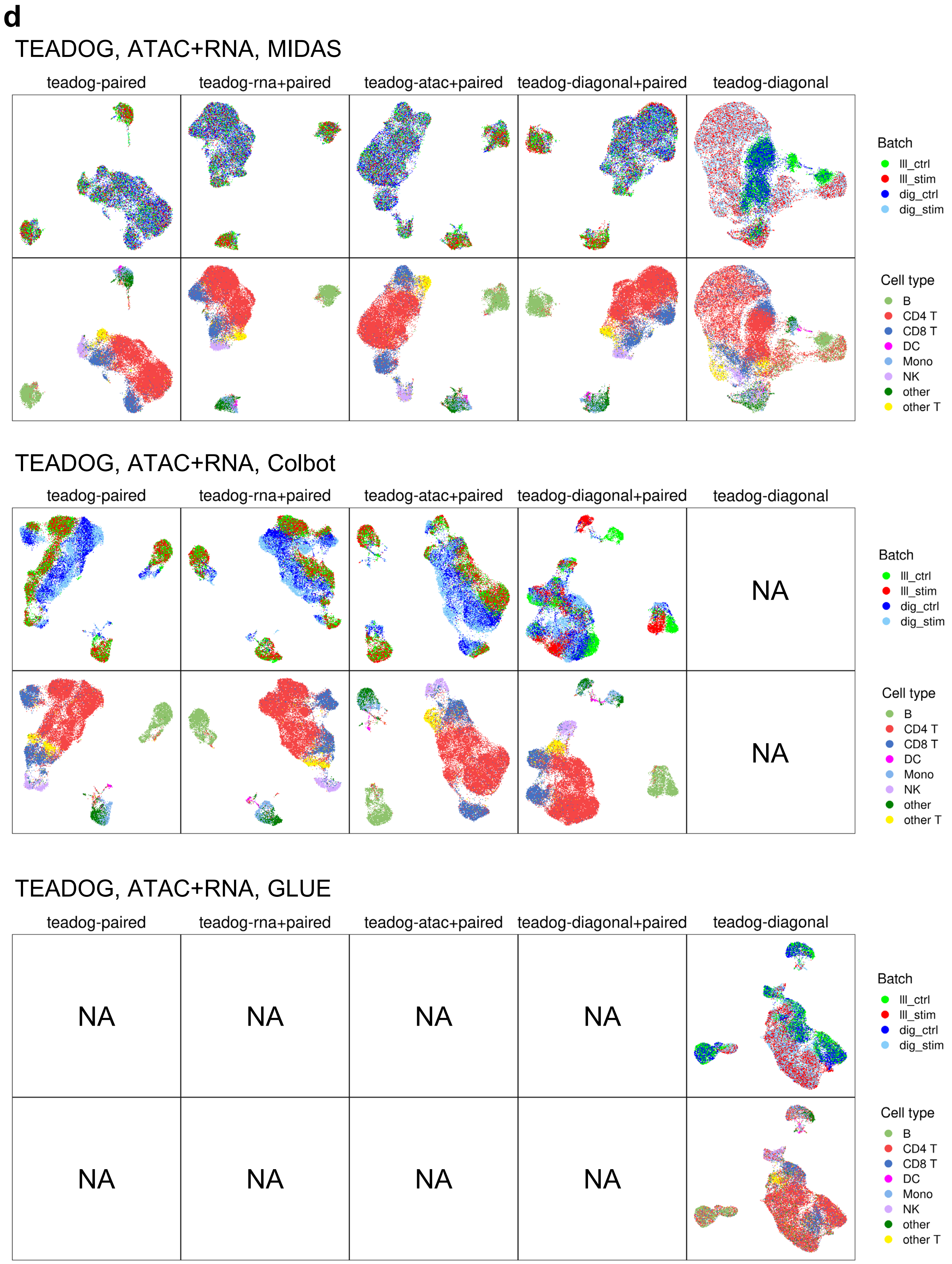
d
TEADOG, ATAC+RNA, MIDAS
teadog-paired
teadog-rna+paired
teadog-atac+paired
teadog-diagonal+paired
teadog-diagonal
Batch
lll_ctrl
lll_stim
dig_ctrl
dig_stim
Cell type
B
CD4 T
CD8 T
DC
Mono
NK
other
other T
TEADOG, ATAC+RNA, Colbot
teadog-paired
teadog-rna+paired
teadog-atac+paired
teadog-diagonal+paired
teadog-diagonal
NA
NA
Batch
lll_ctrl
lll_stim
dig_ctrl
dig_stim
Cell type
B
CD4 T
CD8 T
DC
Mono
NK
other
other T
TEADOG, ATAC+RNA, GLUE
teadog-paired
teadog-rna+paired
teadog-atac+paired
teadog-diagonal+paired
teadog-diagonal
NA
NA
NA
NA
NA
NA
NA
NA
Batch
lll_ctrl
lll_stim
dig_ctrl
dig_stim
Cell type
B
CD4 T
CD8 T
DC
Mono
NK
other
other T

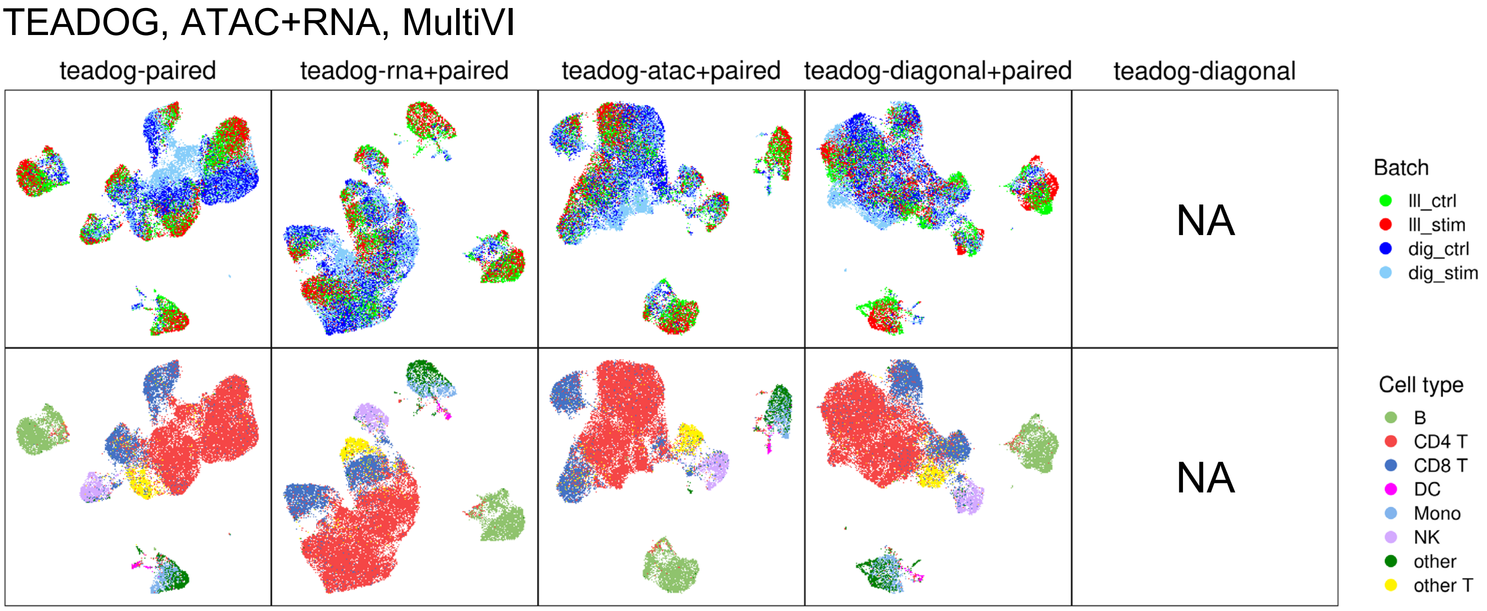

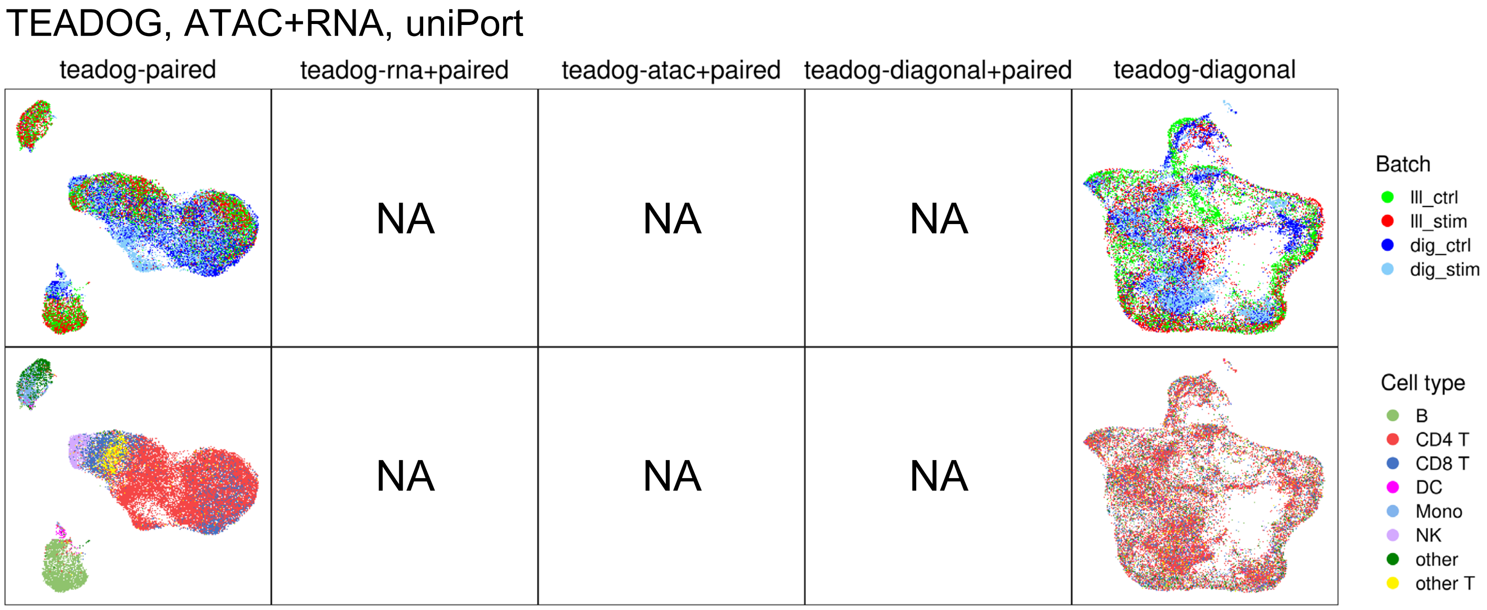

Supplementary Figure 17. UMAP comparison of performance of MIDAS and other methods on bimodal integration tasks. a, **b**, UMAP comparison of MIDAS, scPENN and totalVI on RNA+ADT integration tasks based on dogma (a) and teadog (b) datasets. **c**, **d**, UMAP comparison of MIDAS, Cobolt, GLUE, MultiVI and uniPort on ATAC+RNA integration tasks based on dogma (c) and teadog (d) datasets. NA, not available.

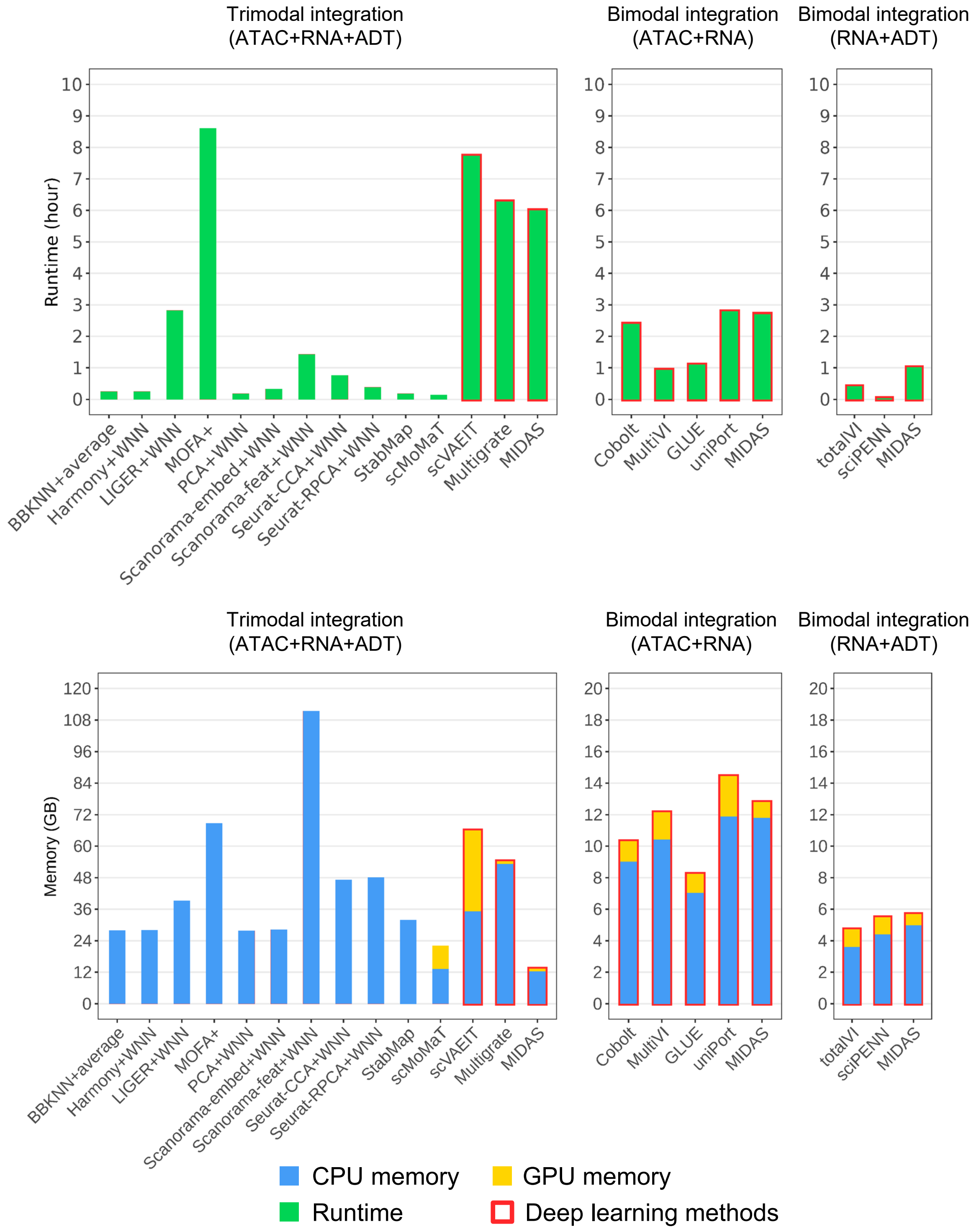

Supplementary Figure 18. Comparison of the runtime (top) and memory (bottom) costs of different methods. All methods are evaluated on a Linux server with 36 CPU cores (two Intel Xeon Gold 6240 chips), 512 GB of RAM, and one NVIDIA Tesla V100S GPU.

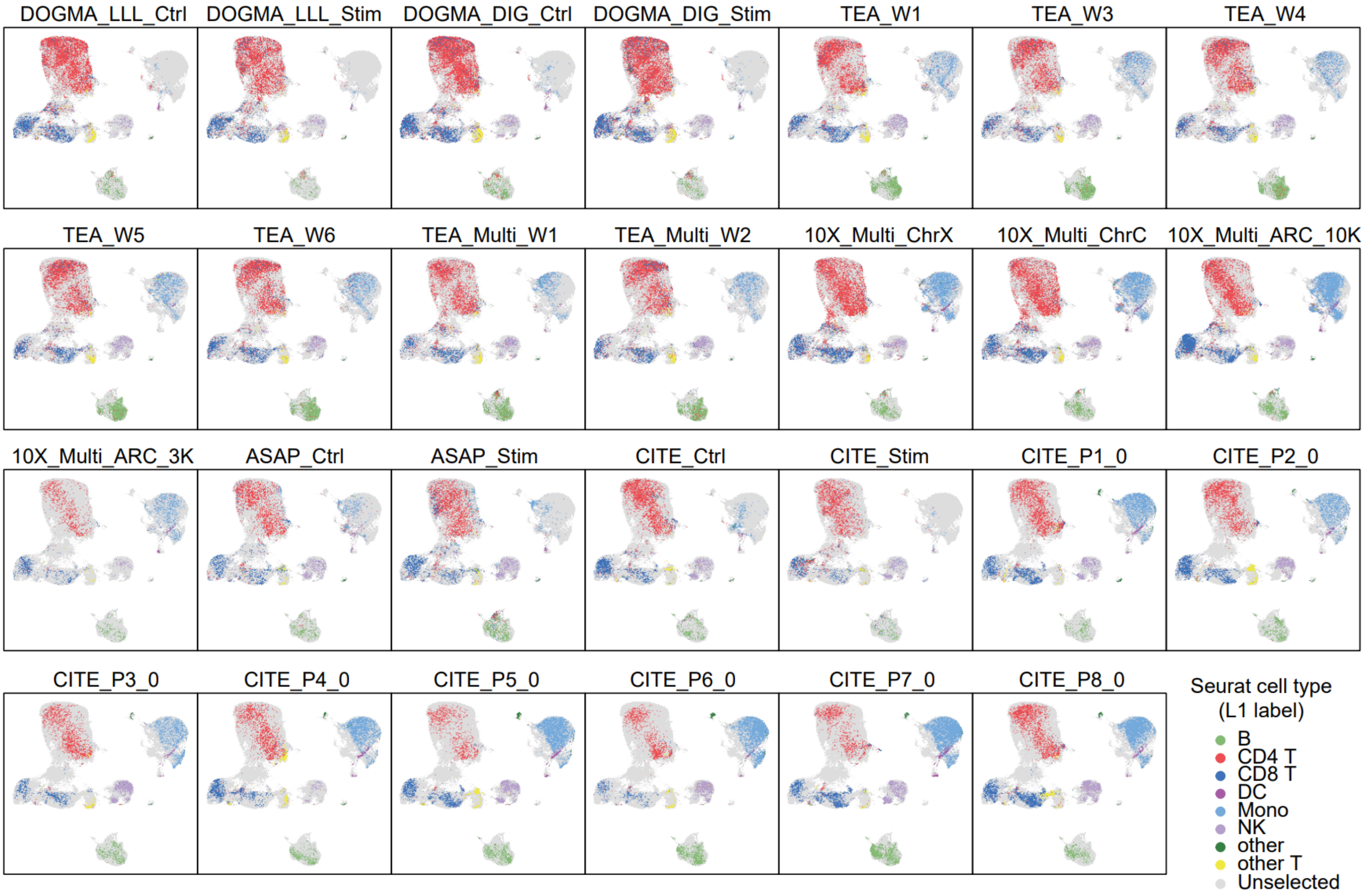

Supplementary Figure 19. UMAP visualization of the biological states inferred by MIDAS with the PBMC mosaic dataset containing 27 batches. Cell type labels are derived from Seurat labeling.

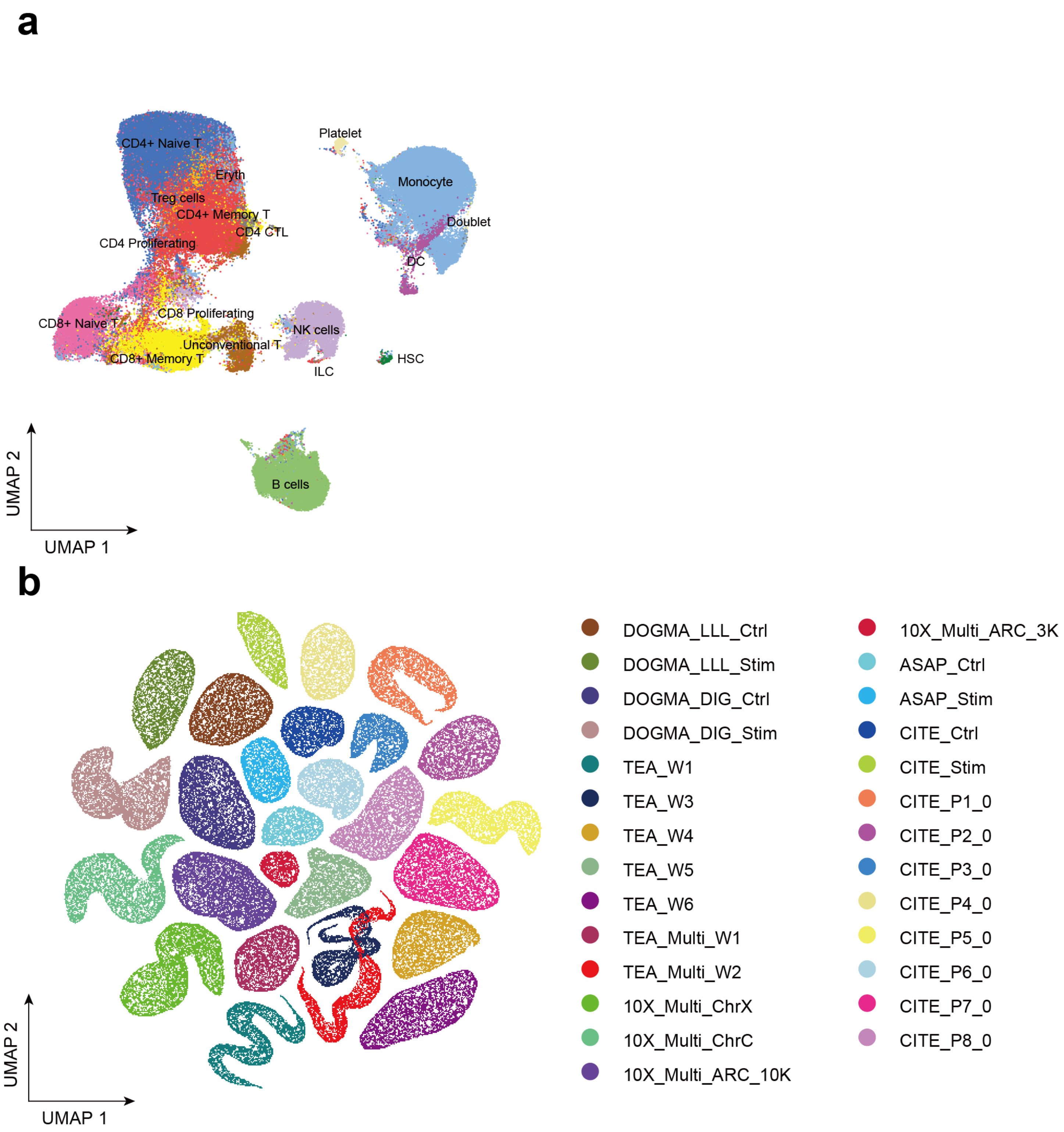

Supplementary Figure 20. UMAP visualization (merged view) of the biological states (a) and technical noises (b) inferred by MIDAS with the PBMC mosaic dataset containing 27 batches. Cell type colors are derived from Seurat labeling.

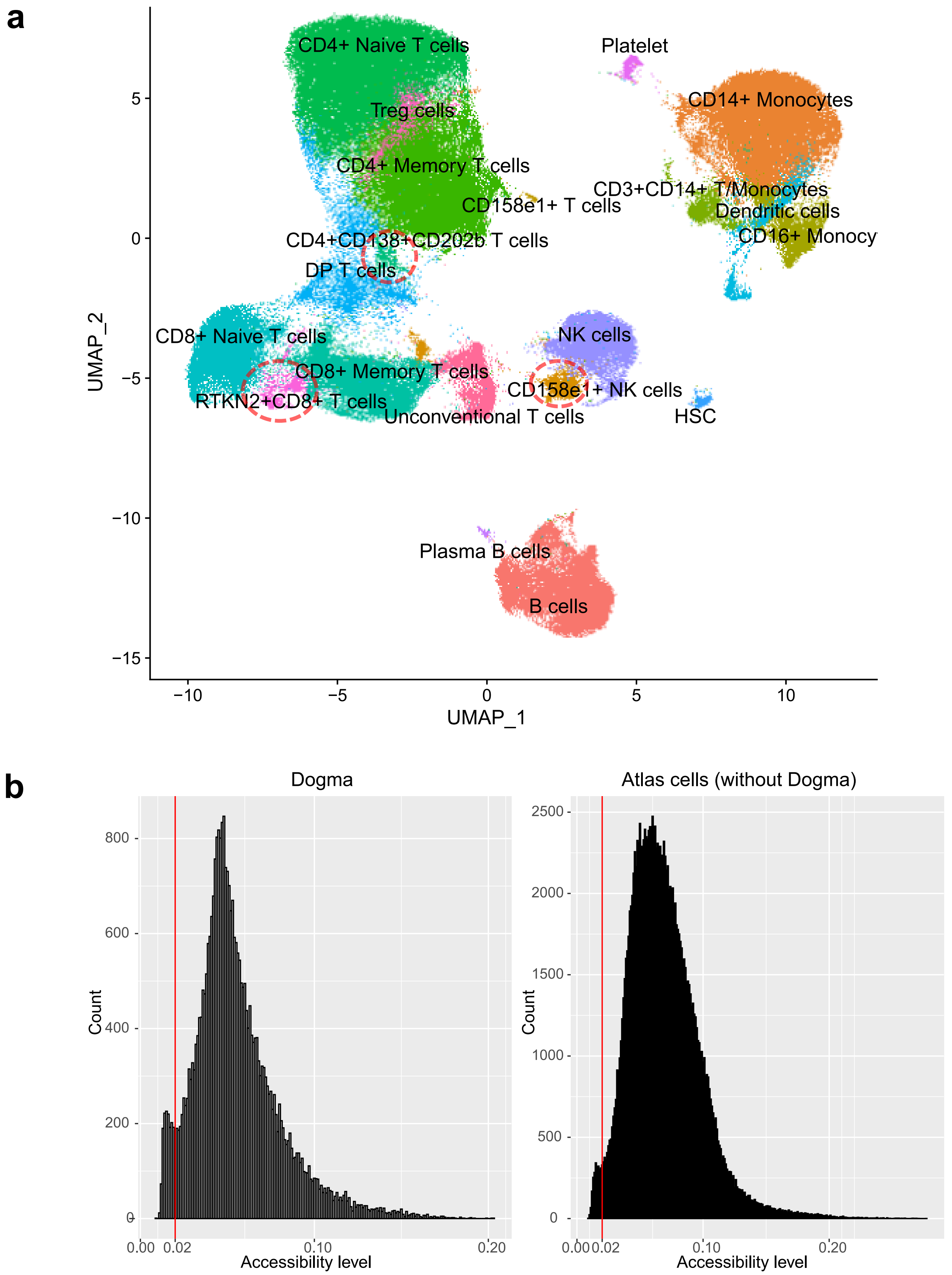

Supplementary Figure 21. The Atlas leads to a high-resolution cell typing and LCA cells validation. a, A more refined cell typing result of MIDAS on the atlas. **b**, The bimodal distribution of ATAC peak ration in the dogma (left) cells and atlas (right) cells without dogma. Peak ratio of a cell is defined as the ratio of peak numbers of the cell to that of all the cells

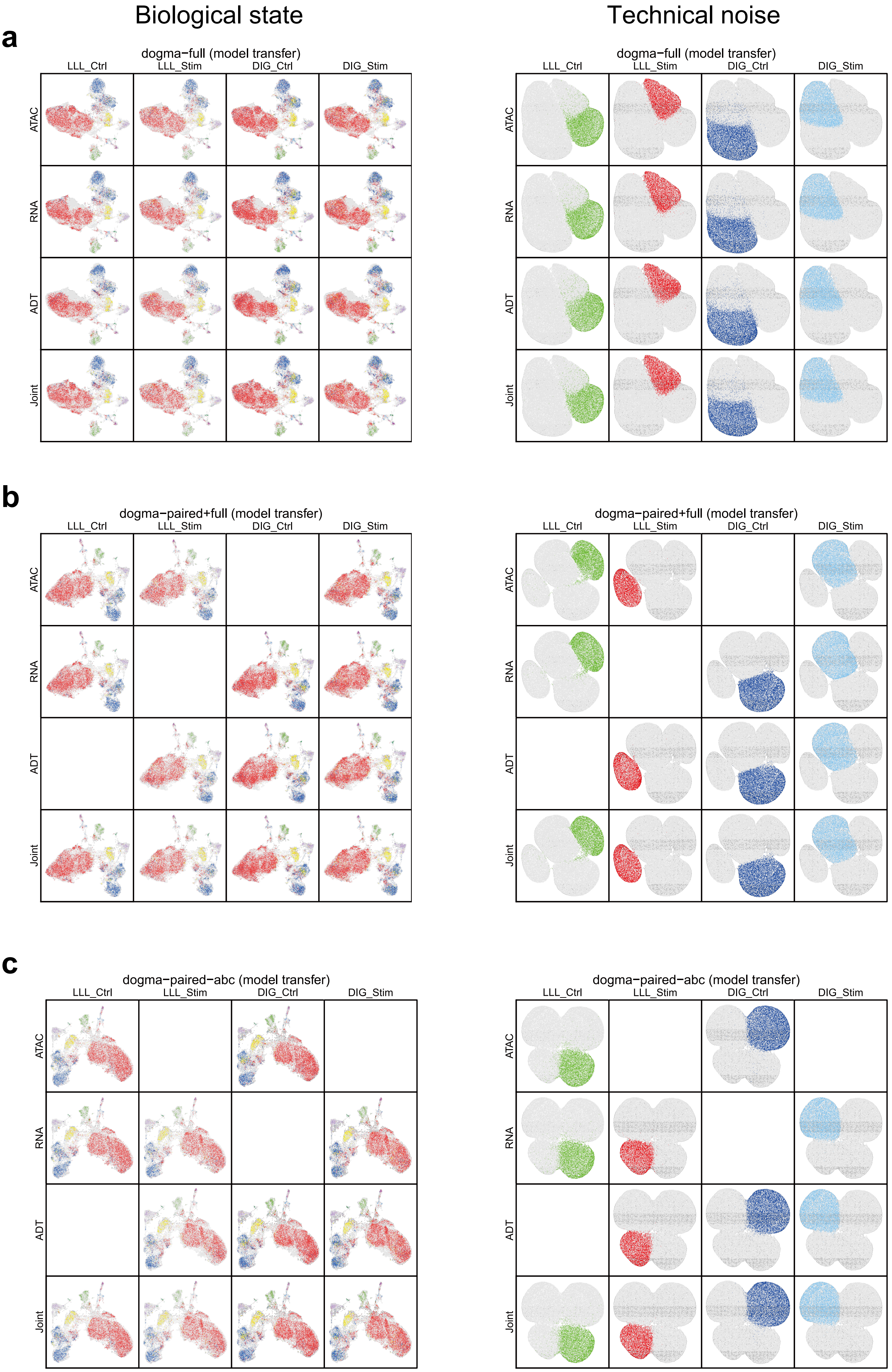
Biological state
Technical noise
a
dogma−full (model transfer)
LLL_Ctrl
LLL_Stim
DIG_Ctrl
DIG_Stim
ATAC
RNA
ADT
Joint
b
dogma−paired+full (model transfer)
c
dogma−paired−abc (model transfer)

d

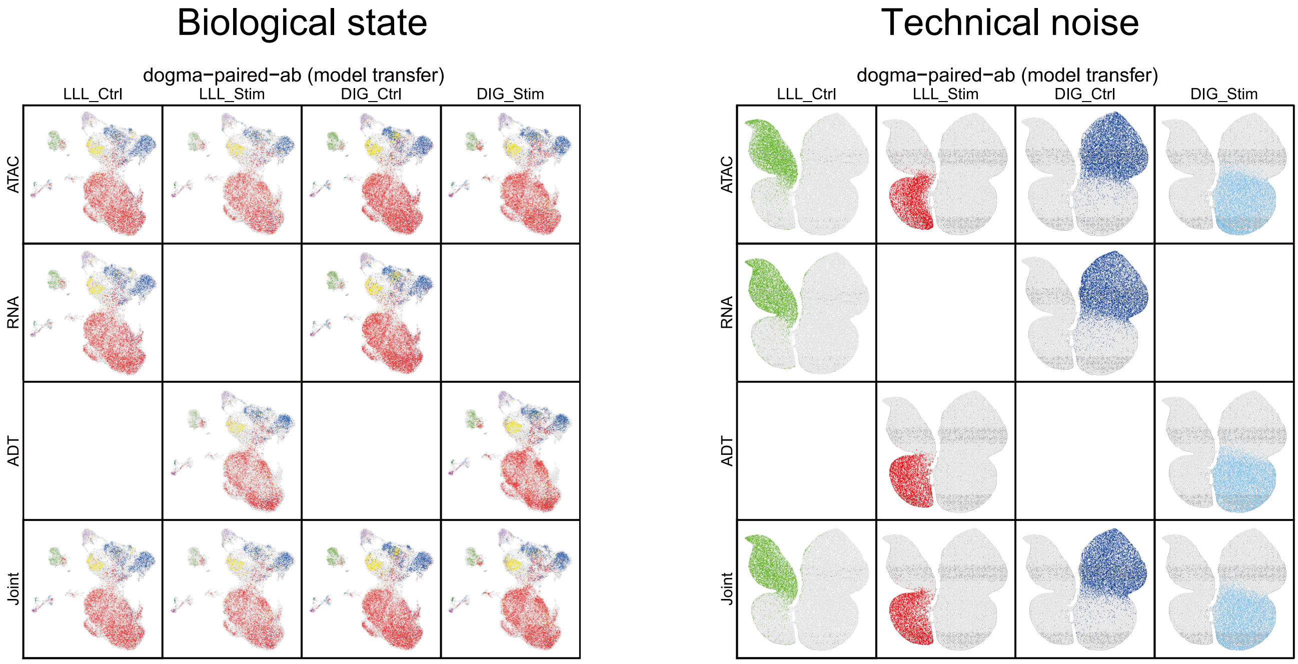
Biological state
Technical noise
dogma−paired−ab (model transfer)
LLL_Ctrl
LLL_Stim
DIG_Ctrl
DIG_Stim
ATAC
RNA
ADT
Joint
dogma−paired−ab (model transfer)
LLL_Ctrl
LLL_Stim
DIG_Ctrl
DIG_Stim
ATAC
RNA
ADT
Joint

e

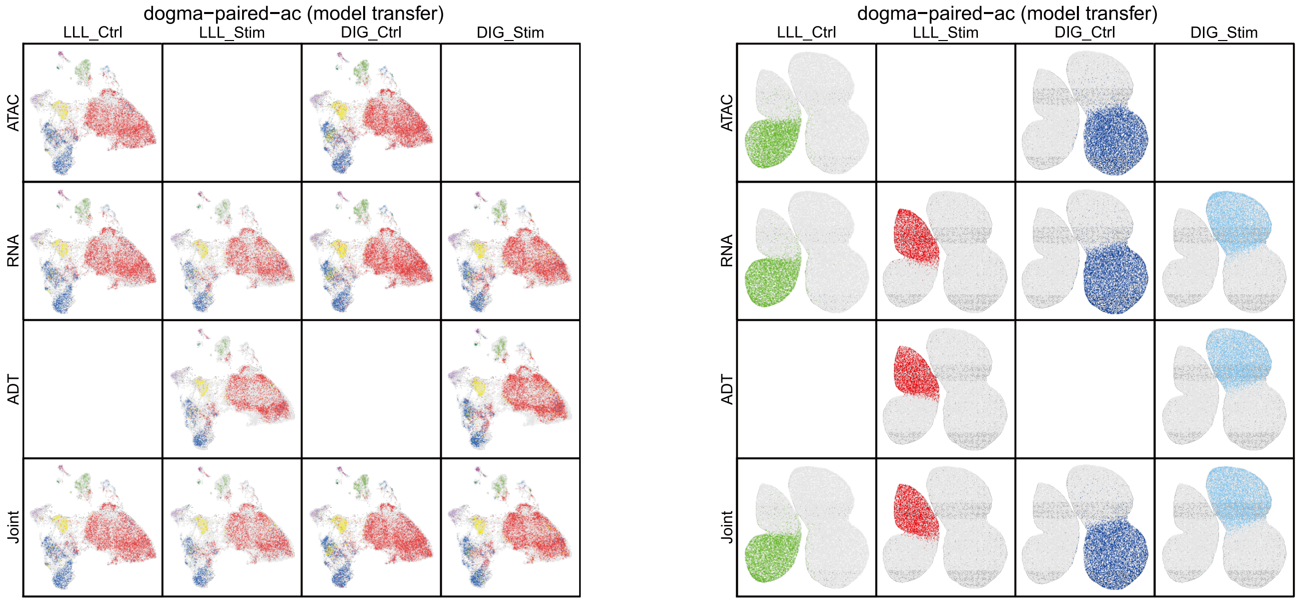
dogma−paired−ac (model transfer)
LLL_Ctrl
LLL_Stim
DIG_Ctrl
DIG_Stim
ATAC
RNA
ADT
Joint
dogma−paired−ac (model transfer)
LLL_Ctrl
LLL_Stim
DIG_Ctrl
DIG_Stim
ATAC
RNA
ADT
Joint

f

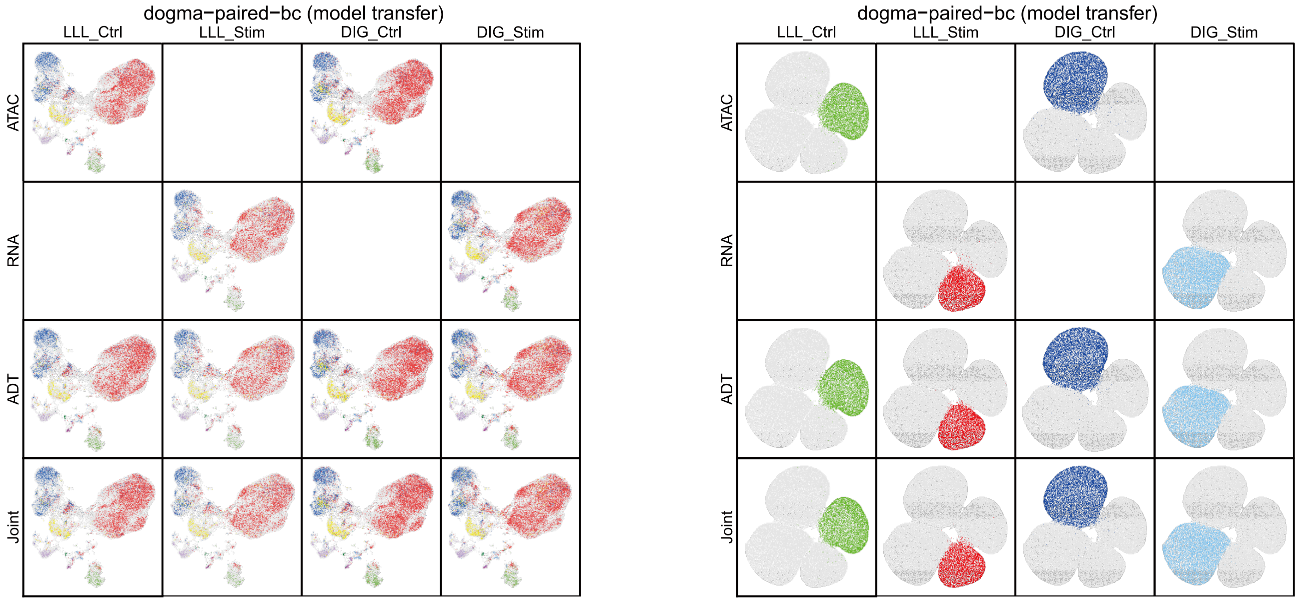
dogma−paired−bc (model transfer)
LLL_Ctrl
LLL_Stim
DIG_Ctrl
DIG_Stim
ATAC
RNA
ADT
Joint
dogma−paired−bc (model transfer)
LLL_Ctrl
LLL_Stim
DIG_Ctrl
DIG_Stim
ATAC
RNA
ADT
Joint

Biological state

Technical noise

g

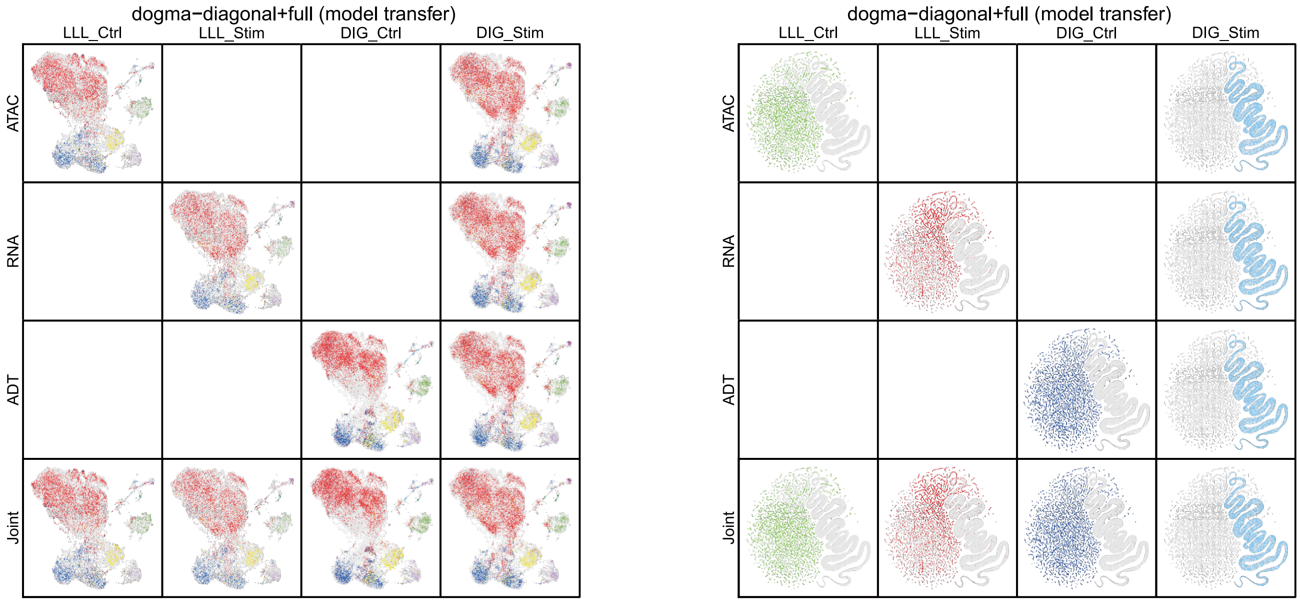

h

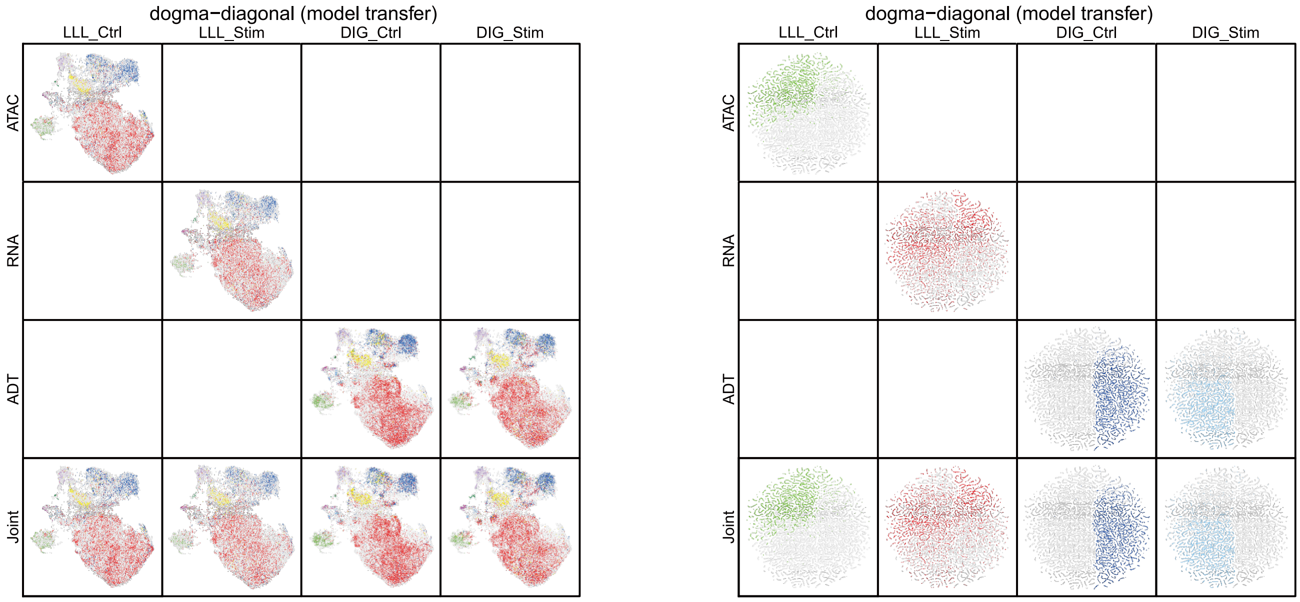

i

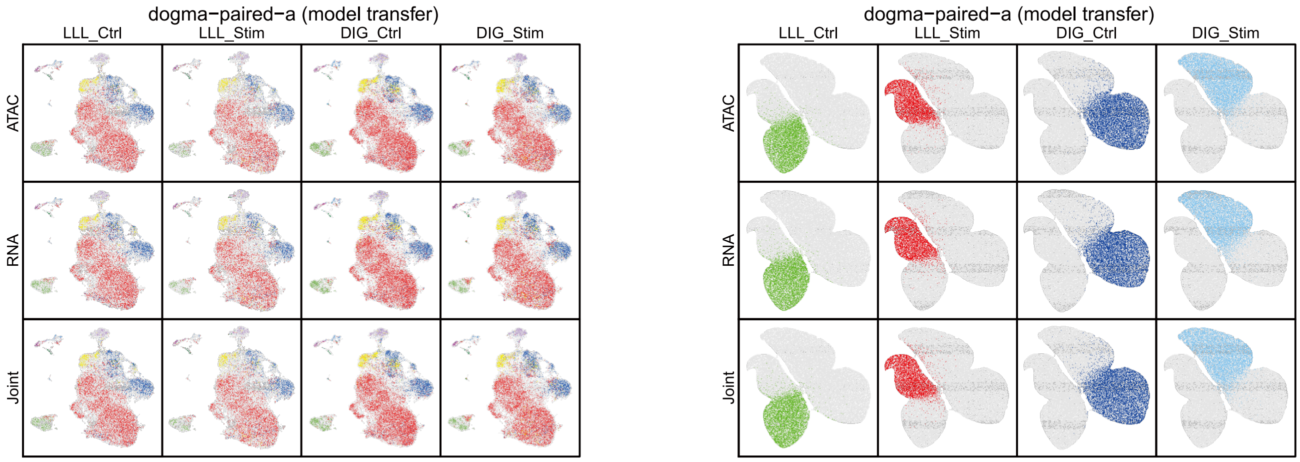

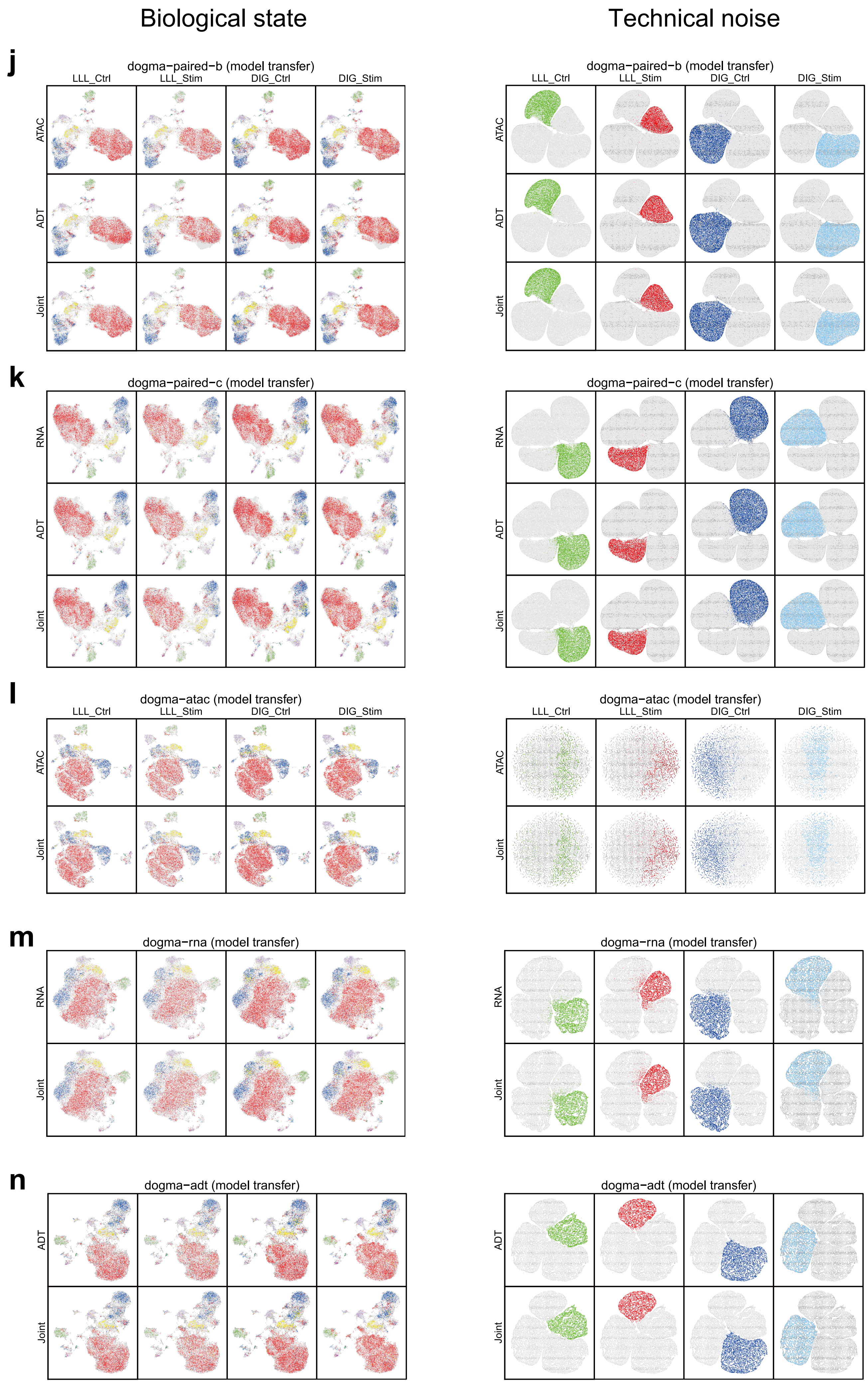

Biological state
Technical noise
j
dogma−paired−b (model transfer)
LLL_Ctrl
LLL_Stim
DIG_Ctrl
DIG_Stim
ATAC
ADT
Joint
dogma−paired−b (model transfer)
LLL_Ctrl
LLL_Stim
DIG_Ctrl
DIG_Stim
ATAC
ADT
Joint
k
dogma−paired−c (model transfer)
RNA
ADT
Joint
dogma−paired−c (model transfer)
RNA
ADT
Joint
l
dogma−atac (model transfer)
LLL_Ctrl
LLL_Stim
DIG_Ctrl
DIG_Stim
ATAC
Joint
dogma−atac (model transfer)
LLL_Ctrl
LLL_Stim
DIG_Ctrl
DIG_Stim
ATAC
Joint
m
dogma−rna (model transfer)
RNA
Joint
dogma−rna (model transfer)
RNA
Joint
n
dogma−adt (model transfer)
ADT
Joint
dogma−adt (model transfer)
ADT
Joint

Supplementary Figure 22. UMAP visualization of the biological states (left, colored by cell type) and technical noise (right, colored by batch) inferred by the transfer-learned MIDAS on 14 different dogma mosaic datasets (a–n).

a

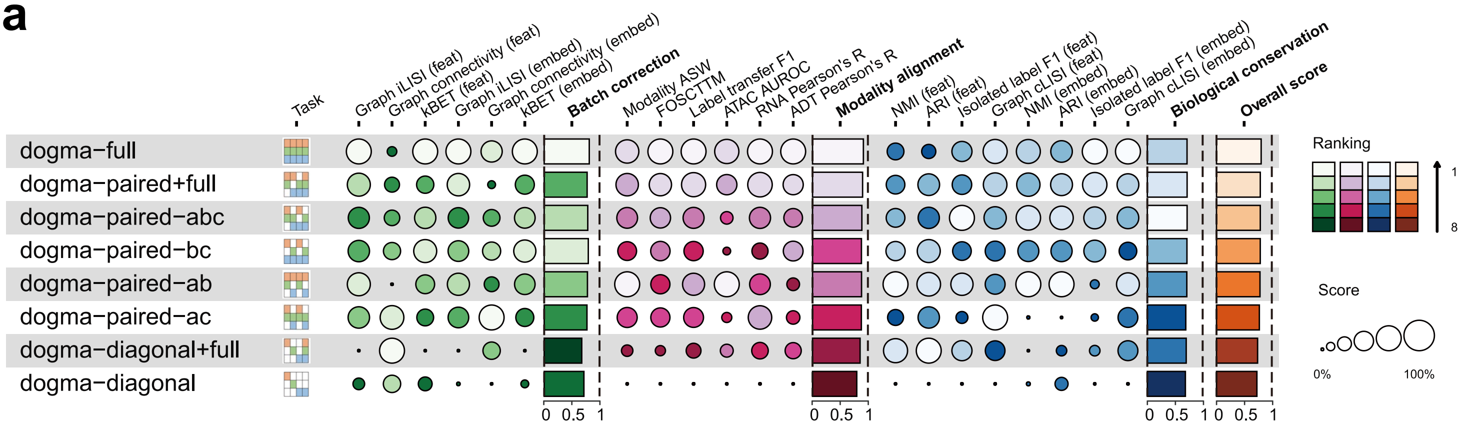

b

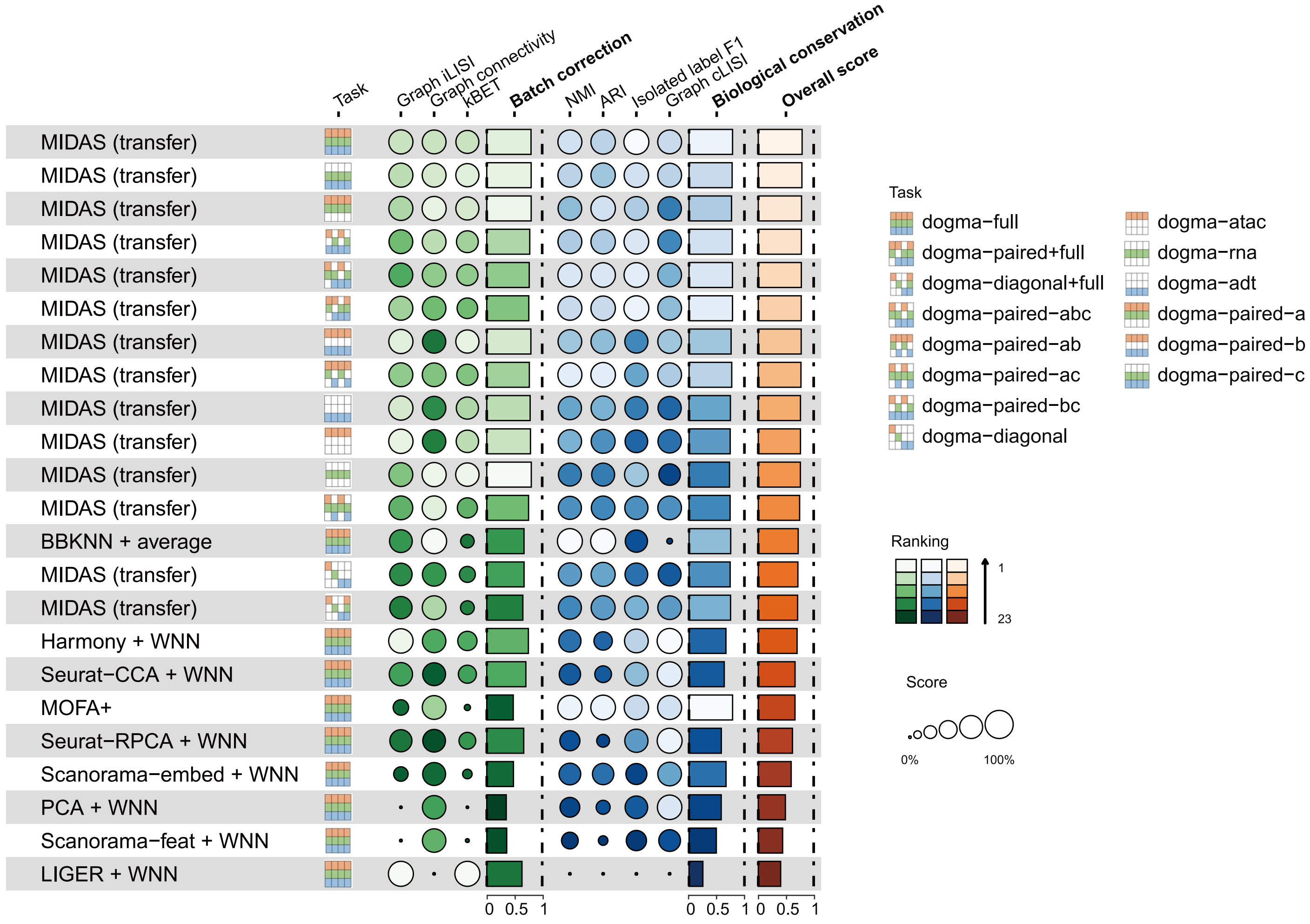

Supplementary Figure 23. scIB and scMIB benchmarking of the performance of the transfer-learned MIDAS and other strategies. a, Benchmarking the performance of transfer-learned MIDAS on dogma mosaic integration tasks using our proposed scMIB. **b**, scIB benchmarking the performance of transfer-learned MIDAS on dogma mosaic integration tasks against nine other strategies on the dogma-full rectangular integration task.

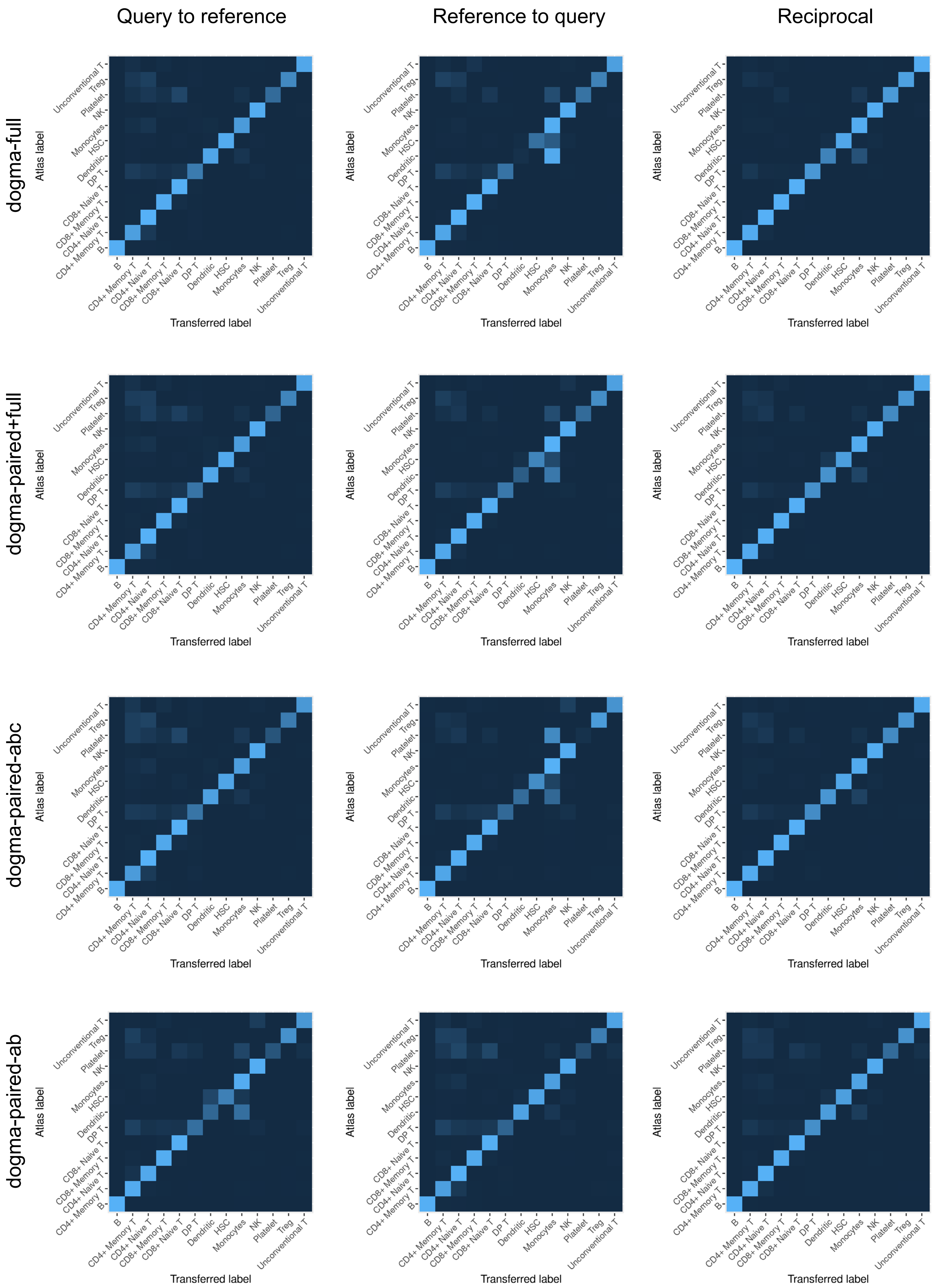

Query to reference
Reference to query
Reciprocal
dogma-full
dogma-paired+full
dogma-paired-abc
dogma-paired-ab
Atlas label
Transferred label
B
CD4+ Memory T
CD4+ Naive T
CD8+ Memory T
CD8+ Naive T
DP T
Dendritic
HSC
Monocytes
NK
Platelet
Treg
Unconventional T

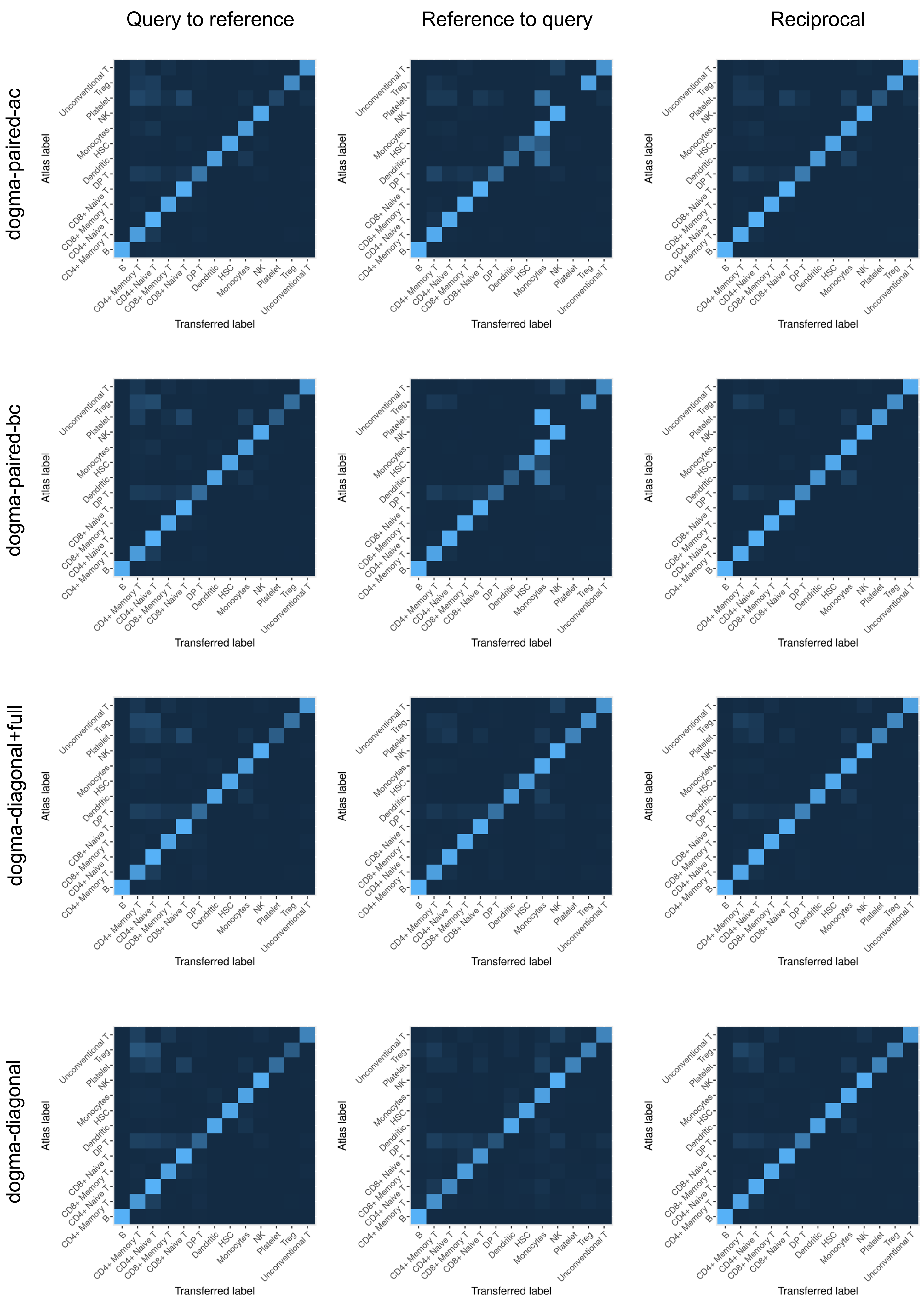

Query to reference
Reference to query
Reciprocal
dogma-paired-ac
dogma-paired-bc
dogma-diagonal+full
dogma-diagonal
Atlas label
Transferred label
B
CD4+ Memory T
CD4+ Naive T
CD8+ Memory T
CD8+ Naive T
DP T
Dendritic
HSC
Monocytes
NK
Platelet
Treg
Unconventional T

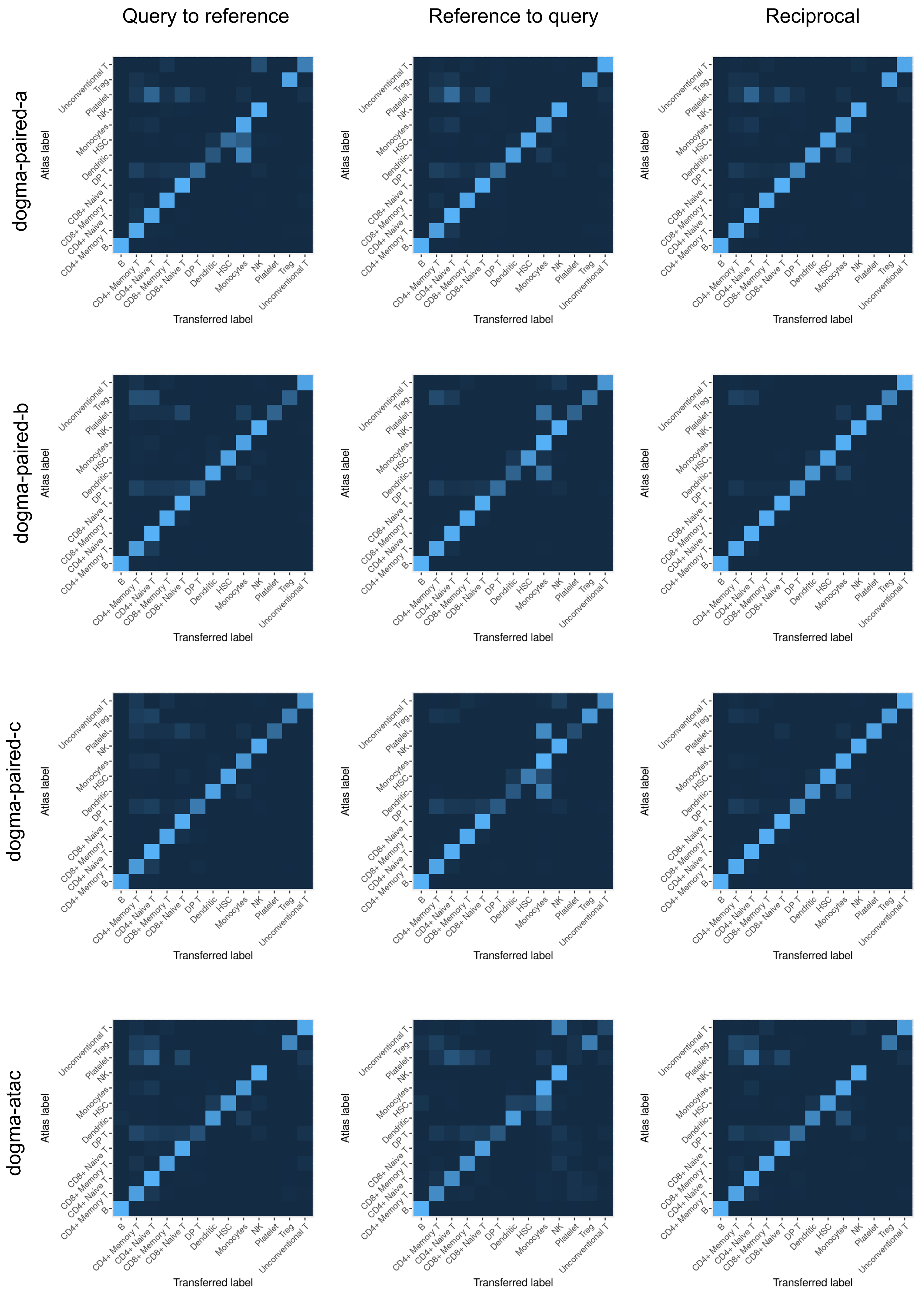

Query to reference
Reference to query
Reciprocal
dogma-paired-a
dogma-paired-b
dogma-paired-c
dogma-atac
Atlas label
Transferred label
B
CD4+ Memory T
CD4+ Naive T
CD8+ Memory T
CD8+ Naive T
DP T
Dendritic
HSC
Monocytes
NK
Platelet
Treg
Unconventional T

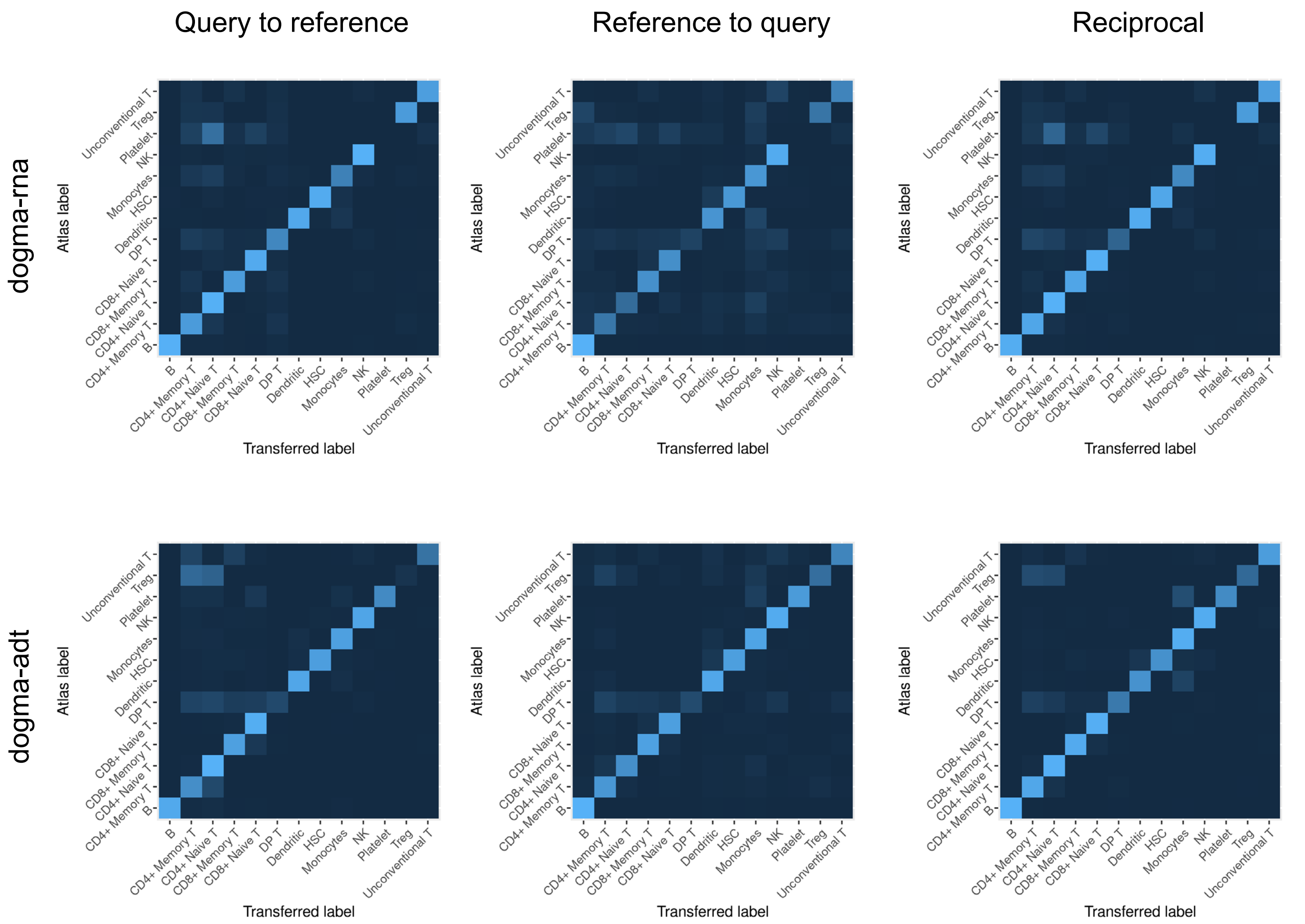

Supplementary Figure 24. Comparison of confusion plots corresponding to the label transfer micro F1-scores of different mapping strategies on different dogma query dataset. In each plot, rows correspond to PBMC atlas labels and columns correspond to transferred labels. The horizontal plots show 14 tasks while the vertical plots show query-to-reference mapping, reference-to-query mapping and reciprocal reference mapping methods.

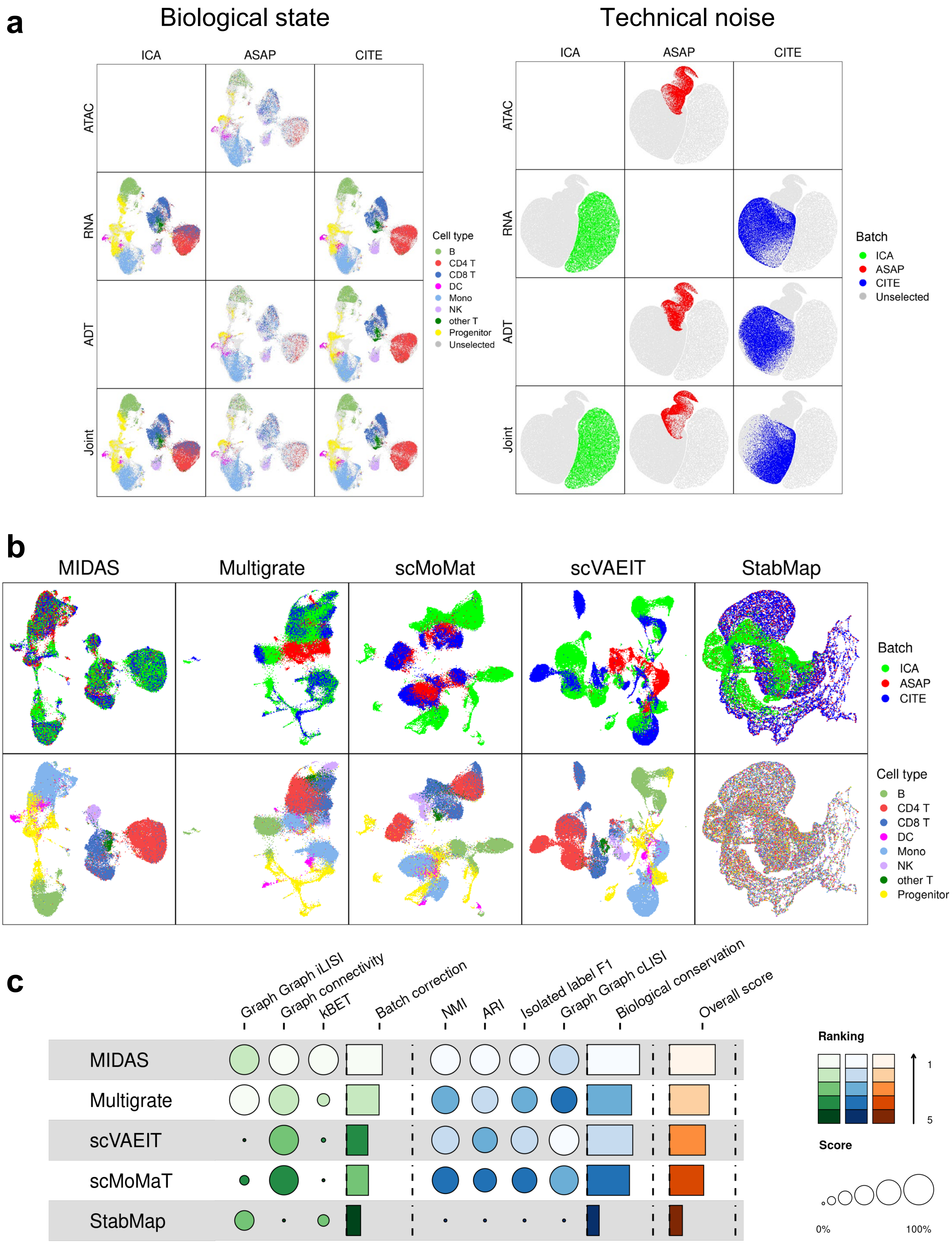

Supplementary Figure 25. Qualitative and quantitative evaluation of MIDAS performance on BMMC mosaic dataset. a, UMAP visualization of the biological states (left panel) and technical noises (right panel) inferred by MIDAS. **b,** UMAP comparison of embeddings on BMMC mosaic integration. Cells in the top row are colored by batches, and cells in the bottom row are colored by cell types. **c,** scIB benchmarking the performance of MIDAS against four methods.

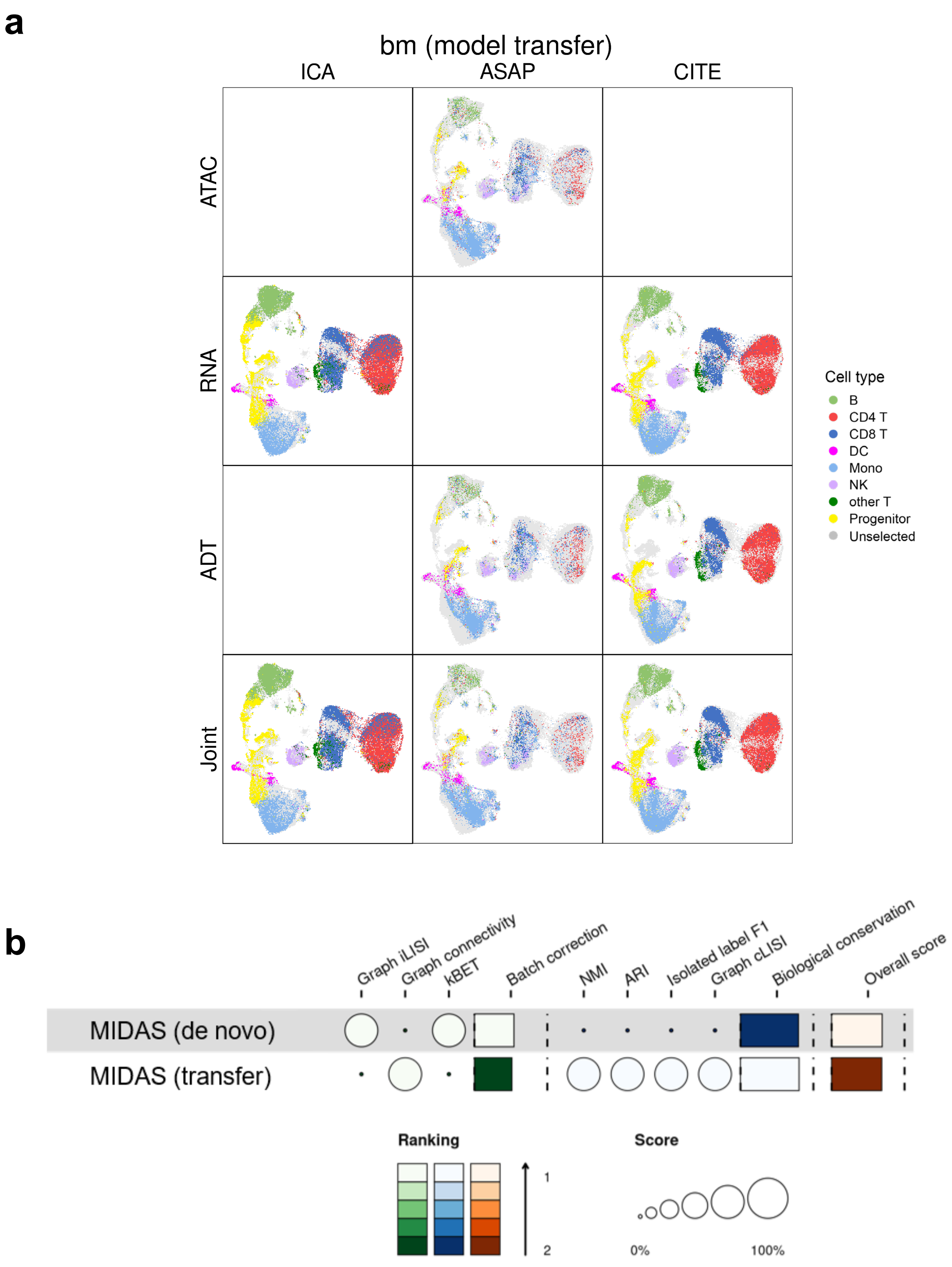

Supplementary Figure 26. Performance of MIDAS on model transfer in BMMC mosaic dataset. a, UMAP visualization of the biological states inferred by the transfer-learned MIDAS on bm dataset. **b**, scIB benchmarking the performance of *de novo* and model transfer integration.

Supplementary Table 1. Public datasets used in this study.

Dataset	Batch	Protocal	Accession ID/URL	Filtered cell number	RNA	ADT	ATAC
DOGMA dataset	LLL Ctrl	DOGMA-seq	GSE166188 (GSM5065524, GSM5065525, GSM5065526)	7,361	√	√	√
	LLL Stim	DOGMA-seq	GSE166188 (GSM5065527, GSM5065528, GSM5065529)	5,897	√	√	√
	DIG Ctrl	DOGMA-seq	GSE166188 (GSM5065530, GSM5065531, GSM5065532)	10,190	√	√	√
	DIG Stim	DOGMA-seq	GSE166188 (GSM5065533, GSM5065534, GSM5065535)	9,527	√	√	√
TEA dataset	W1	TEA-seq	GSE158013 (GSM4949911)	7,325	√	√	√
	W3	TEA-seq	GSE158013 (GSM5123951)	6,587	√	√	√
	W4	TEA-seq	GSE158013 (GSM5123952)	6,897	√	√	√
	W5	TEA-seq	GSE158013 (GSM5123953)	6,910	√	√	√
	W6	TEA-seq	GSE158013 (GSM5123954)	7,137	√	√	√
TEA Multiome dataset	W1	10x Chromium Single Cell Multiome ATAC + Gene Expression	GSE158013 (GSM5123949)	6,096	√		√
	W2	10x Chromium Single Cell Multiome ATAC + Gene Expression	GSE158013 (GSM5123950)	7,284	√		√
10X Multiome dataset	ChrX	10x Chromium Single Cell Multiome ATAC + Gene Expression	https://www.10xgenomics.com/resources/datasets/10-k-human-pbm-cs-multiome-v-1-0-chromium-x-1-standard-2-0-0	9,868	√		√
	ChrC	10x Chromium Single Cell Multiome ATAC + Gene Expression	https://www.10xgenomics.com/resources/datasets/10-k-human-pbm-cs-multiome-v-1-0-chromium-controller-1-standard-2-0-0	9,582	√		√
	ARC2 10K	10x Chromium Single Cell Multiome ATAC + Gene Expression	https://www.10xgenomics.com/resources/datasets/pbmc-from-a-healthy-donor-no-cell-sorting-10-k-1-standard-2-0-0	11,116	√		√
	ARC2 3K	10x Chromium Single Cell Multiome ATAC + Gene Expression	https://www.10xgenomics.com/resources/datasets/pbmc-from-a-healthy-donor-no-cell-sorting-3-k-1-standard-2-0-0	2,566	√		√
ASAP dataset	Ctrl	ASAP-seq	GSE156473 (GSM4732109, GSM4732110)	4,255		√	√
	Stim	ASAP-seq	GSE156473 (GSM4732111, GSM4732112)	5,241		√	√
ASAP CITE dataset	Ctrl	CITE-seq	GSE156473 (GSM4732113, GSM4732114)	5,086	√	√	
	Stim	CITE-seq	GSE156473 (GSM4732115, GSM4732116)	3,629	√	√	

WNN CITE dataset	P1_0	CITE-seq	https://atlas.fredhutch.org/nygc/multimodal-pbmc/	6,378	√	√	
	P2_0	CITE-seq	https://atlas.fredhutch.org/nygc/multimodal-pbmc/	5,899	√	√	
	P3_0	CITE-seq	https://atlas.fredhutch.org/nygc/multimodal-pbmc/	4,628	√	√	
	P4_0	CITE-seq	https://atlas.fredhutch.org/nygc/multimodal-pbmc/	5,285	√	√	
	P5_0	CITE-seq	https://atlas.fredhutch.org/nygc/multimodal-pbmc/	6,952	√	√	
	P6_0	CITE-seq	https://atlas.fredhutch.org/nygc/multimodal-pbmc/	6,060	√	√	
	P7_0	CITE-seq	https://atlas.fredhutch.org/nygc/multimodal-pbmc/	8,854	√	√	
	P8_0	CITE-seq	https://atlas.fredhutch.org/nygc/multimodal-pbmc/	8,908	√	√	
BMMC mosaic dataset	ICA	10X v2 Sequencing	https://www.dropbox.com/s/xe5tithw1xjxrfs/ica_bone_marrow.h5?dl=0	34,592	√		
	ASAP	ASAP-seq	GSE156477	10,671		√	√
	CITE	CITE-seq	GSE128639	26,286	√	√	

Supplementary Table 2. Construction of rectangular and mosaic datasets with trimodal data.

**Data**	**Batch**	**Rectangular dataset**			**Mosaic dataset**																				
		full			paired+full			paired-abc			paired-ab			paired-ac			paired-bc			diagonal+full			diagonal		
		ATAC	RNA	ADT	ATAC	RNA	ADT	ATAC	RNA	ADT	ATAC	RNA	ADT	ATAC	RNA	ADT	ATAC	RNA	ADT	ATAC	RNA	ADT	ATAC	RNA	ADT
DOGMA	LLL Ctrl	√	√	√	√	√		√	√		√	√		√	√		√		√	√			√		
	LLL Stim	√	√	√	√		√		√	√	√		√		√	√		√	√		√			√	
	DIG Ctrl	√	√	√		√	√	√		√	√	√		√	√		√		√			√			√
	DIG Stim	√	√	√	√	√	√		√	√	√		√		√	√		√	√	√	√	√			√
TEADOG	W1	√	√	√	√	√		√	√		√	√		√	√		√		√	√			√		
	W6	√	√	√	√		√		√	√	√		√		√	√		√	√		√			√	
	LLL ctrl	√	√	√		√	√	√		√	√	√		√	√		√		√			√			√
	DIG stim	√	√	√	√	√	√		√	√	√		√		√	√		√	√	√	√	√			√

*For datasets starting with "paired-", "a" denotes ATAC+RNA, "b" denotes ATAC+ADT, and "c" denotes RNA+ADT.

Supplementary Table 3. An overview of compared methods for single-cell multimodal integration.

**Method**			**Trimodal task (ATAC+RNA+ADT)**								**Bimodal task (ATAC+RNA)**					**Bimodal task (RNA+ADT)**				
**Integration type**		**Name**	**full**	**paired +full**	**paired -abc**	**paired -ab**	**paired -ac**	**paired -bc**	**diag. +full**	**diag.**	**paired**	**atac+ paired**	**rna+ paired**	**diag.+ paired**	**diag.**	**paired**	**rna+ paired**	**adt+ paired**	**diag.+ paired**	**diag.**
Trimodal (ATAC+RNA+ ADT)	Mosaic	MIDAS	√	√	√	√	√	√	√	√	√	√	√	√	√	√	√	√	√	√
		Multigrate	√	√	√	√	√	√	√	√										
		scMoMaT	√	√	√	√	√	√	√	√										
		scVAEIT	√	√	√	√	√	√	√	√										
		StabMap	√	√	√	√	√	√	√	√										
	Rectangular	BBKNN+average	√																	
		Harmony+WNN	√																	
		LIGER+WNN	√																	
		MOFA+	√																	
		PCA+WNN	√																	
		Scanorama-embed+WNN	√																	
		Scanorama-feat+WNN	√																	
		Seurat-CCA+WNN	√																	
		Seurat-RPCA+WNN	√																	
Bimodal (ATAC+RNA)	Mosaic	Cobolt									√	√	√	√						
		MultiVI									√	√	√	√						
	Diagonal	uniPort									√				√					
		GLUE													√					
Bimodal (RNA+ADT)	Mosaic	totalVI														√	√			
	Rectangular	sciPENN														√				

*Detailed configuration of each task can be found in Supplementary Tables 2, 7, and 8.

Supplementary Table 4. Metrics of scMIB.

	**Batch correction**	**Madality alignment**	**Biological conservation**
**Feature space**	Graph iLISI (feat)<br>Graph connectivity (feat)<br>kBET (feat)	ATAC AUROC<br>RNA Pearson's *r*<br>ADT Pearson's *r*	NMI (feat)<br>ARI (feat)<br>Graph cLISI (feat)<br>Isolated label F1 (feat)
**Embedding space**	Graph iLISI (embed)<br>Graph connectivity (embed)<br>kBET (embed)	Modality ASW<br>FOSCTTM<br>Label transfer F1	NMI (embed)<br>ARI (embed)<br>Isolated label F1 (embed)<br>Graph cLISI (embed)

Supplementary Table 5. Construction of cell type missing datasets.

**Original data**								
	**B**	**CD4 T**	**CD8 T**	**DC**	**Mono**	**NK**	**other**	**other T**
**LLL Ctrl**	296	4983	1435	43	53	166	21	364
**LLL Stim**	159	3974	1080	45	11	213	27	388
**DIG Ctrl**	463	6913	1713	57	112	298	23	611
**DIG Stim**	332	6377	1608	62	64	431	33	620
**Major cell type missing**								
	**B**	**CD4 T**	**CD8 T**	**DC**	**Mono**	**NK**	**other**	**other T**
**LLL Ctrl**	296	4983	1435	43	53	166	21	**0**
**LLL Stim**	**0**	3974	1080	45	11	213	27	388
**DIG Ctrl**	463	**0**	1713	57	112	298	23	611
**DIG Stim**	332	6377	**0**	62	64	431	33	620
**Minor cell type missing**								
	**B**	**CD4 T**	**CD8 T**	**DC**	**Mono**	**NK**	**other**	**other T**
**LLL Ctrl**	296	4983	1435	43	53	166	21	**0**
**LLL Stim**	159	3974	**0**	45	11	213	27	388
**DIG Ctrl**	463	6913	1713	57	112	**0**	23	611
**DIG Stim**	**0**	6377	1608	62	64	431	33	620

*Cell numbers of each batch belonging different cell types are presented.

Supplementary Table 6. Construction of datasets with different sequencing depth.

	**Original**	**D_0.5**	**D_0.2**	**D_0.1**	**D_0.05**	**D_0.02**
**LLL Ctrl**	1	0.5	0.2	0.1	0.05	0.02
**LLL Stim**	1	0.5	0.2	0.1	0.05	0.02
**DIG Ctrl**	1	1	1	1	1	1
**DIG Stim**	1	1	1	1	1	1

*Sampling ratios of each batch for different constructed datasets (D_0.5, D_0.2, D_0.1, D_0.05, D_0.02) are presented.

*Each modality of the original mosaic datasets was downsampled using the downsampleMatrix function (bycol = F) from the scuttle (v1.4.0) R package.

Supplementary Table 7. Construction of rectangular and mosaic datasets with ATAC+RNA bimodal data.

Data (ATAC+RNA)	Batch	Rectangular dataset		Mosaic dataset							
		paired		rna+paired		atac+paired		diagonal+paired		diagonal	
		RNA	ATAC	RNA	ATAC	RNA	ATAC	RNA	ATAC	RNA	ATAC
DOGMA	LLL Ctrl	√	√	√			√	√		√	
	LLL Stim	√	√	√	√	√	√		√		√
	DIG Ctrl	√	√	√			√	√		√	
	DIG Stim	√	√	√	√	√	√	√	√		√
TEADOG	W1	√	√	√			√	√		√	
	W6	√	√	√	√	√	√		√		√
	LLL ctrl	√	√	√			√	√		√	
	DIG stim	√	√	√	√	√	√	√	√		√

Supplementary Table 8. Construction of rectangular and mosaic datasets with RNA+ADT bimodal data.

Data (RNA+ADT)	Batch	Rectangular dataset		Mosaic dataset							
		paired		rna+paired		adt+paired		diagonal+paired		diagonal	
		RNA	ADT	RNA	ADT	RNA	ADT	RNA	ADT	RNA	ADT
DOGMA	LLL Ctrl	√	√	√			√	√		√	
	LLL Stim	√	√	√	√	√	√		√		√
	DIG Ctrl	√	√	√			√	√		√	
	DIG Stim	√	√	√	√	√	√	√	√		√
TEADOG	W1	√	√	√			√	√		√	
	W6	√	√	√	√	√	√		√		√
	LLL ctrl	√	√	√			√	√		√	
	DIG stim	√	√	√	√	√	√	√	√		√

Supplementary Table 9. Datasets used in atlas construction and knowledge transfer.

**Dataset**	**Batch**	**Modality**			**Cell number**	**Knowledge transfer (within-tissue)**		**Knowledge transfer (cross-tissue)**	
		**ATAC**	**RNA**	**ADT**		**Reference (atlas-no_dogma, PBMC)**	**Query (dogma, PBMC)**	**Reference (atlas, PBMC)**	**Query (bm, BMMC)**
BMMC mosaic dataset	ICA		√		34,592				√
	ASAP	√		√	10,671				√
	CITE		√	√	26,286				√
DOGMA dataset	LLL Ctrl	√	√	√	7,361		√	√	
	LLL Stim	√	√	√	5,897		√	√	
	DIG Ctrl	√	√	√	10,190		√	√	
	DIG Stim	√	√	√	9,527		√	√	
TEA dataset	W1	√	√	√	7,325	√		√	
	W3	√	√	√	6,587	√		√	
	W4	√	√	√	6,897	√		√	
	W5	√	√	√	6,910	√		√	
	W6	√	√	√	7,137	√		√	
TEA Multiome dataset	W1	√	√		6,096	√		√	
	W2	√	√		7,284	√		√	
10X Multiome dataset	ChrX	√	√		9,868	√		√	
	ChrC	√	√		9,582	√		√	
	ARC2 10K	√	√		11,116	√		√	
	ARC2 3K	√	√		2,566	√		√	
ASAP dataset	Ctrl	√		√	4,255	√		√	
	Stim	√		√	5,241	√		√	
ASAP CITE dataset	Ctrl		√	√	5,086	√		√	
	Stim		√	√	3,629	√		√	
WNN CITE dataset	P1_0		√	√	6,378	√		√	
	P2_0		√	√	5,899	√		√	
	P3_0		√	√	4,628	√		√	
	P4_0		√	√	5,285	√		√	
	P5_0		√	√	6,952	√		√	
	P6_0		√	√	6,060	√		√	
	P7_0		√	√	8,854	√		√	
	P8_0		√	√	8,908	√		√	
Total cells		134,510	236,900	175,963	257,067	152,543	32,975	185,518	71,549

Supplementary Table 10. Construction of 14 dogma mosaic query datasets for knowledge transfer.

**Batch**	**Dogma query datasets for knowledge transfer**																				
	full			paired+full			paired-abc			paired-ab			paired-ac			paired-bc			diagonal+full		
	ATAC	RNA	ADT	ATAC	RNA	ADT	ATAC	RNA	ADT	ATAC	RNA	ADT	ATAC	RNA	ADT	ATAC	RNA	ADT	ATAC	RNA	ADT
LLL Ctrl	√	√	√	√	√		√	√		√	√		√	√		√		√	√		
LLL Stim	√	√	√	√		√		√	√	√		√		√	√		√	√		√	
DIG Ctrl	√	√	√		√	√	√		√	√	√		√	√		√		√			√
DIG Stim	√	√	√	√	√	√		√	√	√		√		√	√		√	√	√	√	√

**Batch**	diagonal			paired-a			paired-b			paired-c			atac			rna			adt		
	ATAC	RNA	ADT	ATAC	RNA	ADT	ATAC	RNA	ADT	ATAC	RNA	ADT	ATAC	RNA	ADT	ATAC	RNA	ADT	ATAC	RNA	ADT
LLL Ctrl	√			√	√		√		√		√	√	√				√				√
LLL Stim		√		√	√		√		√		√	√	√				√				√
DIG Ctrl			√	√	√		√		√		√	√	√				√				√
DIG Stim			√	√	√		√		√		√	√	√				√				√

*For datasets starting with “paired-”, “a” denotes ATAC+RNA, “b” denotes ATAC+ADT, and “c” denotes RNA+ADT.

Supplementary Table 11. Functionality comparison of MIDAS and other mosaic integration methods.

Method	Diverse mosaic combination	Embedding space		Feature space		Knowledge transfer	Flexible knowledge transfer	General mosaic integration
		Batch correction	Modality alignment	Imputation	Batch correction			
Multigrate	√	√		√		√		
scMoMaT	√			√				
scVAEIT	√			√		√		
StabMap				√				
MIDAS	√	√	√	√	√	√	√	√

Supplementary Table 12. The layer sizes of each module in MIDAS.

<table>
<tr><th colspan="2">Module</th><th colspan="3">Layer size</th></tr>
<tr><td rowspan="4">Encoder</td><td>f^{ATAC}</td><td>D^{chr1}→128→32
⋯
D^{chr22}→128→32</td><td>32×22→1024</td><td rowspan="3">1024→128→68
(shared layers)</td></tr>
<tr><td>f^{RNA}</td><td colspan="2">D^{RNA}→1024</td></tr>
<tr><td>f^{ADT}</td><td colspan="2">D^{ADT}→1024</td></tr>
<tr><td>f^{s}</td><td colspan="3">D^{s}→16→16→68</td></tr>
<tr><td rowspan="4">Decoder</td><td>g^{ATAC}</td><td rowspan="3">34→128→1024
(shared layers)</td><td>1024→32×22</td><td>32→128→D^{chr1}
⋯
32→128→D^{chr22}</td></tr>
<tr><td>g^{RNA}</td><td colspan="2">1024→D^{RNA}</td></tr>
<tr><td>g^{ADT}</td><td colspan="2">1024→D^{ADT}</td></tr>
<tr><td>g^{s}</td><td colspan="3">2→16→16→D^{s}</td></tr>
<tr><td rowspan="4">Classifier</td><td>r</td><td colspan="3">32→128→64→D^{s}</td></tr>
<tr><td>r^{ATAC}</td><td colspan="3">32→128→64→D^{s}</td></tr>
<tr><td>r^{RNA}</td><td colspan="3">32→128→64→D^{s}</td></tr>
<tr><td>r^{ADT}</td><td colspan="3">32→128→64→D^{s}</td></tr>
</table>

Supplementary Table 13. The input layer sizes of MIDAS on different tasks.

Input size	Dataset				
	**dogma-full**	**teadog-full**	**atlas-no_dogma**	**atlas**	**bm**
D^{ATAC}	30,589	31,243	52,393	52,867	37,840
- D^{chr1}	3,165	3,210	5,318	5,361	3,692
- D^{chr2}	2,338	2,315	4,124	4,160	3,050
- D^{chr3}	1,764	1,748	3,234	3,270	2,473
- D^{chr4}	993	985	1,861	1,875	1,494
- D^{chr5}	1,407	1,370	2,503	2,535	1,917
- D^{chr6}	1,853	1,861	3,293	3,312	2,421
- D^{chr7}	1,466	1,473	2,552	2,591	1,894
- D^{chr8}	1,169	1,175	2,032	2,066	1,589
- D^{chr9}	1,211	1,258	2,106	2,131	1,544
- D^{chr10}	1,418	1,437	2,473	2,500	1,861
- D^{chr11}	1,600	1,670	2,761	2,774	2,019
- D^{chr12}	1,656	1,685	2,877	2,896	2,087
- D^{chr13}	620	606	1,120	1,127	800
- D^{chr14}	1,147	1,141	1,892	1,905	1,262
- D^{chr15}	1,026	1,042	1,692	1,708	1,248
- D^{chr16}	1,317	1,384	2,063	2,088	1,447
- D^{chr17}	1,929	2,073	3,142	3,162	2,024
- D^{chr18}	498	497	917	928	705
- D^{chr19}	1,986	2,176	2,980	3,001	1,988
- D^{chr20}	866	933	1,519	1,525	1,079
- D^{chr21}	391	379	644	654	437
- D^{chr22}	769	825	1,290	1,298	809
D^{RNA}	4,045	4,047	4,051	4,047	4,096
D^{ADT}	208	213	228	228	239
D^{s}	4	4	23	27	3

Supplementary Table 14. Hyperparameter settings for MIDAS training.

**Hyperparameter**	**Value**
Modality alignment loss weight α	50
Technical IB loss weight β^s	30
Biological IB loss weight β^x	4
Technical noise likelihood loss weight γ	1000
Classifier update number K	3
Training-validation ratio	95:5
Mini-batch size	256
Maximum number of training epochs	2000
Learning rate	0.0001
Hidden layer dropout rate	0.2
