# Supplementary material for: Mosaic integration and knowledge transfer of single-cell multimodal data with MIDAS
Source: Nat Biotechnol. 2024 Jan 23;42(10):1594–605. doi: 10.1038/s41587-023-02040-y (PMC11471558; doi:10.1038/s41587-023-02040-y)
Supplement: Supplementary file 2 — Reporting Summary [file 41587_2023_2040_MOESM2_ESM.pdf]

nature portfolio

Corresponding author(s): Xiaochen Bo, Xiaomin Ying

Last updated by author(s): Jan 12, 2024

Reporting Summary

Nature Portfolio wishes to improve the reproducibility of the work that we publish. This form provides structure for consistency and transparency in reporting. For further information on Nature Portfolio policies, see our Editorial Policies and the Editorial Policy Checklist.

Statistics

For all statistical analyses, confirm that the following items are present in the figure legend, table legend, main text, or Methods section.

n/a	Confirmed	
☐	☒	The exact sample size (*n*) for each experimental group/condition, given as a discrete number and unit of measurement
☐	☒	A statement on whether measurements were taken from distinct samples or whether the same sample was measured repeatedly
☐	☒	The statistical test(s) used AND whether they are one- or two-sided<br>*Only common tests should be described solely by name; describe more complex techniques in the Methods section.*
☒	☐	A description of all covariates tested
☒	☐	A description of any assumptions or corrections, such as tests of normality and adjustment for multiple comparisons
☐	☒	A full description of the statistical parameters including central tendency (e.g. means) or other basic estimates (e.g. regression coefficient) AND variation (e.g. standard deviation) or associated estimates of uncertainty (e.g. confidence intervals)
☐	☒	For null hypothesis testing, the test statistic (e.g. *F*, *t*, *r*) with confidence intervals, effect sizes, degrees of freedom and *P* value noted<br>*Give P values as exact values whenever suitable.*
☒	☐	For Bayesian analysis, information on the choice of priors and Markov chain Monte Carlo settings
☒	☐	For hierarchical and complex designs, identification of the appropriate level for tests and full reporting of outcomes
☐	☒	Estimates of effect sizes (e.g. Cohen's *d*, Pearson's *r*), indicating how they were calculated

Our web collection on statistics for biologists contains articles on many of the points above.

Software and code

Policy information about availability of computer code

Data collection	All data used in this manuscript are publicly available and no software was used for data collection.
Data analysis	MIDAS was implemented using the Python (v3.8.8) package PyTorch (v2.0.0) with code available at https://github.com/labomics/midas.<br><br>Other software used in data preprocessing, algorithm comparison, performance evaluation, and result analysis include:<br><br>Python (3.8.8) software:<br>Arboreto (v0.1.5)<br>BBKNN (v1.5.1)<br>Cobolt (v1.0.1)<br>GLUE (v0.3.2)<br>MACS2 (v2.2.7.1)<br>Multigrate (v0.0.2)<br>PyTorch (v2.0.0)<br>Scanorama (v1.7.2)<br>Scanpy (v1.9.1)<br>scIB (v1.0.2)<br>scikit-learn (v1.2.2)<br>sciPENN (v1.0.0)<br>scMoMaT (v0.2.0)<br>scVAEIT (https://github.com/jaydu1/scVAEIT/tree/21541e58d97694abd9148963962024e4d8c2b997)

scvi-tools (v1.0.0)
umap-learn (v0.5.3)
uniPort (v1.2.2)

R (4.1.1) software:
clusterProfiler (v4.2.2)
Harmony (v0.1.1)
LIGER (v1.0.0)
MOFA+ (v1.4.0)
Monocle 3 (v1.3.1)
scuttle (v1.4.0)
Seurat (v4.3.0)
Signac (v1.9.0)
StabMap (v0.1.8)

For manuscripts utilizing custom algorithms or software that are central to the research but not yet described in published literature, software must be made available to editors and reviewers. We strongly encourage code deposition in a community repository (e.g. GitHub). See the Nature Portfolio guidelines for submitting code & software for further information.

Data

Policy information about availability of data

All manuscripts must include a data availability statement. This statement should provide the following information, where applicable:

- Accession codes, unique identifiers, or web links for publicly available datasets
- A description of any restrictions on data availability
- For clinical datasets or third party data, please ensure that the statement adheres to our policy

All datasets used in this study are already published and were obtained from public data repositories. See Supplementary Table 1 for detailed information on single-cell omics datasets used in this study, including access codes and URLs.

Human research participants

Policy information about studies involving human research participants and Sex and Gender in Research.

Reporting on sex and gender	Not relevant, because in our study we did not recruit any participants.
Population characteristics	Not relevant, because in our study we did not recruit any participants.
Recruitment	Not relevant, because in our study we did not recruit any participants.
Ethics oversight	Not relevant, because in our study we did not recruit any participants.

Note that full information on the approval of the study protocol must also be provided in the manuscript.

Field-specific reporting

Please select the one below that is the best fit for your research. If you are not sure, read the appropriate sections before making your selection.

☒ Life sciences ☐ Behavioural & social sciences ☐ Ecological, evolutionary & environmental sciences

For a reference copy of the document with all sections, see nature.com/documents/nr-reporting-summary-flat.pdf

Life sciences study design

All studies must disclose on these points even when the disclosure is negative.

Sample size	No sample size calculation was performed. To prevent individual datasets from dominating the integration results, the WNN CITE dataset and the ICA batch of the BMMC mosaic dataset were simply selected from the original data without using any statistical methods (Methods). For other data used in the paper (Methods and Supplementary Table 1), sample sizes were chosen based on the availability of public data resources and all available data were included for analysis.
Data exclusions	Standard filtering procedures were applied to exclude low-quality cells. The details are can be found in the Methods section.
Replication	We provide the code necessary for replicating the results. Different package versions or computational environments might lead to slightly different outputs.
Randomization	Random train/test set splits were used when training models in order to evaluate them, as common practice.

Blinding	Blinding is not relevant, because our study did not involve group allocation that requires blinding.

Reporting for specific materials, systems and methods

We require information from authors about some types of materials, experimental systems and methods used in many studies. Here, indicate whether each material, system or method listed is relevant to your study. If you are not sure if a list item applies to your research, read the appropriate section before selecting a response.

Materials & experimental systems

n/a	Involved in the study
☒	☐ Antibodies
☒	☐ Eukaryotic cell lines
☒	☐ Palaeontology and archaeology
☒	☐ Animals and other organisms
☒	☐ Clinical data
☒	☐ Dual use research of concern

Methods

n/a	Involved in the study
☒	☐ ChIP-seq
☒	☐ Flow cytometry
☒	☐ MRI-based neuroimaging
